# S100A8/A9^hi^ neutrophils induce mitochondrial dysfunction and PANoptosis in endothelial cells via mitochondrial complex I deficiency during sepsis

**DOI:** 10.1038/s41419-024-06849-6

**Published:** 2024-06-28

**Authors:** Yanghanzhao Wang, Yuxin Shi, Yuwen Shao, Xihua Lu, Hao Zhang, Changhong Miao

**Affiliations:** 1 Department of Anesthesiology, Zhongshan Hospital, Fudan University, Shanghai, China; 2 Shanghai Key Laboratory of Perioperative Stress and Protection, Shanghai, China; 3 Department of Anesthesiology, Shanghai Medical College, Fudan University, Shanghai, China; 4 Department of Anesthesiology, Affiliated Cancer Hospital of Zhengzhou University, Henan Cancer Hospital, Zhengzhou, China

**Keywords:** Mitophagy, Bacterial infection

## Abstract

S100a8/a9, largely released by polymorphonuclear neutrophils (PMNs), belongs to the S100 family of calcium-binding proteins and plays a role in a variety of inflammatory diseases. Although S100a8/a9 has been reported to trigger endothelial cell apoptosis, the mechanisms of S100a8/a9-induced endothelial dysfunction during sepsis require in-depth research. We demonstrate that high expression levels of S100a8/a9 suppress Ndufa3 expression in mitochondrial complex I via downregulation of Nrf1 expression. Mitochondrial complex I deficiency contributes to NAD^+^-dependent Sirt1 suppression, which induces mitochondrial disorders, including excessive fission and blocked mitophagy, and mtDNA released from damaged mitochondria ultimately activates ZBP1-mediated PANoptosis in endothelial cells. Moreover, based on comprehensive scRNA-seq and bulk RNA-seq analyses, S100A8/A9^hi^ neutrophils are closely associated with the circulating endothelial cell count (a useful marker of endothelial damage), and S100A8 is an independent risk factor for poor prognosis in sepsis patients.

## Introduction

Sepsis is a persistent systemic inflammatory condition caused by an extreme immune response to infection, and it is always accompanied by multiple organ dysfunction [1]. To date, sepsis and septic shock have remained leading causes of death in critically ill patients, and the mortality rate of 30-day septic shock is as high as 34.7% [2]. In the initial stage of infection, the endothelial barrier and antimicrobial substances released by immune cells can cooperatively hinder pathogen dissemination [3, 4]. However, overwhelming amounts of inflammatory mediators damage endothelial barrier integrity, which contributes to microcirculatory disturbance and end-organ injury, especially lung injury [5, 6].

During sepsis, neutrophils are often the first immune cells to be recruited to infected sites [7]. Calprotectin (S100a8/a9), a heterodimeric Ca^2+^-binding protein mainly released by neutrophils, is thought to induce prolonged inflammation and endothelial injury through binding with its receptors, including toll-like receptor 4 (TLR4) and receptor for advanced glycation end products (RAGE) [8–10]. According to previous studies, inflammatory mediators, such as S100a8/a9 and neutrophil extracellular traps (NETs, web-like DNA structures adorned with bactericidal proteins), tend to induce mitochondrial metabolic disturbance and disrupt mitochondrial homeostasis [11, 12].

Mitochondrial dynamics (fission and fusion) and mitophagy are vital for maintaining mitochondrial quantity and quality. Specifically, fusion allows mitochondria to transfer gene products for optimal function [13], while fission induces the isolation of impaired mitochondria for degradation through mitophagy (a selective form of autophagy) [14]. Failure at any stage results in the accumulation of damaged mitochondria in the cytoplasm and ultimately cell death.

Previously, the different cell death patterns were considered independent. However, increasing evidence emphasizes the extensive crosstalk among different cell death patterns, and these pathways may be intertwined. Therefore, a novel form of cell death, PANoptosis, was proposed in 2019 [15]. It is a coordinated cell death pattern that involves apoptosis, pyroptosis and necroptosis [16]. Several studies have shown that Z-DNA binding protein 1 (ZBP1) can sense cytosolic DNA and consequently activate PANoptosis [17].

Here, our study provides evidence that S100A8/A9^hi^ neutrophils are present specifically in lung tissues from septic mice. High expression levels of S100a8/a9 induce mitochondrial disorders in endothelial cells, including excessive fission and blocked mitophagy, mainly through Ndufa3 suppression in mitochondrial complex I. Finally, mtDNA released from damaged mitochondria activates ZBP1-mediated PANoptosis.

## Results

### Increased numbers of S100A8/A9^hi^ neutrophils are found in the lung tissues of sepsis model mice, and these cells exhibit enhanced interactions with endothelial cells

**Fig. 1:**
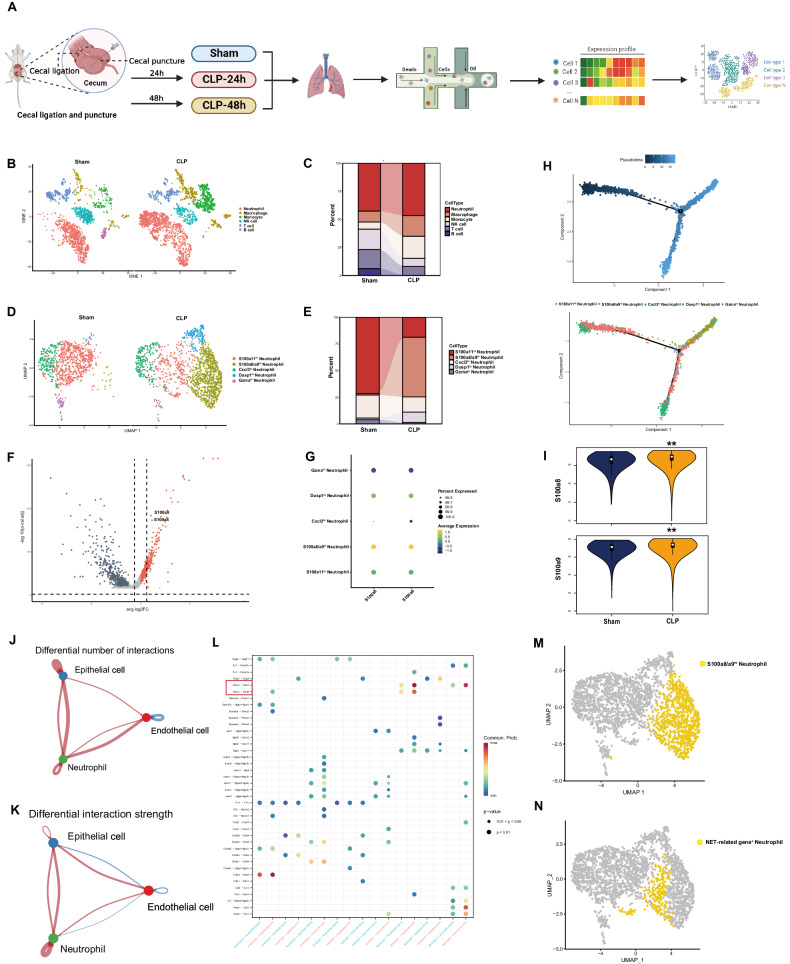
Increased numbers of S100A8/A9^hi^ neutrophils exist in lung tissues from CLP mice, and showed enhanced interactions with endothelial cells. **A** The main workflow of the scRNA-seq; **B** The UMAP plot was based on scRNA-seq data, and it showed five identified immune cell types; **C** The sankey diagram showed the percentages of five immune cells in sham and CLP groups; **D** Five clusters of neutrophils were identified in the UMAP plot; **E** The changes of five neutrophil subclusters percentage were shown on the sankey diagram; **F** Volcano map showed upregulated genes in S100A8/A9^hi^ neutrophils compared with other subpopulations; **G** The expression levels of S100A8 and S100A9 in five neutrophil subclusters on dot plot; **H** The prediction of neutrophil differentiation trajectories using pseudotime analysis; **I** The expression levels of S100A8 and S100A9 of neutrophils in sham and CLP groups; **J**, **K** The number and strength of interactions among neutrophils, endothelial cells and epithelial cells analyzed by CellChat; **L** The expression levels of ligand-receptor pairs analyzed by CellChat (Since there is no ligand-receptor pair with high expression among epithelial cells in sham group, the dot plot only shows the expressions of ligand-receptor pairs among epithelial cells in CLP group.); **M**, **N** The distribution of S100A8/A9^hi^ neutrophils and NET-related gene^+^ neutrophils on the UMAP plot. Wilcoxon rank sum test was used for the comparison between two groups. *p < 0.05, **p < 0.01 versus sham group.

Since the lung is considered as one of the most susceptible organs to sepsis [18], we selected publicly available scRNA-seq data from the lung tissues of sham and CLP mice for analysis (Fig. 1A). First, to explore the mechanisms underlying the excessive immune response to infection, all immune cells were clustered and identified by their marker genes (Fig. 1B, Supplementary Table 4). According to the relative percentages of each cell type, neutrophils accounted for the largest proportion of immune cells and were considerably more abundant in the CLP group (Fig. 1C). Therefore, neutrophils were extracted and reclustered into five subgroups. We found that a special subgroup, which showed greater expression of S100A8 and S100A9 than the other groups, was present only in the lung tissues of septic mice and represented a large proportion of the total neutrophil population (Fig. 1D–G, Supplementary Fig. 1A−D). Additionally, pseudotime analysis revealed that S100A8/A9^hi^ neutrophils were specifically in the late stage of differentiation, indicating that neutrophils were induced to differentiate into S100A8/A9^hi^ neutrophils during sepsis progression (Fig. 1H). Due to the presence of this subpopulation, neutrophils in the CLP group exhibited increased expressions of S100A8 and S100A9 and exhibited increased immune function and metabolism (Fig. 1I, Supplementary Fig. 1E–K).

Previously, we demonstrated that neutrophils could disturb the metabolism of endothelial cells and alveolar epithelial cells to aggravate sepsis-induced acute lung injury (SI-ALI) [11, 19]. Consequently, we next explored the interactions among neutrophils, endothelial cells and epithelial cells by using the “CellChat” R package. The results indicated that the number of interactions among these three cell types increased (Fig. 1J). Notably, from an interaction strength perspective, neutrophils showed enhanced unidirectional effects on endothelial cells, and endothelial cells also affected epithelial cells unidirectionally (Fig. 1K). These findings suggested that endothelial cells might act as a “bridge” in neutrophil-induced epithelial cell damage. We then focused on the ligand‒receptor pairs with upregulated expression in neutrophils and endothelial cells. The results indicated that thrombospondin 1 (Thbs1)-CD47 and Thbs1-CD36 expressions were clearly upregulated (Fig. 1L). Previous studies have revealed that endothelial cells exhibit increased Thbs1 expression after treatment with S100a8/a9 [20]. The above results suggest that neutrophils induce endothelial damage mainly through the release of S100a8/a9.

In addition, our previous study revealed that neutrophil extracellular traps (NETs) released by neutrophils might also damage the endothelial barrier during sepsis [21]. Based on the 137 identified NET formation-related genes, GSEA suggested that neutrophils from the sepsis model mice were able to release more NETs [22] (Supplementary Fig. 1L, Supplementary Table 5). Moreover, the subpopulation with high expression of NET-related genes was mostly composed of S100A8/A9^hi^ neutrophils (Fig. 1M, N, Supplementary Fig. 1M-N). These results indicate that S100A8/A9^hi^ neutrophils, including those in the NET-related gene^+^ subgroup, play a critical role in endothelial injury during sepsis.

In conclusion, the results of the scRNA-seq data analysis suggest that the presence of S100A8/A9^hi^ neutrophils, which exist especially in the lung tissues of septic model mice, might induce endothelial barrier damage to exacerbate lung injury during sepsis.

### High expression levels of S100a8/a9 induce excessive inflammatory responses and acute lung injury, both of which are reversed by an S100a8/a9 inhibitor

**Fig. 2:**
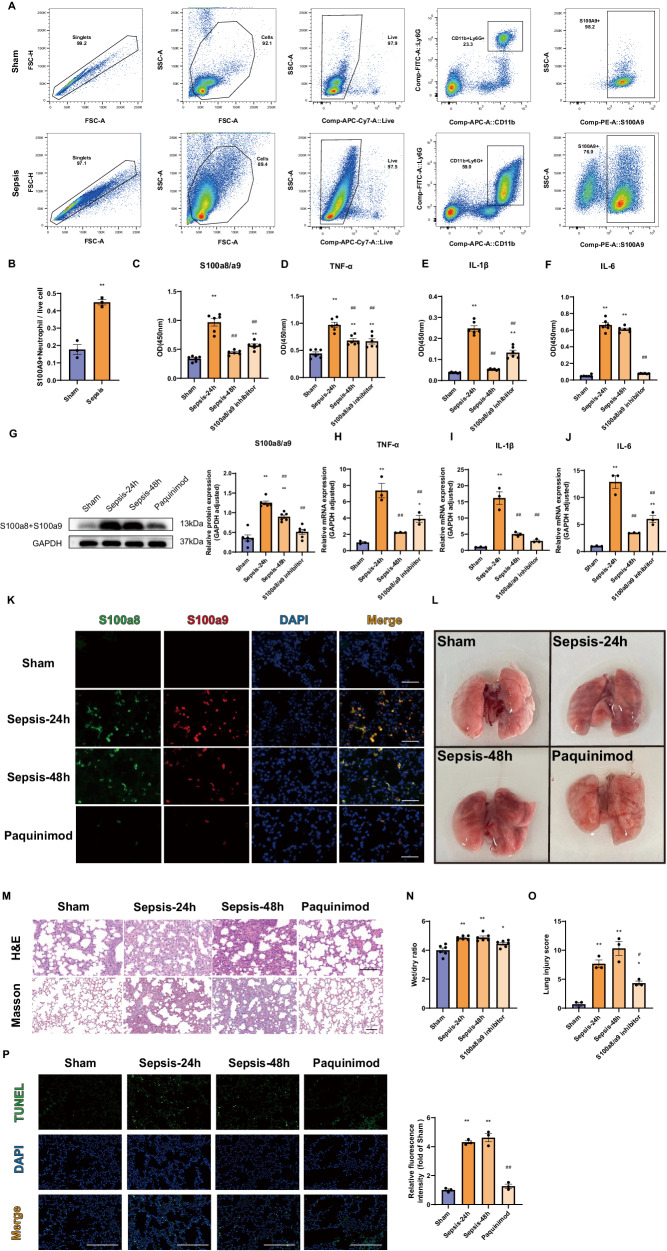
S100a8/a9 induces overwhelming inflammatory responses and acute lung injury, both of which could be alleviated by S100a8/a9 inhibitor. **A**, **B** The changes of S100A9^+^ neutrophil proportion were analyzed by flow cytometry (*n* = 3 in each group); **C**–**F** ELISA results showed the variations of S100a8/a9 and inflammatory cytokines concentrations in serum (*n* = 6 in each group). **G** The expression of S100a8 + S100a9 protein in lung tissues measured by Western blot (*n* = 6 in each group. The antibody we used is Rabbit recombinant multiclonal [RM1038] to S100A8 + S100A9 (Abcam; ab288715). The observed band size of S100A8 + S100A9 is 11, 14 kDa. But in some cell types, only one band can be observed, and the band size of S100A8 + S100A9 is 13 kDa approximately.); **H**–**J** The mRNA expression levels of inflammatory mediators detected by RT-qPCR (*n* = 3 in each group); **K** Representative immunofluorescence images showed S100a8/a9 expression in lung tissues; Scale bar: 40 µm; **L** Photos of dissected lung from mice; **M** Images of H&E and Masson staining of lung tissues to assess the degree of lung injury and fibrosis; Scale bar: 100 µm; **N** Wet/dry ratio was used to evaluate the extent of lung edema (*n* = 6 in each group); **O** The degree of lung injury was measured by lung injury score (*n* = 3 in each group); **P** Images of TUNEL staining of lung tissues were used to assess cell apoptosis; Scale bar: 40 µm (*n* = 3 in each group). Each bar showed means ± SEM. Unpaired *t-*test was used for the comparison between two groups. Comparison among three or more groups was analyzed by one-way ANOVA. *p < 0.05, **p < 0.01 versus sham group. ^#^p < 0.05, ^##^p < 0.01 versus sepsis-24h group.

Since S100a8/a9 usually exists as a dimer, we evaluated the number of S100A9^+^ neutrophils by flow cytometry. The results demonstrated that the percentage of S100A9^+^ neutrophils in the peripheral blood were significantly increased in septic mice compared to control mice (Fig. 2A, B). According to previous studies, the peak of lung vascular injury occurred at 24 h after CLP [23]. And based on the results of murine sepsis score (MSS) and survival analysis, septic model mice had a very high sepsis injury score (MSS ≥ 14) at 24 h post CLP, and the mortality rate also significantly increased at 24 h (Supplementary Fig. 2B-C). The ELISA results also indicated that the levels of S100a8/a9 and inflammatory cytokines increased considerably at 24 h. However, administration of the S100a8/a9 inhibitor paquinimod significantly inhibited the increase in S100a8/a9 expression and inflammatory mediator levels at 24 h after CLP (Fig. 2C–F). Endothelial barrier damage further induces end-organ injury. H&E staining of tissues revealed that the lung was more vulnerable to acute infection than other organs, such as the liver, kidney, spleen and intestine (Fig. 2M, Supplementary Fig. 2A). Treatment with the S100a8/a9 inhibitor noticeably ameliorated the degree of lung injury, although it had no significant impact on the 72 h survival rate (Fig. 2M, O, Supplementary Fig. 2C).

Therefore, we next evaluated lung inflammatory lesions. Based on the Western blot, RT‒qPCR and immunofluorescence results, S100a8/a9, accompanied by several inflammatory cytokines, mainly accumulated in lung tissues in the CLP-24h group. Consistent with previous observations, the use of paquinimod mitigated these inflammatory responses (Fig. 2G–K). Images of lung tissues also indicated the protective effect of the S100a8/a9 inhibitor (Fig. 2L). Moreover, the results of Masson staining, wet/dry ratio and TUNEL staining further confirmed the more severe lung injury in 24h-CLP group. And the S100a8/a9 inhibitor could considerably alleviate pulmonary fibrosis and cell apoptosis (Fig. 2M, N, P).

Collectively, these findings confirm the critical role of S100a8/a9 in sepsis-induced lung injury. An S100a8/a9 inhibitor might be a potential therapeutic strategy.

### High expression levels of S100a8/a9 induce endothelial cell apoptosis by inhibiting the Erk signaling pathway

**Fig. 3:**
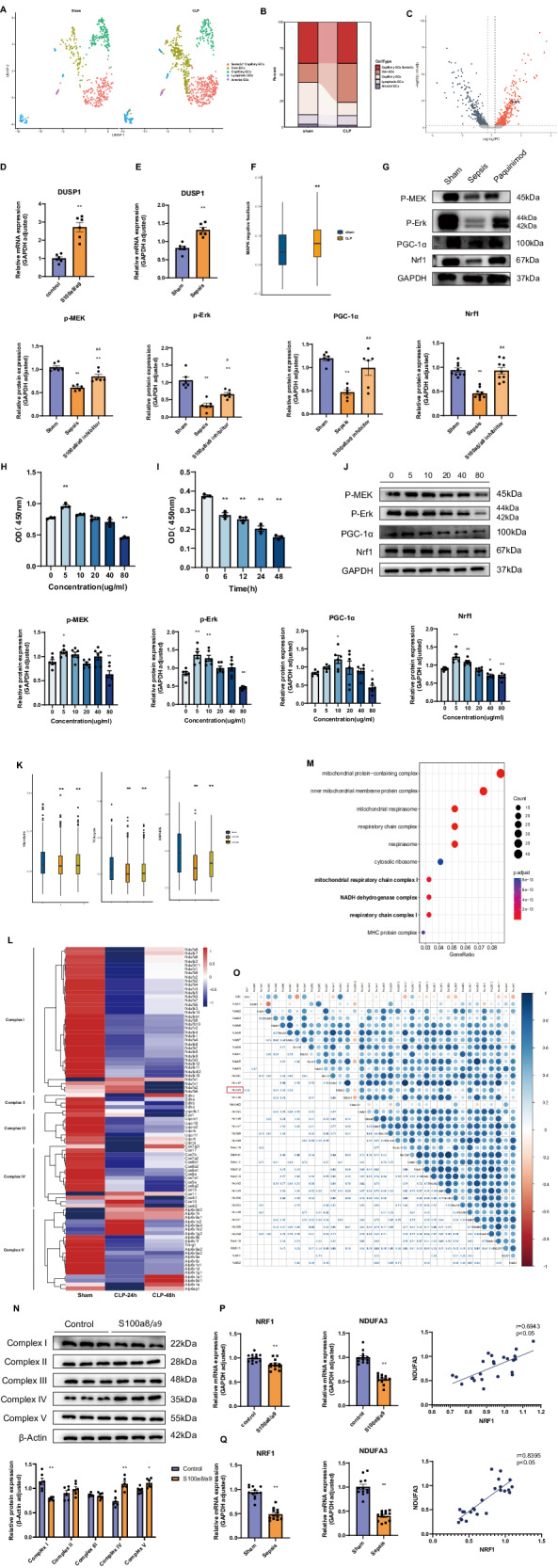
High expression levels of S100a8/a9 inhibit viability of endothelial cells via induction of metabolic disorders. **A** Five types of endothelial cells were showed on the UMAP plot; **B** The sankey diagram showed the percentages of five types of endothelial cells in sham and CLP groups; **C**. The volcano map showed the noticeably upregulated genes in capillary endothelial cells; **D**, **E** The mRNA expression levels of DUSP1 detected by RT-qPCR in cell and animal experiments (*n* = 6 in each group); **F** The extent of MAPK negative feedback was evaluated by GSVA enrichment analysis; **G** Western blot analysis was used to assess Erk signaling pathway in lung tissues from mice (*n* = 6 in each group); **H**, **I** The viability of endothelial cells was evaluated by CCK8 (*n* = 3 in each group); **J** The protein expression levels of Erk signaling pathway were assessed by Western blot (*n* = 6 in each group); **K** Metabolic pathways in endothelial cells from mice were evaluated by GSVA enrichment analysis; **L** Heatmap showed the expression levels of mitochondrial complexes-related genes in endothelial cells from mice. **M** The significantly downregulated cell components were showed on the dot plot; **N** The protein expression levels of mitochondrial complexes were assessed by Western blot (*n* = 6 in each group); **O** Corrplot R package was used to evaluate the correlation between NRF1 and complex I-related genes in every subcluster of endothelial cell; **P**, **Q** The mRNA levels of NRF1 and NDUFA3 were measured by RT-qPCR in cell and animal experiments (*n* = 12 in each group), and the correlation between NRF1 and NDUFA3 was shown on the correlation curve (*n* = 24). Each bar showed means ± SEM. Unpaired *t-*test was used for the comparison between two groups. Comparison among three or more groups was analyzed by one-way ANOVA. *p < 0.05, **p < 0.01 versus control group. ^#^p < 0.05, ^##^p < 0.01 versus sepsis group.

After we observed enhanced interactions between neutrophils and endothelial cells, the endothelial cell subpopulation was extracted for further analysis. On the basis of marker genes, endothelial cells were reclustered into five subgroups (Fig. 3A). We found that the percentage of capillary endothelial cells noticeably decreased, indicating that this type of endothelial cell was severely damaged (Fig. 3B). Moreover, capillary injury usually contributes to microcirculation dysfunction and organ damage [24]. Therefore, we focused on genes with significantly upregulated expression and related signaling pathways. The volcano map indicated the remarkable upregulation of dual-specificity phosphatase 1 (DUSP1) expression in capillary endothelial cells (Fig. 3C). And this result was further confirmed by cell and animal experiments (Fig. 3D, E). In the early stage of sepsis, the endothelium is activated, and inflammatory angiogenesis is induced in an extracellular signal-regulated kinase (Erk/MAPK)-dependent manner [25]. However, dual-specificity phosphatases (DUSPs) are considered to reduce phospho-Erk (p-Erk) expression levels and constitute a negative feedback loop when the Erk signaling pathway is overactivated [26]. GSEA enrichment results of DUSP genes indicated that the Erk signaling pathway was inhibited (Fig. 3F, Supplementary Table 6), which means that endothelial cells might shift to a pro-apoptotic phenotype from a proliferative status. Moreover, inhibition of the Erk pathway might contribute to mitochondrial dysfunction by suppressing the expression of PGC-1α and its nuclear receptor Nrf1 [27, 28]. The results of animal experiments demonstrated that the Erk signaling pathway showed an obvious downward trend in activation in lung tissues from the CLP group, while this downregulation could be reversed by using an S100a8/a9 inhibitor (Fig. 3G). CCK-8 assays showed that low concentrations of S100a8/a9 stimulated endothelial cell proliferation, while high concentrations of S100a8/a9 induced apoptosis in vitro (Fig. 3H). At a concentration of 80 µg/ml, S100a8/a9 inhibited cell viability in a time-dependent manner (Fig. 3I). In addition, Western blot analysis also revealed that the Erk signaling pathway was first activated and then suppressed with increasing S100a8/a9 concentrations (Fig. 3J). In summary, these findings indicate that high expression levels of S100a8/a9 could induce endothelial cell apoptosis via Erk signaling pathway inhibition. The suppression of PGC-1α/Nrf1 activity in the nucleus might result in mitochondrial dysfunction.

### S100a8/a9 induces endothelial cell metabolic disorders by suppressing NDUFA3 expression in mitochondrial complex I

Since Nrf1 is generally considered to maintain mitochondrial homeostasis and regulate metabolism [29], we analyzed several metabolic pathways in endothelial cells. GSVA revealed no significant difference in glycolysis, but the activities of the tricarboxylic acid (TCA) cycle and of oxidative phosphorylation (OXPHOS) in mitochondria decreased considerably in the CLP-24h group (Fig. 3K, Supplementary Table 7). We next assessed the expression levels of genes that encode five mitochondrial complexes and found that these genes exhibited different degrees of expression downregulation in endothelial cells (Fig. 3L). Moreover, enrichment analysis of the genes with downregulated expression suggested that mitochondrial complex I was significantly inhibited during sepsis (Fig. 3M). Consistent with the sequencing data, the protein expression level of complex I decreased markedly after the administration of S100a8/a9 (Fig. 3N). According to previous studies, Nrf1 was known to regulate mitochondrial complexes-related genes directly or indirectly [12, 30, 31]. Since mitochondrial complex I is encoded by several genes, we next explored which target gene is most relevant to Nrf1. Spearman’s correlation was used to analyze the correlation between expression of the NRF1 gene and that of 37 complex I-related genes, and the results indicated a strong correlation between NRF1 and NDUFA3 expression (Fig. 3O). Additionally, RT‒qPCR analysis confirmed that the expression levels of NRF1 and NDUFA3 were markedly downregulated in S100a8/a9-treated endothelial cells and in septic model mice. Moreover, it further confirmed a positive correlation between these two genes (Fig. 3P, Q). Taken together, these results confirm that S100a8/a9 mainly suppresses mitochondrial complex I via Nrf1 inhibition, and the gene NDUFA3 strongly correlates with NRF1.

### S100a8/a9 induces mitochondrial dysfunction, and mitochondrial complex I deficiency further downregulates NAD^+^-dependent Sirt1 expression

**Fig. 4:**
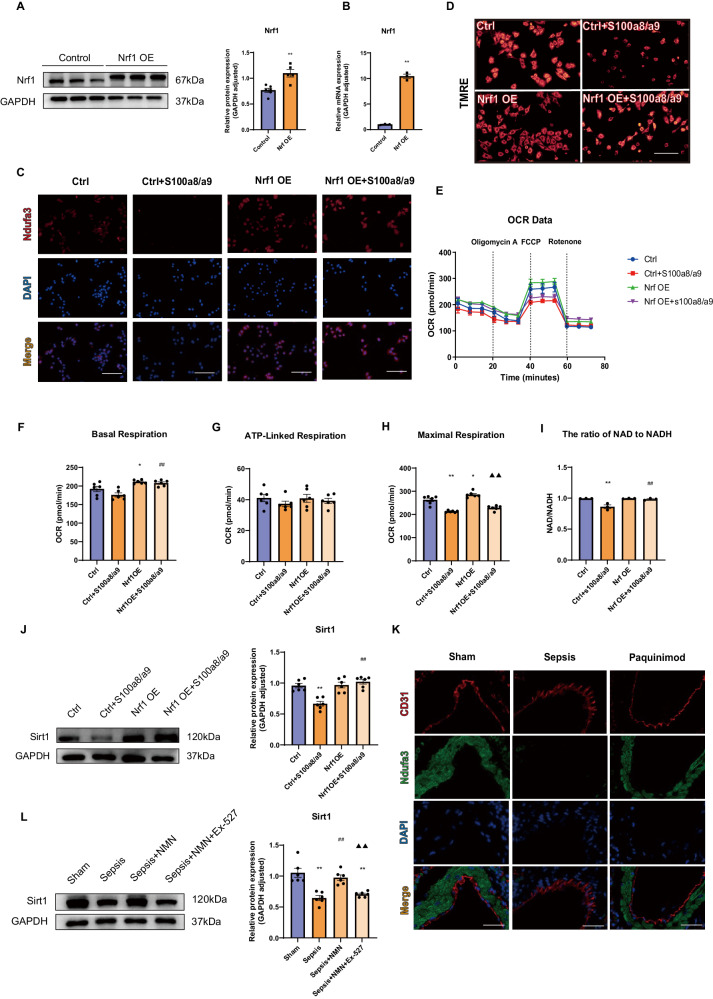
S100a8/a9 induces mitochondrial dysfunction and NAD^+^-dependent Sirt1 downregulation. **A**, **B** NRF1-overexpressing endothelial cells were successfully constructed according to the results of Western blot (*n* = 5 in each group) and RT-qPCR (*n* = 3 in each group). **C** Images from immunofluorescence assay showed the Ndufa3 expression in endothelial cells; Scale bar: 200 µm; **D** Mitochondrial membrane potential was measured by TMRE kit; Scale bar: 40 µm; **E–H** The OCR was measured by an XF96 Extracellular Flux Analyser (Seahorse Bioscience) (*n* = 6 in each group); **I** The ratio of NAD^+^ to NADH was evaluated by NAD + /NADH Assay Kit with WST-8 (*n* = 3 in each group); **J** The expression of Sirt1 in HUVECs was measured by Western blot (*n* = 6 in each group). **K** Representative images of immunofluorescence co-staining CD31 and Ndufa3 in lung tissues; Scale bar: 40 µm**; L** Western blot analysis showed the expression of Sirt1 in lung tissues (*n* = 6 in each group). Each bar showed means ± SEM. Unpaired *t*-test was used for the comparison between two groups. Comparison among three or more groups was analyzed by one-way ANOVA. *p < 0.05, **p < 0.01 versus control group. ^#^p < 0.05, ^##^p < 0.01 versus S100a8/a9-treated or sepsis group. ▲p < 0.05, ▲▲p < 0.01 versus NRF1 overexpression group or NMN-treated sepsis group.

Since mitochondrial complex I is responsible for the first step of electron transport during OXPHOS, we next analyzed the effects of S100a8/a9 on mitochondrial function, and endothelial cells overexpressing NRF1 were constructed to explore its protective effects on mitochondria. First, the RT‒qPCR and Western blot results indicated the successful generation of NRF1-overexpressing cells (Fig. 4A, B). Immunofluorescence analysis confirmed that overexpression of NRF1 reversed S100a8/a9-induced Ndufa3 expression downregulation (Fig. 4C). Moreover, paquinimod treatment upregulated Ndufa3 expression in endothelial cells from the CLP group (Fig. 4K). Next, we evaluated changes in mitochondrial structure and function. We used TRME, a fluorescent probe that can penetrate the cell membrane, to detect changes in the mitochondrial membrane potential (ΔΨ). The fluorescence images suggested that NRF1 overexpression mitigated the S100a8/a9-induced loss of mitochondrial membrane potential (Fig. 4D). Additionally, we determined the oxygen consumption rate (OCR) to assess mitochondrial function. The results showed that S100a8/a9 mainly inhibited the maximal respiration capacity but had no noticeable effect on the basal respiration capacity or ATP-linked respiration capacity. However, the overexpression of NRF1 was not sufficient to restore maximal respiration capacity significantly (Fig. 4E–H).

Although the five mitochondrial complexes are all involved in OXPHOS, their functions vary. For example, mitochondrial complex I is a master regulator of NAD^+^ and NADH intracellular levels, because it can oxidize NADH to NAD^+^ [32]. Therefore, we determined the ratio of NAD^+^ to NADH in endothelial cells to evaluate complex I activity. We found that S100a8/a9 inhibited the activity of complex I, but NRF1-overexpressing endothelial cells resisted this effect (Fig. 4I). Based on our previous study, Sirt1, an NAD^+^-dependent deacetylase, can modulate mitochondrial homeostasis [33]. NRF1-overexpressing endothelial cells reversed the S100a8/a9-induced downregulation of Sirt1 expression (Fig. 4J). Moreover, the administration of β-nicotinamide mononucleotide (β-NM, NMN), a key NAD^+^ intermediate, significantly upregulated Sirt1 expression in lung tissues from the CLP group. However, when septic mice were treated simultaneously with NMN and the Sirt1 inhibitor Selisistat (EX-527), the expression of Sirt1 decreased (Fig. 4L). Therefore, in addition to inhibiting Sirt1 activity, using EX-527 could partially suppress NMN-induced upregulation of Sirt1 expression.

In general, S100a8/a9 inhibits mitochondrial complex I activity, which induces mitochondrial dysfunction and NAD^+^-dependent Sirt1 expression suppression.

### S100a8/a9 disturbs mitochondrial homeostasis via excessive mitochondrial fission and blocked mitophagy

**Fig. 5:**
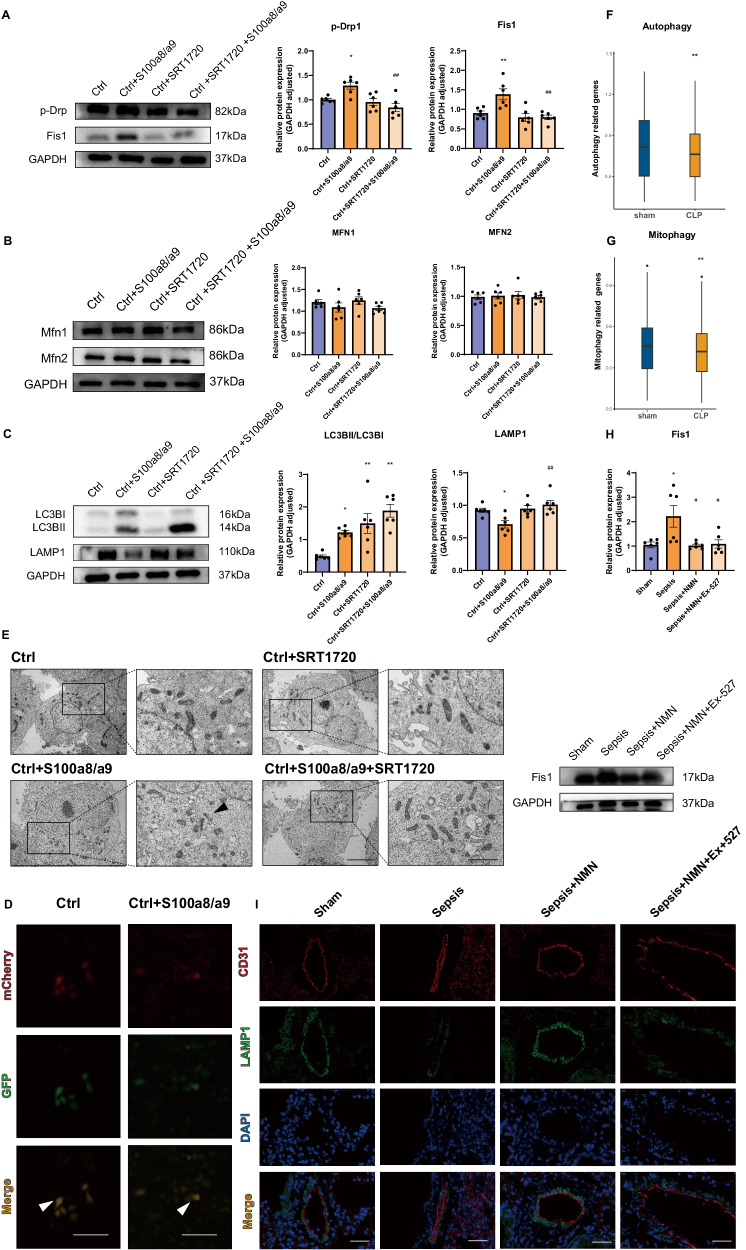
S100a8/a9 induces excessive mitochondrial fission and impaired mitophagy, both of which disturb mitochondrial homeostasis. **A**–**C** The expression levels of mitochondrial fission, fusion and mitophagy-related proteins were evaluated by Western blot analysis (*n* = 6 in each group); **D** Representative fluorescent images of endothelial cells transfected with mCherry-EGFP-LC3B; Scale bar: 200 µm; **E** Images from TEM showed impaired mitochondria in cytoplasm; Scale bar: 2 µm, 5 µm; **F**, **G** Autophagy- and mitophagy-related genes in endothelial cells from lung tissues were evaluated by GSVA enrichment analysis; **H** Fission-related protein expression level was assessed by Western blot (*n* = 6 in each group); **I** Representative images of immunofluorescence co-staining CD31 and LAMP1 in lung tissues; Scale bar: 40 µm. Each bar showed means ± SEM. Unpaired *t*-test was used for the comparison between two groups. Comparison among three or more groups was analyzed by one-way ANOVA. *p < 0.05, **p < 0.01 versus control group. ^#^p < 0.05, ^##^p < 0.01 versus S100a8/a9-treared or sepsis group. ▲p < 0.05, ▲▲p < 0.01 versus NMN-treated sepsis group.

Mitochondrial function particularly relies on the dynamic balance among mitochondrial biogenesis, fission and fusion, and the degradation of impaired mitochondria through mitophagy [34]. Previous studies have confirmed the vital role of Sirt1 expression in maintaining mitochondrial homeostasis. Western blot analysis demonstrated that S100a8/a9 led to upregulated expression of fission-related proteins, such as phospho-Drp (p-Drp) and Fis1. Treatment with the Sirt1 activator SRT1720 reversed this effect (Fig. 5A). However, S100a8/a9 has no apparent effect on fusion-related proteins, such as Mfn1 and Mfn2, which confirms the imbalance between fission and fusion (Fig. 5B). Additionally, S100a8/a9 was shown to increase the expression of LC3B, a marker of autophagosomes. However, the complete process of autophagy consists of autophagosome formation and the fusion of autophagosomes and lysosomes [35]. The expression of the lysosome marker LAMP1 was downregulated by S100a8/a9 administration. The use of a Sirt1 activator inhibited its downregulation (Fig. 5C). The above results confirmed that autophagic flux was blocked by S100a8/a9 and that LC3B might passively accumulate. mCherry-GFP-LC3B fluorescence analysis confirmed the blockade of autophagic flux (Fig. 5D). Impaired mitophagy might result in the accumulation of damaged mitochondria. Transmission electron microscopy (TEM) analysis revealed that using Sirt1 activator decreased impaired mitochondria in the cytoplasm after S100a8/a9 stimulation (Fig. 5E).

Additionally, based on 504 autophagy-related genes [36] and 29 mitophagy-related genes [37] (Supplementary Tables 8–9), GSVA results confirmed that the activities of autophagy- and mitophagy-related pathways were significantly downregulated in endothelial cells from the CLP group (Fig. 5F–G). Moreover, the Western blot and immunofluorescence results also revealed excessive mitochondrial fission and impaired mitophagy in septic mice, and NMN administration restored mitochondrial homeostasis. However, the simultaneous treatment with NMN and Sirt1 inhibitor suppressed this protective effect on mitophagy (Fig. 5H, I). It indicated that NMN supplementation maintained mitochondrial homeostasis in a Sirt1-dependent manner, since Ex-527 could inhibit Sirt1 activity and partially suppress NMN-induced upregulation of Sirt1 expression (Fig. 4L).

Generally, S100a8/a9 induced excessive fission and impaired mitophagy in a Sirt1-dependent manner.

### S100a8/a9 induces increased mtDNA release, which initiates ZBP1-mediated PANoptosis

**Fig. 6:**
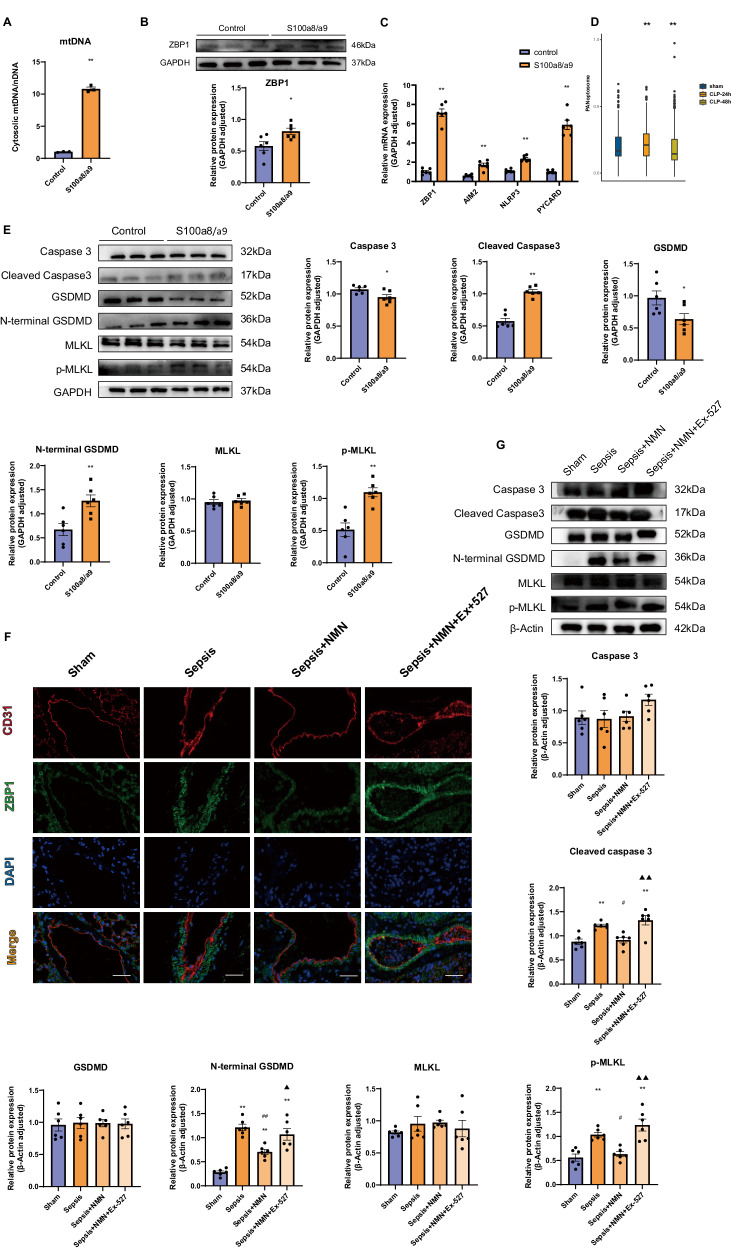
S100a8/a9 induces ZBP1-mediated PANoptosis through the release of mtDNA from damaged mitochondria. **A** Cytosolic mtDNA/nDNA was detected by RT-qPCR (*n* = 3 in each group); **B** The expression level of ZBP1 protein was evaluated by Western blot (*n* = 6 in each group); **C** The expression levels of PANoptosome components were measured by RT-qPCR (*n* = 6 in each group); **D** PANoptosome formation-related genes in endothelial cells from lung tissues were assessed by GSVA enrichment analysis; **E** PANoptosis-related proteins in HUVECs treated with or without S100a8/a9 were evaluated by Western blot (*n* = 6 in each group); **F** Representative images of immunofluorescence co-staining CD31 and ZBP1 in lung tissues; Scale bar: 40 µm; **G** PANoptosis-related proteins from lung tissues were assessed by Western blot (*n* = 6 in each group). Each bar showed means ± SEM. Unpaired *t*-test was used for the comparison between two groups. Comparison among three or more groups was analyzed by one-way ANOVA. *p < 0.05, **p < 0.01 versus control group. ^#^p < 0.05, ^##^p < 0.01 versus S100a8/a9-treared or sepsis group. ▲p < 0.05, ▲▲p < 0.01 versus NMN-treated sepsis group.

According to previous studies, mtDNA released from damaged mitochondria might initiate ZBP1-induced PANoptosis, a novel type of programmed cell death consisting of apoptosis, pyroptosis and necroptosis [17]. Therefore, we first assessed the ratio of mtDNA to nuclear DNA (nDNA) in the cytoplasm of endothelial cells. We confirmed that S100a8/a9 stimulation considerably increased mtDNA in the cytoplasm (Fig. 6A). Moreover, S100a8/a9 also upregulated ZBP1 protein expression (Fig. 6B). According to RT-qPCR analysis, several PANoptosome components such as ZBP1, AIM2, NLRP3 and PYCARD, increased significantly in S100a8/a9-treated endothelial cells. And PANoptosome-related gene expression was significantly enriched in endothelial cells from the CLP-24h group [38] (Fig. 6D, Supplementary Table 10). Next, the expression of marker proteins correlated with apoptosis, pyroptosis and necroptosis were evaluated by Western blotting. The results confirmed that activated proteins, such as cleaved caspase-3, N-terminal GSDMD and phospho-MLKL (p-MLKL), showed upregulated expression following S100a8/a9 stimulation (Fig. 6E). In animal experiments, immunofluorescence and Western blot analyses demonstrated that the administration of NAD^+^ reversed the upregulation of PANoptosis-related protein expression in sepsis, while administration of the Sirt1 inhibitor decreased these protective effects (Fig. 6F–G). These findings indicate that S100a8/a9 administration activates ZBP1-mediated PANoptosis via increased mtDNA release in the cytoplasm.

### S100A8/A9^hi^ neutrophils may increase the number of circulating endothelial cells and strongly positively correlate with poor prognosis in sepsis patients

**Fig. 7:**
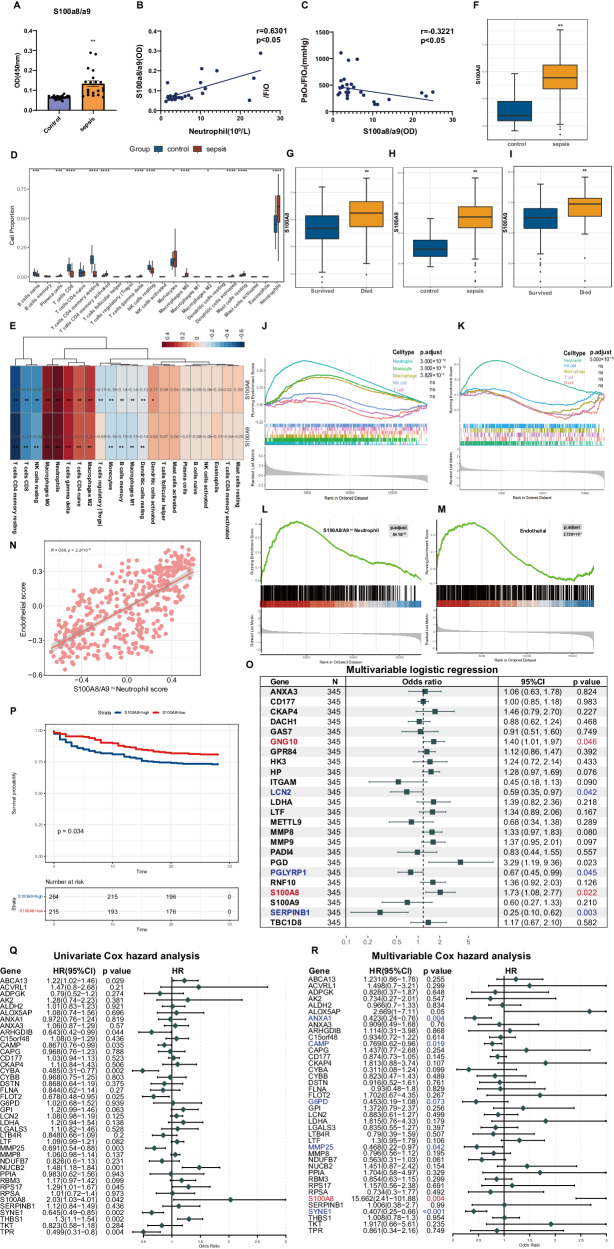
S100a8/a9^hi^ neutrophils may increase circulating endothelial cells, and show a positive correlation with poor prognosis of sepsis patients. **A** The concentration of S100a8/a9 in plasma from healthy people and sepsis patients was detected by ELISA analysis (*n* = 20 in each group); **B** The correlation curve between Neutrophil and S100a8/a9 (*n* = 40); **C** The correlation curve between S100a8/a9 and PaO_2_/FiO_2_ (*n* = 39); **D** The proportions of 22 types of immune cells in plasma were evaluated by cibersort analysis; **E** Heatmap showed the correlation between immune cells and S100A8/A9 with Spearman’s correlation analysis; **F**, **H** The expression levels of S100A8 and S100A9 were evaluated between control and sepsis groups on the box plot; **G**, **I** The expression levels of S100A8 and S100A9 were evaluated in survived and died groups on the box plot; **J** Six immune cells-related genes, which based on scRNA-seq data, were evaluated by GSEA enrichment in control and sepsis groups (Theoretically, macrophages do not exist in the circulation. Owing to the similarity in genes between macrophage and monocyte, the results of enrichment analysis indicated the existence of macrophages in the plasma); **K** Five immune cells-related genes, which based on scRNA-seq data, were evaluated by GSEA enrichment in survived and died groups; **L** S100A8/A9^hi^ neutrophil-related genes were evaluated by GSEA enrichment in survived and died groups; **M** Endothelial cell-related genes were evaluated by GSEA enrichment in survived and died groups; **N** The correlation curve between S100A8/A9^hi^ neutrophil score and endothelial cell score; **O**. To identify the risk factors for high SOFA score, a multivariate logistic regression was performed; **P** The survival curve was used to compare 28-day mortality in S100A8^high^ and S100A8^low^ groups; **Q**, **R** To identify the risk factors for 28-day mortality, univariate cox regression and multivariate cox regression were performed. Wilcoxon rank sum test and unpaired *t*-test were used for the comparison between two groups. *p < 0.05, **p < 0.01 versus control group.

Patients with sepsis exhibit increased plasma levels of S100a8/a9, accompanied by an increased number of neutrophils (Fig. 7A, B, Supplementary Table 1). Moreover, S100a8/a9 levels present a negative correlation with PaO_2_/FiO_2_, which indicates the severity of disease (Fig. 7C). On the basis of publicly available bulk RNA-seq data from 44 healthy people and 348 sepsis patients, we demonstrated that the percentage of neutrophils showed the most noticeable increase in plasma from sepsis patients by cibersort analysis (Fig. 7D, Supplementary Fig. 3A). Moreover, the expressions of S100A8 and S100A9 were positively correlated with neutrophil score (Fig. 7E, Supplementary Fig. 3B–C). Elevated expression levels of S100A8 and S100A9 were found in sepsis patients, and they were negatively associated with survival rate (Fig. 7F–I). According to the identification of marker genes in six types of immune cells via scRNA-seq (Supplementary Table. 4), we found that the expression of genes related to three types of immune cells (neutrophils, monocytes and macrophages) increased significantly in the sepsis group (Fig. 7J). However, only neutrophil-related genes showed an apparent upward trend in expression in non-surviving patients, indicating that neutrophils are closely linked with survival in sepsis patients (Fig. 7K). Moreover, the GSEA enrichment results suggested that S100A8/A9^hi^ neutrophils and NET-related gene^+^ neutrophils might accumulate in the plasma of patients who did not survive (Fig. 7L, Supplementary Fig. 3D, Supplementary Table 5, 11). Elevated circulating endothelial cell (CEC) counts might suggest vascular injury and severe disease. Previous clinical trials have shown that the CEC count considerably elevates in severe sepsis or ARDS patients [39, 40]. Therefore, endothelial cell-related genes derived from the scRNA-seq data were used for further analysis (Supplementary Table 12). We confirmed that an elevated CEC count was associated with a high mortality rate (Fig. 7M). Moreover, correlation analysis revealed a distinct positive correlation between the S100A8/A9^hi^ neutrophil score and the endothelial cell score (Fig. 7N).

Based on the median SOFA scores of all sepsis patients, they were classified into a “low SOFA score” group and a “high SOFA score” group. Then, we performed univariate logistic regression analysis of the S100A8/A9^hi^ neutrophil-related genes and selected genes with p values less than 0.001 for multivariate logistic regression (Supplementary Table 13). A forest plot indicated that S100A8 and GNG10 expressions were independent risk factors (Fig. 7O). Additionally, based on additional bulk RNA-seq data from 760 sepsis patients, people in the S100A8-high group had a lower 28-day survival probability (Fig. 7P). Moreover, we selected 40 genes that were highly expressed in S100A8/A9^hi^ neutrophils from the bulk RNA-seq data gene expression matrix. According to univariate and multivariate Cox regressions, S100A8 was confirmed to be an independent risk factor for 28-day mortality (Fig. 7Q, R). Notably, S100A9 was not included in the gene list of this dataset, which might have been caused by technological limitations at that time.

Overall, S100A8/A9^hi^ neutrophils are likely to increase the number of circulating endothelial cells, and S100A8 gene might be an independent risk factor for poor prognosis in sepsis patients.

## Discussion

**Fig. 8:**
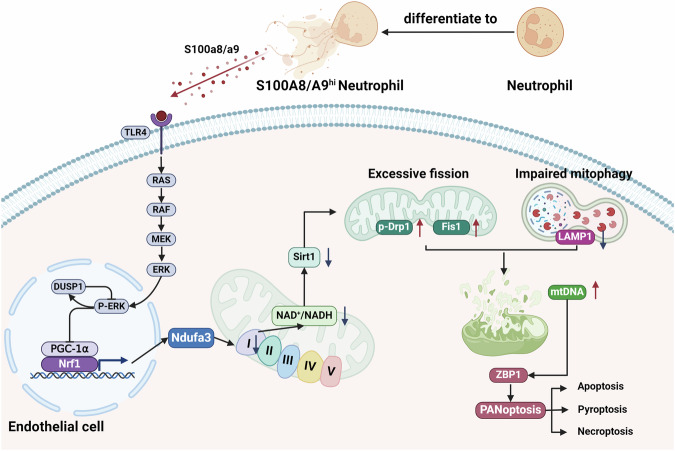
The mechanisms of S100A8/A9^hi^ neutrophils-induced PANoptosis in endothelial cells. S100a8/a9 released by S100A8/A9^hi^ neutrophils induces the negative feedback loop of Erk signaling pathway in endothelial cells, which further suppresses PGC-1α/Nrf1 expression in the nucleus. And S100a8/a9 mainly downregulates Ndufa3 expression in mitochondrial complex I via Nrf1 suppression. Moreover, mitochondrial complex I deficiency results in the decline of NAD^+^/NADH ratio and NAD^+^-dependent Sirt1 inhibition. It further causes mitochondrial disorders, including excessive fission and blocked mitophagy. Finally, mtDNA released from damaged mitochondria induces ZBP1-mediated PANoptosis.

In the present study, we demonstrated that S100a8/a9 could downregulate Nrf1-mediated Ndufa3 expression in mitochondrial complex I in endothelial cells. Complex I deficiency inhibited NAD^+^-dependent Sirt1 expression, which induced mitochondrial disorders (overactivated fission and mitophagy blockade). mtDNA released from damaged mitochondria ultimately activated ZBP1-mediated PANoptosis (Fig. 8). First, the scRNA-seq data confirmed that S100A8/A9^hi^ neutrophils were specifically present in the lung tissues of septic mice and that they strongly interacted with endothelial cells. Furthermore, high expression levels of S100a8/a9 contributed to negative feedback in the Erk signaling pathway, which suppressed Nrf1 expression in the nucleus. According to Pearson’s correlation analysis, NRF1 expression was positively linked with NDUFA3 expression in endothelial cells. Then, we generated NRF1-overexpressing HUVECs for validation. Since mitochondrial complex I deficiency decreased the NAD^+^/NADH ratio, we detected NAD^+^-dependent Sirt1 expression downregulation in vitro and in vivo. Finally, the results of animal experiments confirmed that NAD^+^ supplementation could ameliorate mitochondrial disorders and alleviate mtDNA-induced PANoptosis. These protective effects were mediated by Sirt1 since the use of a Sirt1 inhibitor reversed the effects of NAD^+^ supplementation.

A hallmark feature of sepsis is endothelial dysfunction. Damage to the endothelial barrier disrupts microcirculation and ultimately induces end-organ dysfunction [41]. S100a8/a9 released by neutrophils has been reported to participate in various inflammatory diseases [42]. According to previous studies, low concentrations of S100a8/a9 can induce inflammatory angiogenesis [43], while high expression levels of S100a8/a9 can induce endothelial cell apoptosis [9]. Based on our previous studies, NET-induced metabolic reprogramming results in cell death and exacerbates acute lung injury [11, 19]. According to the scRNA-seq data, the majority of NET-related gene^+^ neutrophils were S100A8/A9^hi^ neutrophils, and S100a8/a9 released by neutrophils might also contribute to endothelial cell death via metabolic alteration. Other studies have also confirmed that S100a8/a9 signaling induces mitochondrial disorders and cardiomyocyte death during ischemia/reperfusion injury [12].

The core components of the electron-transport chain (ETC) consist of five mitochondrial complexes, and suppression of any complex will result in mitochondrial dysfunction [44]. Among the complexes, mitochondrial complex I plays a critical role in maintaining the NAD^+^/NADH ratio and proton-motive force [45]. Therefore, mitochondrial complex I deficiency induces a decrease in the NAD^+^/NADH ratio, which may suppress NAD^+^-dependent Sirt1 expression [46]. Sirt1 is a deacetylase that belongs to the Sirtuin family, and it regulates inflammation, aging, mitochondrial biogenesis and endothelial function [47–49]. A dynamic equilibrium among fusion, fission and degradation of damaged mitochondria, called mitophagy, is critical for maintaining mitochondrial homeostasis [50, 51]. According to previous studies, the fission-related protein Drp1 was shown to be activated through acetylation. This result indicated that a decreased expression level of Sirt1 might induce excessive mitochondrial fission [52]. Moreover, we confirmed that impaired autophagic flux results from Sirt1 expression suppression during acute lung injury [33]. Overall, the downregulation of NAD^+^-dependent Sirt1 expression might disrupt mitochondrial homeostasis and cause the accumulation of damaged mitochondria in the cytoplasm.

Since ZBP1 is involved in host defense by sensing viral nucleic acids [53], PANoptosis driven by ZBP1 triggers cell death and eliminates infected cells [54]. However, the mechanisms of ZBP1 activation without viral infection remain poorly understood. In recent years, mtDNA released by impaired mitochondria has been considered as a danger-associated molecular pattern (DAMP) and may act as a potent agonist of the innate immune system. A previous study suggested that mtDNA might be sensed by ZBP1 and promote cardiotoxicity [55]. Our present study confirmed that the presence of mtDNA resulted in ZBP1-mediated PANoptosis in endothelial cells during sepsis.

There are many advantages to our research. First, comprehensive analyses of the scRNA-seq and bulk-seq data revealed that S100A8/A9^hi^ neutrophils induced mitochondrial dysfunction in endothelial cells and that S100A8 might be an independent risk factor for poor prognosis in sepsis patients. Second, based on our previous studies, inflammatory mediators released by neutrophils might inhibit mitochondrial complexes. In this study, we further confirmed that complex I was inhibited the most significantly by S100a8/a9, and NDUFA3 expression in mitochondrial complex I was closely linked with NRF1 expression via correlation analysis between NRF1 and 37 mitochondrial complex I-related genes. Finally, treatment with an NAD^+^ intermediate and a Sirt1 inhibitor showed that mitochondrial complex I deficiency contributed to mitochondrial disorders mediated by NAD^+^-dependent Sirt1 expression. Undeniably, this study has limitations. First, during sepsis progression, pathogen-associated molecular patterns (PAMPs) and DAMPs cooperatively induce cell death [56]. Therefore, the in vivo concentration of S100a8/a9 might not be as high as the concentration used in cell experiments. Second, the mechanism by which the balance of mitochondrial fission/fusion is regulated by Sirt1, such as the specific lysine acetylation sites in fission-related proteins, needs further exploration. Third, although several studies have proven that Nrf1 can regulate the transcription of mitochondrial complexes-related genes directly or indirectly [12, 30, 31], the specific mechanism by which Nrf1 regulates NDUFA3 needs further research.

In conclusion, S100a8/a9, which is mainly released by the S100A8/A9^hi^ neutrophil subcluster, can induce mitochondrial complex I deficiency. It further disrupts mitochondrial homeostasis through NAD^+^-dependent Sirt1 expression downregulation. mtDNA released from damaged mitochondria ultimately induces ZBP1-driven PANoptosis in endothelial cells.

## Methods

### Ethics statement

The research received approval from the Ethics Committee of Zhongshan Hospital, Fudan University, and adhered to the principles of the Declaration of Helsinki (Protocol license number: B2023-409). We acquired informed consent from patients or their relatives. Mouse experiments were conducted based on the Regulations for the Administration of Affairs Concerning Experimental Animals and guidelines set by animal review committee of Zhongshan Hospital, Fudan University (Protocol license number: 2023-613).

### Human subjects

The study included patients in intensive care unit (ICU) between January 2023 and December 2023. Diagnosis of sepsis was based on the Third International Consensus Definition for sepsis [57]. Exclusion criteria included: a history of cardiopulmonary arrest before being admitted to ICU; history of connective tissue diseases such as systemic lupus erythematosus (SLE), vascular embolism and pregnancy.

### Cecal ligation and puncture (CLP) mouse model

Eight- to ten-week-old male C57BL/6 mice were used for experiment. Following random grouping, the cecal ligation and puncture (CLP) mice model was established using the procedures described in previous studies [58]. In brief, after intraperitoneal anesthesia with 1% pentobarbital sodium (1 mg/kg), the abdominal cavity was opened. The cecum was carefully separated, ligated using 5-0 suture, and punctured with a 20-gauge needle. Next, we extruded a small quantity of feces from cecum and repositioned it before closing abdominal cavity. Each animal received 0.5 ml/10 g of normal saline for rehydration. The sham group received the same surgery without CLP. When mice required treatments, the following drugs were injected intraperitoneally: Paquinimod (10 mg/kg, MCE, Shanghai, China), β-nicotinamide mononucleotide (NMN, 500 mg/kg, MCE, Shanghai, China), Selisistat (EX-527, 5 mg/kg, MCE, Shanghai, China) [46, 59].

### Murine sepsis score (MSS)

Seven clinical variables in MSS, including appearance, level of consciousness, activity, response to stimulus, eyes, respiration rate and respiration quality were used to assess the severity of sepsis. Each indicator has a score of 0-4, with a full score of 28. Higher scores mean more severe injury [60, 61].

### Flow cytometry

Blood samples from mice were treated with red blood cell lysis buffer [46, 59] (Thermo Fisher). After centrifugation, the supernatant was discarded. The prepared cell suspensions were stained in PBS with the following antibodies: APC anti-mouse/human CD11b (BioLegend, 101211), FITC anti-mouse Ly-6G (BioLegend, 127605), PE anti-S100A9 (Cell Signaling Technology, #93941). Flow cytometry was performed using a BD FACS Aria III flow cytometer according to manufacturer protocol.

### Histopathological analysis

Mice were sacrificed for several organs, such as lung, liver, kidney, spleen and intestine, and these tissues were fixed with 4% paraformaldehyde at room temperature for 24 h. Paraffin-embedded tissue sections were stained with hematoxylin and eosin (H&E). According to previous descriptions, the severity of acute lung injury was evaluated through a semiquantitative histology scoring method [62]. Specifically, the score of lung injury was based on these indicators: leukocyte infiltration, alveolar edema, haemorrhage and the thickness of alveolar septa. Two pathologists who blinded to the results graded each indicator from 0 to 3 (0 = normal; 1 = mild; 2 = moderate; 3 = severe), and finally calculated the total lung injury score. Furthermore, paraffin-embedded lung tissue sections were also stained with Masson dye to identify the degree of fibrosis within lung tissues.

### TUNEL staining

Paraffin-embedded lung tissue sections were stained with TUNEL to detect cell apoptosis according to manufacturer protocol.

### Lung wet-to-dry ratio

We harvested the left lung of mice and obtained its wet weight after drying the surface water. Subsequently, the tissue was dried at 70 °C for 48 h, and the dry weight was acquired. The wet/dry ratio was calculated by dividing the wet weight with dry weight. High wet-to-dry ratio means more severe lung edema.

### Semi-quantification of inflammatory mediators

The levels of IL-1β, IL-6 and TNF-α in mouse serum were evaluated by Mouse IL-1β ELISA kit (mIC50300-1, mlbio, Shanghai, China), Mouse IL-6 ELISA kit (IC50325-1, mlbio, Shanghai, China), Mouse TNF-α ELISA kit (mIC50536-1, mlbio, Shanghai, China). Moreover, the levels of S100a8/a9 in mouse serum and human plasma were detected by Mouse S100a8/a9 ELISA kit (ml037985, mlbio, Shanghai, China) and Human S100a8/a9 ELISA kit (ml038517, mlbio, Shanghai, China).

### Cell culture and treatments

We obtained Human Umbilical Vein Endothelial Cells (HUVECs) from American Type Culture Collection (ATCC; Manassas, USA) and cultured them in DMEM (Gibco) containing 10% fetal bovine serum (Gibco) and penicillin/streptomycin (Gibco) at 37 °C, 5% CO_2_ incubator. S100a8&a9 heterodimer protein was purchased from SinoBiological (Beijing, China). Sirt1 activator SRT1720 was purchased from MCE (5 µM, Shanghai, China) [63]. HUVECs were transfected with Lentivirus-NC or Lentivirus-NRF1 (MOI = 10, Shanghai GeneChem, China) for 72 h to overexpress NRF1, and the sequence was listed in Supplementary Table 3.

### Cell viability

A Cell-Counting Kit 8 (Dojindo Corp., Kumamoto, Japan) was used to measure relative cell viability based on manufacturer’s instructions.

### The Ad-mCherry-GFP-LC3B fluorescence microscopy assay

HUVECs were seeded in 48-well plates (5 × 10^4^ cells/well) one day before transfection. Cells were transfected with adenovirus expressing mCherry-GFP-LC3B fusion protein (MOI = 20, Beyotime, Shanghai, China) for 24 h and then treated with or without S100a8/a9. The images were taken by Olympus microscope. In the absence of autophagy, mCherry-GFP-LC3B under fluorescence microscopy showed dispersed yellow fluorescence. However, in the presence of autophagy, mCherry-GFP-LC3B aggregated on the membrane of autophagosome showing yellow spots. After the fusion between autophagosomes and lysosomes, red dots could be observed, since GFP fluorescence was quenched partially.

### Mitochondrial membrane potential assay kit with TMRE

HUVECs were seeded in 48-well plates (2 × 10^4^ cells/well) with or without S100a8/a9 stimulation for 24 h. Then these cells were incubated with TMRE staining working solution (Beyotime, Shanghai, China) at 37 °C, 5% CO_2_ incubator for 30 min. After that, the supernatant was removed and the cells were washed with medium twice. The samples were then observed under immunofluorescence microscopy. Loss of mitochondria membrane potential was shown by diminished red fluorescence, which occurred in the early stage of cell apoptosis.

### Measurement of mitochondrial oxidation

The oxygen consumption rate (OCR) of HUVECs was measured by the Agilent Seahorse XF Cell Mito Stress Test on the Seahorse XFe and XF Extracellular Flux Analyzers. HUVECs (2 × 10^4^ cells/well) were seeded in an XF96 plate and incubated in a medium containing glucose, pyruvate and glutamine. Oligomycin, FCCP and rotenone were used to evaluate OCR. Seahorse Wave software was used to assess all data.

### Transmission electron microscopy (TEM)

HUVECs were fixed in 2.5% glutaraldehyde, and then were post-fixed with 1% osmic acid for 2 h. Next, gradient dehydration was performed by the usage of graded ethanol. The sample was embedded in 812 resin, which followed by thin section staining of 2% uranyl acetate. Finally, the ultrastructural images of mitochondria were acquired by the usage of transmission electron microscope (HT7700, Hitachi).

### Cytosolic mtDNA isolation

After lysis, HUVECs were centrifuged at 700 × *g* for 10 min to remove nuclei. Then, we normalized the supernatant volume based on protein concentration. Cell lysate was further centrifuged at 10,000 × *g* for 30 min for cytosolic fraction isolation, which included mtDNA and nDNA [17]. mtDNA was assessed by RT-qPCR with gene sequences coding for human NADH dehydrogenase 1 as primers. Nuclear DNA was detected using sequences coding human b-globin as primers [23]. The primers for human NADH dehydrogenase 1 and human b-globin were listed in Supplementary Table 2.

### NAD^+^/NADH measurement

NAD^+^/NADH Assay Kit with WST-8 (Beyotime, Shanghai, China) was used for measure mitochondrial complex I activity. HUVECs were seeded in a 6-well plate (1 × 10^6^ cells/well) before they were lysed with 200 μl NAD^+^/NADH extraction solution. Then, 100 μl samples were added in centrifugal tubes and heated at 60 °C for 30 min to decompose NAD^+^. Supernatant was further mixed with working solution and the absorbance of samples was measured at 450 nm.

### Immunofluorescence

HUVECs were seeded in 48-well plates (2 × 10^4^ cells/well) with or without S100a8/a9 stimulation. 4% paraformaldehyde was used for fixation for 10 min. The cells were penetrated by using 0.1% Triton for 5 min and further blocked at room temperature for 30 min. Antibody against Ndufa3 (1:500, sc-365351, Santa Cruz Biotechnology), was used for incubation overnight at 4 °C, and Alexa Fluor® 594-conjugated goat anti-rabbit IgG (1:200, ab150080, Abcam) was used to incubation at room temperature for 1 h next day. Finally, the nuclei were stained with 4,6-diamidino-2-phenylindole (DAPI). In order to visualize the expressions of target proteins in endothelial cells from mice lung tissues, paraffin-embedded tissue sections were deparaffinized, rehydrated for antigen retrieval. The primary antibodies used in this study included anti-S100a8 (1:500, GB11421-100, Servicebio), anti-S100a9 (1:750, GB111149-100, Servicebio), anti-CD31 (1:200, GB12063-100, Servicebio), anti-Ndufa3 (1:500, sc-365351, Santa Cruz Biotechnology), anti-LAMP1 (1:500, sc-20011, Santa Cruz Biotechnology); anti-ZBP1 (1:500, 13285-1-AP, Proteintech). And the secondary antibodies used here included iF488-Tyramide (1:500, G1231-50UL, Servicebio) and iF555-Tyramide (1:500, G1233-50UL, Servicebio).

### Quantitative real-time PCR

The total RNA from cells and tissues was extracted by using TRIzol reagent (Thermo Fisher), and the quality and quantity of RNA were measured by NanoDropTM ND-1000. PrimeScript RT reagent kit (RR036A, Takara, Shinga, Japan) was used to reverse-transcribe RNA into cDNA. Then we used TB Green PCR kit (RR820A, Takara) and Bio-Rad system to perform RT-qPCR with two repetitions per well. The primer sequences were listed in Supplementary Table 2.

### Western blot

Cells and lung tissues were lysed with RIPA Buffer (Solarbio, Beijing, China), which contains proteinase inhibitor cocktails. Sodium dodecyl sulfate-polyacrylamide gel electrophoresis (SDS-PAGE) were used to separate proteins. And then proteins were transferred to polyvinylidene fluoride (PVDF) membranes. The membranes were immersed in blocking buffer and incubated with primary antibodies against S100a8/a9 (1:1000, ab288715, Abcam); phospho-MEK1/2 (1:1000, #9154 S, Cell Signaling Technology); phospho-Erk/2 (1:2000, #4370, Cell Signaling Technology); PGC-1α (1:1000, #AF5395, Affinity); Nrf1 (1:1000, 66832-1-Ig, Proteintech); Total OXPHOS Rodent WB antibody cocktail (6.0 µg/ml, ab110413, Abcam); Sirt1 (1:500, sc-74465, Santa Cruz Biotechnology); phospho-Drp (1:1000, #3455, Cell Signaling Technology); Fis (1:500, sc-376447, Santa Cruz Biotechnology); Mfn1 (1:500, sc-166644, Santa Cruz Biotechnology); Mfn2 (1:500, sc-100560, Santa Cruz Biotechnology); LC3B (1:1000, #2775, Cell Signaling Technology); LAMP1 (1:500, sc-20011, Santa Cruz Biotechnology); ZBP1 (1:500; sc-271483, Santa Cruz Biotechnology); caspase 3 (1:500, sc-56053, Santa Cruz Biotechnology); cleaved caspase 3 (1:500, ab2302, Abcam); GSDMD (1:1000, ab219800, Abcam); N-terminal GSDMD (1:1000, ab215203, Abcam); MLKL (1:5000, 66675-1-Ig, Proteintech); phospho-MLKL (1:1000, ab196436, Abcam); phospho-MLKL (1:1000; ab187091, Abcam); GAPDH (1:1000, GB15004-100, Servicebio); β-Actin (1:1000, GB15003-100, Servicebio).

### Bulk RNA-seq and scRNA-seq data

Bulk RNA-seq data from pneumonia-induced sepsis patients (GSE65682) and sepsis patients in ICU (GSE185263) were used to reanalyzed. scRNA-seq data of lung tissues from sham and CLP mice (GSE 207651) were selected for further analysis.

### ScRNA-seq data pre-processing

We transferred merged matrix into the R statistical environment for further analysis through Seurat package (v. 4.0.4). Cells expressing <200 or >2500 genes, >5% mitochondrial reads were removed. “NormalizeData” function was performed to normalize the gene expression matrix, and 2000 highly variable genes (HVGs) were identified using “FindVariableFeatures” function. Then, the data were integrated among different samples based on identified anchor points using “FindIntegrationAnchors” function. Finally, we used “FindNeighbors” and FindCluster” functions to cluster and identify cells. And cell clusters were visualized by “RunTSNE” and “RunUMAP” functions.

### Cluster marker identification and cell annotation

We identified the differentially expressed genes (DEGs) of each cluster using “FindAllMarkers” function, and the clusters were annotated based on classic marker genes [64].

### Pseudotime analysis

We constructed differentiation trajectory using “Monocle 2” with DDRTree and the default parameter.

### Pathway and functional enrichment analysis

Gene Ontology (GO) analysis was performed by “clusterProfiler” R package. Gene set enrichment analysis (GSEA) was performed by GSEA software. And gene set variation analysis (GSVA) scores were calculated through “gsva” function. We showed gene lists in supplementary tables.

### Cell-cell communication analysis

“CellChat” package was used to evaluate the interactions between cells.

### Correlation analysis

The correlation of genes in every endothelial subcluster was analyzed by “corrplot” R package.

### The analysis of immune cells proportion

22 immune cells proportions were calculated by the CIBERSORT algorithm.

### Survival analysis

“Survival” and “Survminer” R packages were used for Survival analysis. Sepsis patients were classified as “S100A8^high^” and “S100A8^low^” groups using “surv_cutpoint” function.

### Logistic regression analysis

We took an intersection of marker genes of S100A8/A9^hi^ neutrophils and gene lists from peripheral blood leukocytes of sepsis patients, and then conducted univariate logistic regression analysis. 24 genes (p < 0.001) were selected for multivariate logistic regression analysis to seek independent risk factors of high SOFA scores (>median of SOFA scores).

### Cox regression analysis

We selected significantly upregulated marker genes in S100A8/A9^hi^ neutrophils according to scRNA-seq data. Then, we took an intersection of marker genes of S100A8/A9^hi^ neutrophils and gene lists from peripheral blood leukocytes of sepsis patients. The top 40 genes ordered by log_2_FC were chosen for univariate and multivariate cox regression analyses.

### Statistical analysis

We carried out all statistical analyses using v.4.0.0 R and GraphPad Prism 8 software. Experimental data were expressed as means ± standard error of the means (SEM). Unpaired *t*-test and Wilcoxon rank sum test were used for the comparison between two groups, and one-way ANOVA was used for three or more groups. p < 0.05 was considered statistically significant (*/#/▲p < 0.05, **/##/▲▲p < 0.01).

### Supplementary information

Supplementary Figure1-3

Supplementary Table1-13

Original Data File

## Data Availability

All experimental data are available and requested to Professor Changhong Miao.
