# Supplementary material for: S100A8/A9hi neutrophils induce mitochondrial dysfunction and PANoptosis in endothelial cells via mitochondrial complex I deficiency during sepsis
Source: Cell Death Dis. 2024 Jun 28;15(6):462. doi: 10.1038/s41419-024-06849-6 (PMC11213914; doi:10.1038/s41419-024-06849-6)
Supplement: Supplementary file 1 — Supplementary Figure1-3 [file 41419_2024_6849_MOESM1_ESM.pdf]

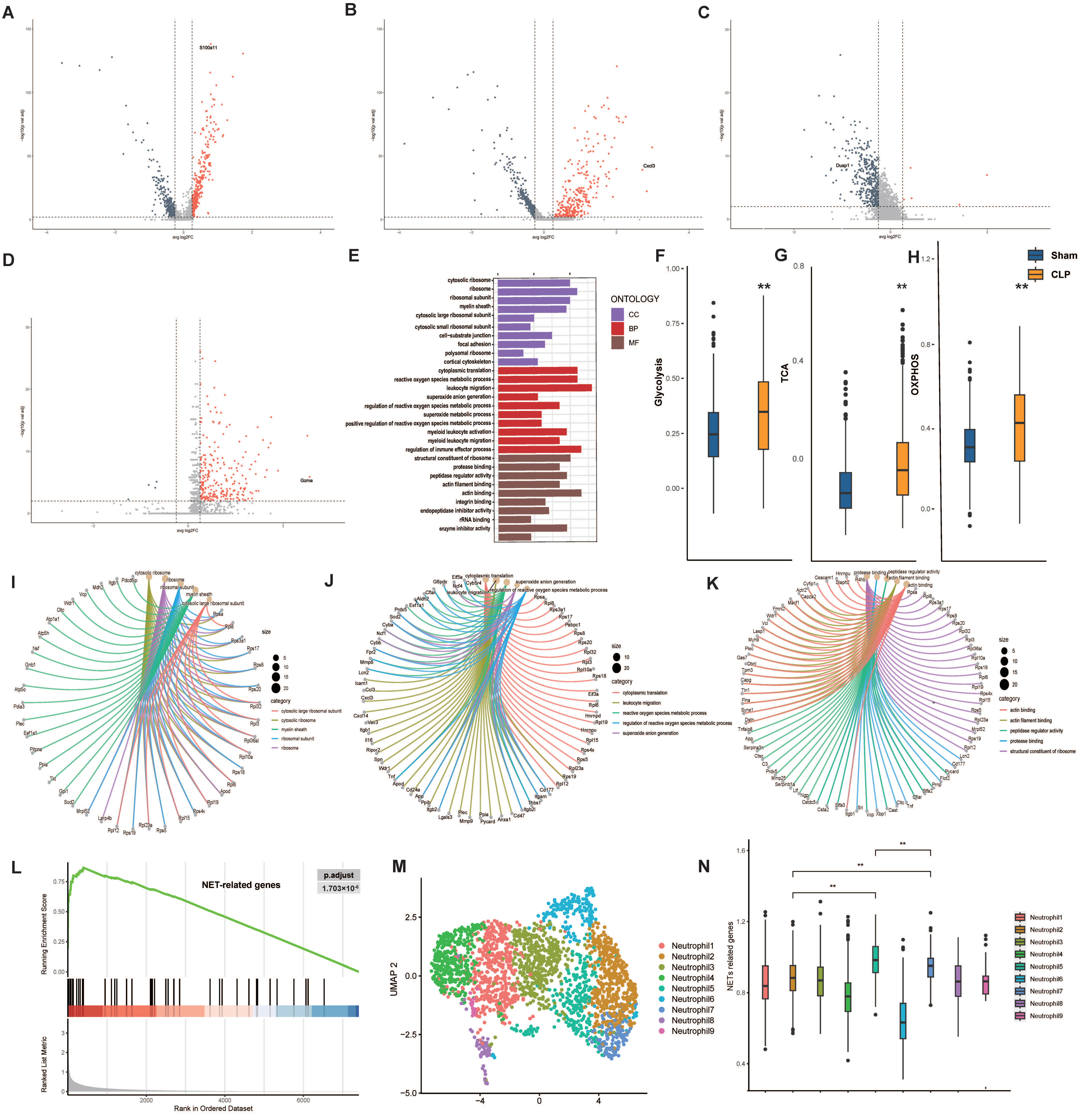

Supplementary Fig. 1

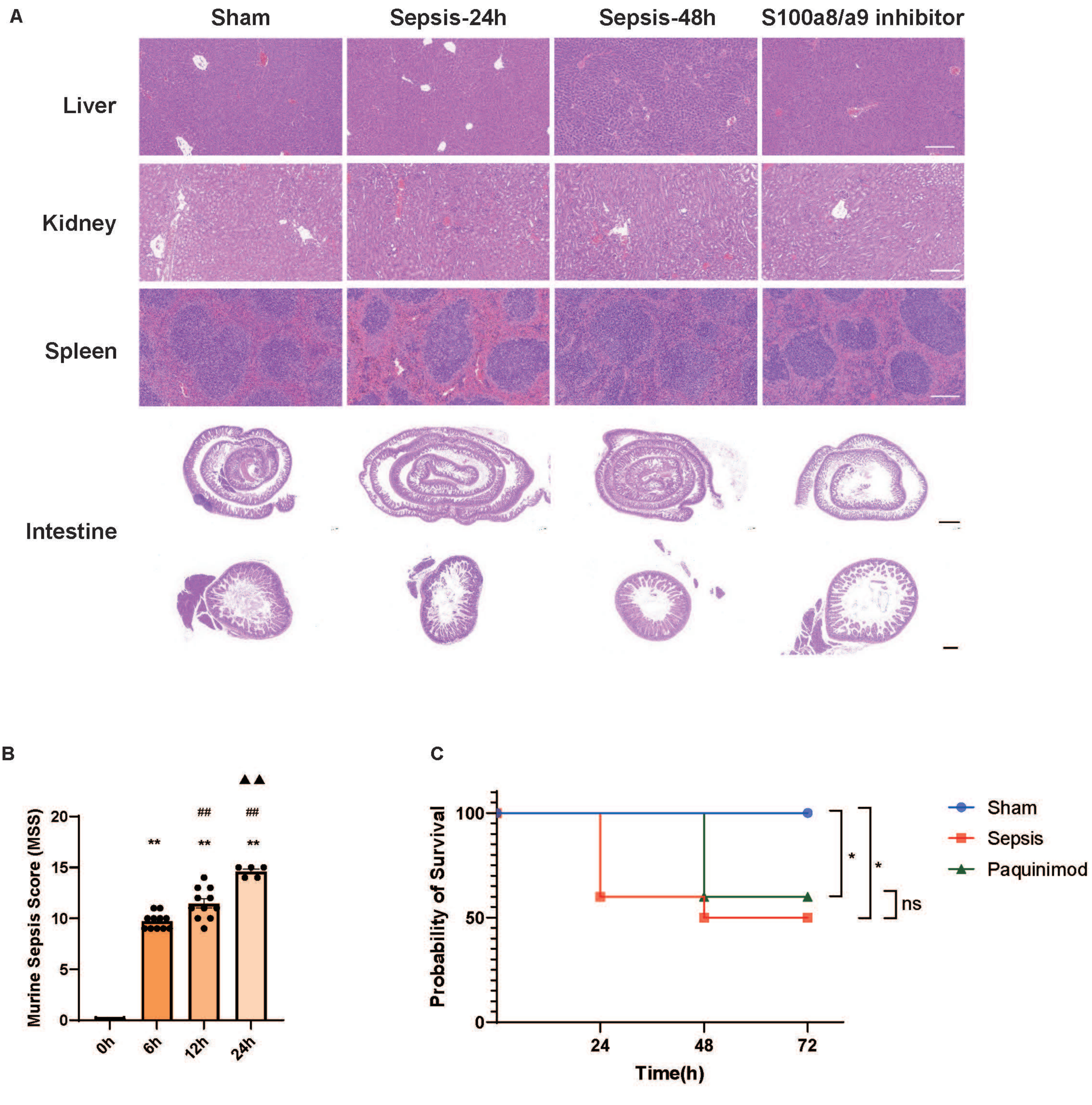

Supplementary Fig. 2

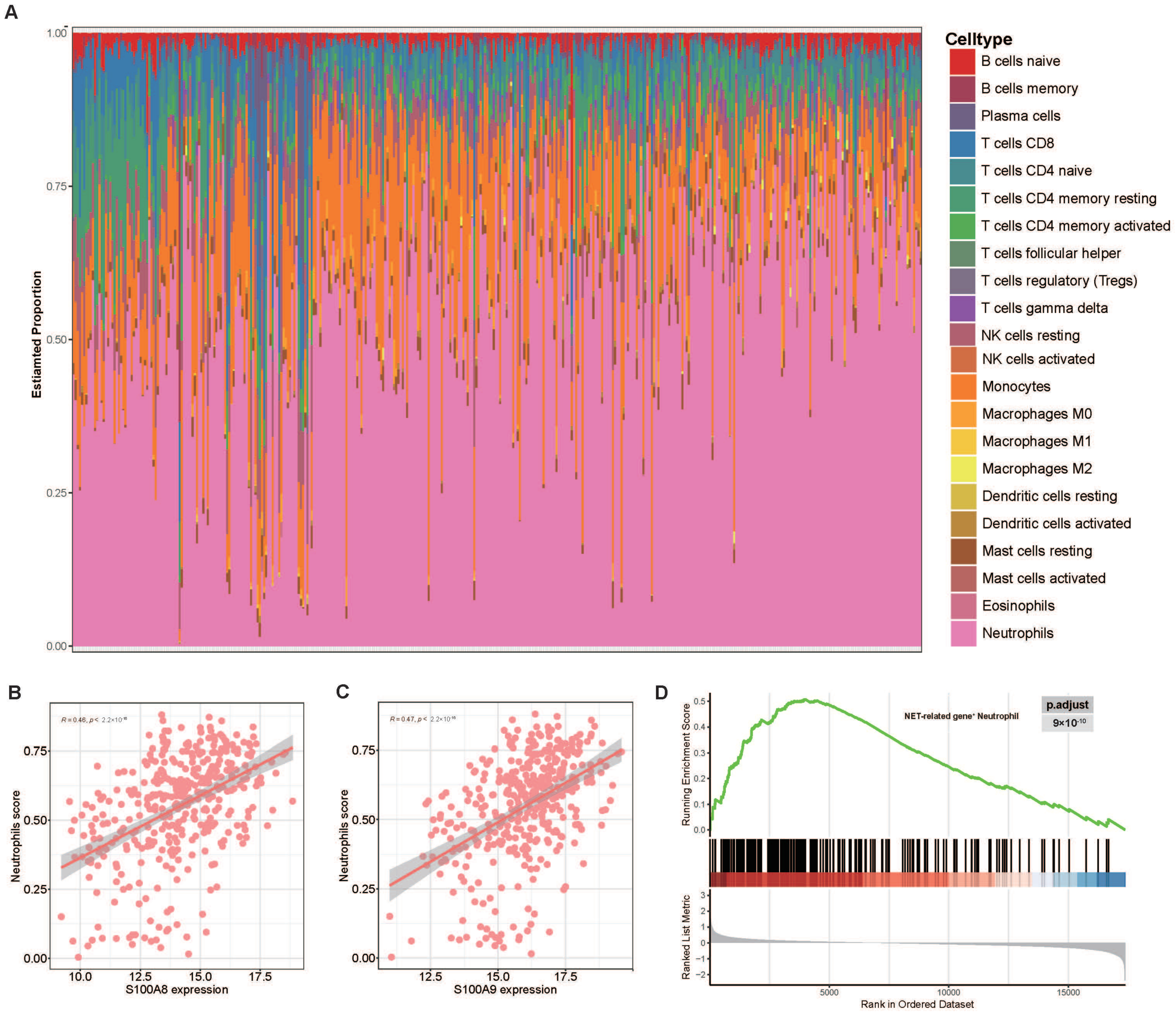

Supplementary Fig. 3

Supplementary Fig. 1 The changes of immune function and metabolism in neutrophils during sepsis. A-D. Volcano maps showed upregulated or downregulated genes in every neutrophil subcluster; **E.** GO enrichment analysis showed significantly upregulated signaling pathways in neutrophils from septic mice; **F-H.** The changes of metabolic pathways in neutrophils analyzed by GSVA enrichment; **I-K.** Cnetplot listed genes in the enriched pathways; **L.** GSEA enrichment analysis was performed according to NET-related genes; **M.** Nine neutrophil clusters were shown on the UMAP plot; **N.** Box plot showed the degree of enrichment in every neutrophil cluster. Wilcoxon rank sum test was used for the comparison between two groups. *$p<0.05$, **$p<0.01$ versus sham group.

Supplementary Fig. 2 The protective effects of S100a8/a9 inhibitor on organ damage and survival rate. A. H&E staining of several organs; Scale bar: 200μm (liver, kidney, spleen), 1000μm (intestine); 400μm (cross-sections of intestine); **B.** MSS was measured at 0h, 6h, 12h and 24h after CLP; **C.** The survival curve of three groups. Each bar showed means ± SEM. Unpaired t-test was used for the comparison between two groups. Comparison among three or more groups was analyzed by one-way ANOVA. *$p<0.05$, **$p<0.01$ versus control group. # $p<0.05$, # #$p<0.01$ versus the time point at 6h post-CLP. ▲$p<0.05$, ▲▲$p<0.01$ versus the time point at 12h post-CLP.

Supplementary Fig. 3 The noticeably increased immune cells in the circulation from sepsis patients. A. The proportions of 22 immune cells in the circulation; **B.** The correlation curve between S100A8 and neutrophil score; **C.** The correlation curve between S100A9 and neutrophil score; **D.** NET-related genes were evaluated by GSEA enrichment analysis in the survived group and died group.
