# Supplementary material for: S100A8/A9hi neutrophils induce mitochondrial dysfunction and PANoptosis in endothelial cells via mitochondrial complex I deficiency during sepsis
Source: Cell Death Dis. 2024 Jun 28;15(6):462. doi: 10.1038/s41419-024-06849-6 (PMC11213914; doi:10.1038/s41419-024-06849-6)
Supplement: Supplementary file 2 — Supplementary Table1-13 [file 41419_2024_6849_MOESM2_ESM.docx]

**Supplementary Table 1. Baseline characteristics of healthy controls**

**(HC) and Sepsis patients.**

|  | **Healthy Control**  **(n = 20)** | **Sepsis**  **(n=20)** | **P value** |
| --- | --- | --- | --- |
| **Gender (n, %)** |  |  | 0.99 |
| **Male** | 15(75%) | 15 (75%) |  |
| **Female** | 5(25%) | 5 (25%) |  |
| **Ages (years)** | 55.45±15.28 | 64.35±12.73 | 0.05 |
| **BMI (kg/m2)** | 23.37±3.174 | 23.34±3.522 | 0.98 |
| **Neutrophils (109/L)** | 4.465±4.485 | 10.31±6.537 | <0.05 |
| **Monocytes (109/L)** | 0.5905±0.5364 | 0.7215±0.5589 | 0.41 |
| **Lymphocytes (109/L)** | 1.755±0.7508 | 0.885±0.6862 | <0.05 |
| **Platelets (109/L)** | 179.6±59.38 | 161±110.8 | 0.14 |
| **Hemoglobin (g/L)** | 130±22.03 | 85.15±21.58 | <0.05 |
| **Albumin (g/L)** | 41.9±5.476 | 32.75±4.011 | <0.05 |
| **PT (s)** | 13.04±3.314 | 21.57±11.69 | <0.05 |
| **APTT (s)** | 28.99±2.543 | 44.71±13.74 | <0.05 |
| **Fibrinogen (g/L)** | 234.9±37.03 | 467.8±274.8 | <0.05 |
| **D-dimer (ng/ml)** | 0.5547±1.184 | 7.803±4.465 | <0.05 |
| **PaO2/FiO2 (mmHg)** | 514.7±245.5 | 293.4±108.2 | <0.05 |

Data are expressed as n (%), mean ± SD or median (interquartile range [IQR]). BMI: body mass index.

**Supplementary Table 2. Primer sequences for RT-qPCR.**

| **Gene** | **Forward** | **Reverse** |
| --- | --- | --- |
| Human-DUSP1 | AGTACCCCACTCTACGATCAGG | GAAGCGTGATACGCACTGC |
| Human-NRF1 | AGGAACACGGAGTGACCCAA | TATGCTCGGTGTAAGTAGCCA |
| Human-NDUFA3 | GGGGCCTCGCTGTAATTCTG | GACGGGCACTGGGTAGTTG |
| Human-NADH | ATACCCATGGCCAACCTCCT | GGGCCTTTGCGTAGTTGTAT |
| Human-b-globin | GTGCACCTGACTCCTGAGGAGA | CCTTGATACCAACCTGCCCAG |
| Human-ZBP1 | AACATGCAGCTACAATTCCAGA | AGTCTCGGTTCACATCTTTTGC |
| Human-AIM2 | TGGCAAAACGTCTTCAGGAGG | AGCTTGACTTAGTGGCTTTGG |
| Human-NLRP3 | GATCTTCGCTGCGATCAACAG | CGTGCATTATCTGAACCCCAC |
| Human-PYCARD | TGGATGCTCTGTACGGGAAG | CCAGGCTGGTGTGAAACTGAA |
| Human-GAPDH | GGAGCGAGATCCCTCCAAAAT | GGCTGTTGTCATACTTCTCATGG |
| Mouse-IL-1β | GAAATGCCACCTTTTGACAGTG | TGGATGCTCTCATCAGGACAG |
| Mouse-IL-6 | CTGCAAGAGACTTCCATCCAG | AGTGGTATAGACAGGTCTGTTGG |
| Mouse-TNF-α | CAGGCGGTGCCTATGTCTC | CGATCACCCCGAAGTTCAGTAG |
| Mouse-DUSP1 | TGTTGTTGGATTGTCGCTCCT | TTGGGCACGATATGCTCCAG |
| Mouse-NRF1 | AGCACGGAGTGACCCAAAC | AGGATGTCCGAGTCATCATAAGA |
| Mouse-NDUFA3 | ATGGCCGGGAGAATCTCTG | AGGGGCTAATCATGGGCATAAT |
| Mouse-GAPDH | TGACCTCAACTACATGGTCTACA | CTTCCCATTCTCGGCCTTG |

**Supplementary Table 3. The sequences of lentivirus-NRF1:**

Insertion sequences are in blue font, vector sequences are in black font, and restriction sites are underlined.

AATTCTGGCCGTTTTTGGCTTTTTTGTTAGACGAAGCTTGGGCTGCAGGTCG ACTCTAGAGGATCCCCGGGTACCGGTCGCCACCATGGAGGAACACGGAGTG ACCCAAACCGAACATATGGCTACCATAGAAGCACATGCAGTGGCCCAGCAA GTGCAGCAGGTCCATGTGGCTACTTACACCGAGCATAGTATGCTGAGTGCT GATGAAGACTCGCCTTCTTCTCCCGAGGACACCTCTTACGATGACTCAGATA TACTCAACTCCACAGCAGCTGATGAGGTGACAGCTCATCTGGCAGCTGCAG GTCCTGTGGGAATGGCCGCTGCTGCTGCTGTGGCAACAGGAAAGAAACGGA AACGGCCTCATGTATTTGAGTCTAATCCATCTATCCGGAAGAGGCAACAAAC ACGTTTGCTTCGGAAACTTCGAGCCACGTTAGATGAATATACTACTCGTGTG GGACAGCAAGCTATTGTCCTCTGTATCTCACCCTCCAAACCTAACCCTGTCTT TAAAGTGTTTGGTGCAGCACCTTTGGAGAATGTGGTGCGTAAGTACAAGAG CATGATCCTGGAAGACCTGGAGTCTGCTCTGGCAGAACACGCCCCTGCGCC ACAGGAGGTTAACTCAGAACTGCCGCCTCTCACCATCGACGGAATTCCAGTC TCTGTGGACAAAATGACCCAGGCCCAGCTTCGGGCATTTATCCCAGAGATGC TCAAGTACTCTACAGGTCGGGGAAAACCAGGCTGGGGGAAAGAAAGCTGCA AGCCCATCTGGTGGCCTGAAGATATCCCCTGGGCAAATGTCCGGAGTGATG TCCGCACAGAAGAGCAAAAGCAGAGGGTTTCATGGACCCAGGCACTACGGA CCATAGTTAAAAACTGTTATAAACAGCATGGGCGGGAAGACCTTTTGTATGC CTTTGAAGATCAGCAAACGCAAACACAGGCCACAGCCACACATAGTATAGCT CATCTTGTACCATCACAGACTGTAGTCCAGACTTTTAGTAACCCTGATGGCA CTGTCTCACTTATCCAGGTTGGTACGGGGGCAACAGTAGCCACATTGGCTG ATGCTTCAGAATTGCCAACCACGGTCACCGTTGCCCAAGTGAATTATTCTGC CGTGGCTGATGGAGAGGTGGAACAAAATTGGGCCACGTTACAGGGAGGTG AGATGACCATCCAGACGACGCAAGCATCAGAGGCCACCCAGGCGGTGGCAT CGTTGGCAGAGGCCGCAGTGGCAGCTTCTCAGGAGATGCAGCAGGGAGCTA CAGTCACTATGGCGCTTAACAGCGAAGCTGCCGCCCATGCTGTCGCCACCCT GGCTGAGGCCACCTTACAAGGTGGGGGACAGATCGTCTTGTCTGGGGAAAC CGCAGCAGCCGTCGGAGCACTTACTGGAGTCCAAGATGCTAATGGCCTGGT CCAGATCCCTGTGAGCATGTACCAGACTGTGGTGACCAGCCTCGCCCAGGG CAACGGACCAGTGCAGGTGGCCATGGCCCCTGTGACCACCAGGATATCAGA CAGCGCAGTCACCATGGACGGCCAAGCTGTGGAGGTGGTGACATTGGAACA GGGTATGGACTACAAGGATGACGATGACAAGGATTACAAAGACGA

| **Supplementary Table. 4 The marker genes of six immune cells** | | | | | | |
| --- | --- | --- | --- | --- | --- | --- |
| **gene** | **p_val** | **avg_log2FC** | **pct.1** | **pct.2** | **p_val_adj** | **cluster** |
| S100a9 | 0 | 5.547951861 | 0.999 | 0.755 | 0 | Neutrophil |
| Retnlg | 0 | 5.516033762 | 0.964 | 0.18 | 0 | Neutrophil |
| Ngp | 0 | 5.312949825 | 0.623 | 0.154 | 0 | Neutrophil |
| S100a8 | 0 | 5.139835708 | 0.999 | 0.669 | 0 | Neutrophil |
| Lcn2 | 0 | 3.839284432 | 0.949 | 0.421 | 0 | Neutrophil |
| Wfdc21 | 0 | 3.544201229 | 0.94 | 0.196 | 0 | Neutrophil |
| Asprv1 | 0 | 3.510922036 | 0.652 | 0.03 | 0 | Neutrophil |
| Ifitm6 | 0 | 3.457871976 | 0.794 | 0.287 | 0 | Neutrophil |
| G0s2 | 0 | 3.431961091 | 0.834 | 0.065 | 0 | Neutrophil |
| Stfa2l1 | 0 | 3.409758696 | 0.607 | 0.029 | 0 | Neutrophil |
| Pglyrp1 | 0 | 3.355026491 | 0.931 | 0.195 | 0 | Neutrophil |
| Mmp9 | 0 | 3.284564184 | 0.887 | 0.042 | 0 | Neutrophil |
| Acod1 | 0 | 3.27878615 | 0.802 | 0.179 | 0 | Neutrophil |
| Hdc | 0 | 3.19455921 | 0.915 | 0.068 | 0 | Neutrophil |
| Ifitm1 | 0 | 3.113800936 | 0.868 | 0.318 | 0 | Neutrophil |
| Wfdc17 | 0 | 3.008147839 | 0.888 | 0.405 | 0 | Neutrophil |
| Lrg1 | 0 | 3.006788612 | 0.912 | 0.154 | 0 | Neutrophil |
| Slfn4 | 0 | 2.990380051 | 0.873 | 0.082 | 0 | Neutrophil |
| Anxa1 | 0 | 2.880682682 | 0.852 | 0.285 | 0 | Neutrophil |
| Cd177 | 0 | 2.719950445 | 0.579 | 0.036 | 0 | Neutrophil |
| Mmp8 | 0 | 2.644904543 | 0.759 | 0.176 | 0 | Neutrophil |
| Alox5ap | 0 | 2.50260043 | 0.966 | 0.424 | 0 | Neutrophil |
| Trem1 | 0 | 2.466219867 | 0.905 | 0.242 | 0 | Neutrophil |
| S100a11 | 0 | 2.402501 | 0.99 | 0.782 | 0 | Neutrophil |
| Cxcr2 | 0 | 2.387881612 | 0.789 | 0.044 | 0 | Neutrophil |
| Slpi | 0 | 2.349344014 | 0.918 | 0.297 | 0 | Neutrophil |
| Il1r2 | 0 | 2.34587578 | 0.948 | 0.274 | 0 | Neutrophil |
| Grina | 0 | 2.327103203 | 0.892 | 0.298 | 0 | Neutrophil |
| Csf3r | 0 | 2.324974533 | 0.7 | 0.114 | 0 | Neutrophil |
| Lmnb1 | 0 | 2.285845733 | 0.9 | 0.335 | 0 | Neutrophil |
| Clec4d | 0 | 2.214768526 | 0.855 | 0.272 | 0 | Neutrophil |
| S100a6 | 0 | 2.1769699 | 0.975 | 0.708 | 0 | Neutrophil |
| Mxd1 | 0 | 2.149935191 | 0.957 | 0.48 | 0 | Neutrophil |
| Msrb1 | 0 | 2.123107522 | 0.954 | 0.467 | 0 | Neutrophil |
| Pygl | 0 | 2.103630072 | 0.822 | 0.168 | 0 | Neutrophil |
| Il1f9 | 0 | 2.084711777 | 0.539 | 0.025 | 0 | Neutrophil |
| Cd14 | 0 | 2.053135518 | 0.954 | 0.416 | 0 | Neutrophil |
| Cxcl2 | 0 | 2.034995182 | 0.951 | 0.491 | 0 | Neutrophil |
| Hcar2 | 4.99E-303 | 2.028289835 | 0.535 | 0.076 | 1.02E-298 | Neutrophil |
| Cstdc4 | 1.55E-301 | 3.319228843 | 0.452 | 0.029 | 3.17E-297 | Neutrophil |
| Camp | 9.05E-296 | 4.43355411 | 0.457 | 0.039 | 1.85E-291 | Neutrophil |
| Ltf | 1.92E-279 | 3.480043206 | 0.425 | 0.029 | 3.93E-275 | Neutrophil |
| Isg15 | 3.72E-145 | 2.174006782 | 0.575 | 0.288 | 7.62E-141 | Neutrophil |
| Mrc1 | 3.38E-222 | 2.767744222 | 0.336 | 0.026 | 6.92E-218 | Macrophage |
| Mt1 | 2.13E-126 | 2.350910243 | 0.802 | 0.64 | 4.36E-122 | Macrophage |
| H2-Eb1 | 1.33E-73 | 3.26938559 | 0.333 | 0.111 | 2.73E-69 | Macrophage |
| H2-Aa | 6.59E-71 | 3.005436384 | 0.346 | 0.123 | 1.35E-66 | Macrophage |
| Mt2 | 6.93E-71 | 2.060389359 | 0.549 | 0.328 | 1.42E-66 | Macrophage |
| Cd74 | 9.60E-49 | 2.698608477 | 0.411 | 0.22 | 1.97E-44 | Macrophage |
| H2-Ab1 | 9.91E-49 | 2.894432959 | 0.319 | 0.137 | 2.03E-44 | Macrophage |
| Apoe | 2.93E-06 | 2.098183211 | 0.385 | 0.352 | 0.060101691 | Macrophage |
| Ms4a6c1 | 0 | 2.581261043 | 0.947 | 0.1 | 0 | Monocyte |
| Gngt2 | 0 | 2.320538361 | 0.845 | 0.17 | 0 | Monocyte |
| Ctss1 | 0 | 2.233130796 | 0.979 | 0.419 | 0 | Monocyte |
| Apoc2 | 0 | 2.211347505 | 0.789 | 0.101 | 0 | Monocyte |
| Mafb1 | 0 | 2.144336033 | 0.714 | 0.064 | 0 | Monocyte |

| Ms4a6d1 | 0 | 2.020476957 | 0.797 | 0.082 | 0 | Monocyte |
| --- | --- | --- | --- | --- | --- | --- |
| Ctsb1 | 0 | 2.013263592 | 0.988 | 0.479 | 0 | Monocyte |
| Sdc41 | 2.41E-307 | 2.148927836 | 0.734 | 0.15 | 4.93E-303 | Monocyte |
| Apoe1 | 7.30E-259 | 2.050300205 | 0.814 | 0.28 | 1.50E-254 | Monocyte |
| Tgfbi1 | 9.48E-249 | 2.179266749 | 0.88 | 0.388 | 1.94E-244 | Monocyte |
| Saa31 | 5.50E-167 | 3.215166324 | 0.776 | 0.413 | 1.13E-162 | Monocyte |
| Gzma | 0 | 5.14294679 | 0.985 | 0.093 | 0 | NK_cell |
| Ccl5 | 0 | 4.234154313 | 1 | 0.229 | 0 | NK_cell |
| Nkg7 | 0 | 3.779302258 | 0.99 | 0.101 | 0 | NK_cell |
| AW112010 | 0 | 3.630208213 | 0.992 | 0.286 | 0 | NK_cell |
| Irf8 | 0 | 3.62023094 | 0.974 | 0.151 | 0 | NK_cell |
| Gzmb | 0 | 3.485440703 | 0.951 | 0.082 | 0 | NK_cell |
| Prf1 | 0 | 3.463333303 | 0.914 | 0.032 | 0 | NK_cell |
| Klra4 | 0 | 2.959221078 | 0.523 | 0.006 | 0 | NK_cell |
| Il2rb | 0 | 2.902121094 | 0.917 | 0.082 | 0 | NK_cell |
| Klra8 | 0 | 2.804419231 | 0.554 | 0.005 | 0 | NK_cell |
| Klre1 | 0 | 2.63774051 | 0.778 | 0.017 | 0 | NK_cell |
| Klrb1c | 0 | 2.58345199 | 0.791 | 0.012 | 0 | NK_cell |
| Klrk1 | 0 | 2.54252883 | 0.839 | 0.057 | 0 | NK_cell |
| Klrd1 | 0 | 2.51605932 | 0.833 | 0.062 | 0 | NK_cell |
| Serpinb9 | 0 | 2.355527494 | 0.765 | 0.062 | 0 | NK_cell |
| Serpinb6b | 0 | 2.1770969 | 0.726 | 0.081 | 0 | NK_cell |
| Ugcg | 0 | 2.167324831 | 0.888 | 0.244 | 0 | NK_cell |
| Klra9 | 0 | 2.116337904 | 0.419 | 0.001 | 0 | NK_cell |
| Klra7 | 0 | 2.097280314 | 0.487 | 0.005 | 0 | NK_cell |
| Txk | 0 | 2.067860198 | 0.727 | 0.07 | 0 | NK_cell |
| Ifng | 0 | 2.062613044 | 0.612 | 0.049 | 0 | NK_cell |
| Xcl1 | 4.16E-295 | 2.188596617 | 0.343 | 0.009 | 8.51E-291 | NK_cell |
| Lgals1 | 1.41E-228 | 2.262061472 | 0.825 | 0.277 | 2.88E-224 | NK_cell |
| Trbc2 | 0 | 3.408732717 | 0.864 | 0.068 | 0 | T_cell |
| Cd3d | 0 | 3.067719892 | 0.859 | 0.03 | 0 | T_cell |
| Cd3g | 0 | 2.935955957 | 0.81 | 0.016 | 0 | T_cell |
| Il7r | 0 | 2.301716819 | 0.539 | 0.038 | 0 | T_cell |
| Ms4a4b1 | 0 | 2.243593017 | 0.773 | 0.14 | 0 | T_cell |
| Trac | 0 | 2.117978076 | 0.711 | 0.027 | 0 | T_cell |
| Cd8b1 | 0 | 2.113571426 | 0.389 | 0.01 | 0 | T_cell |
| Gramd31 | 0 | 2.055731227 | 0.716 | 0.067 | 0 | T_cell |
| Cd3e | 0 | 2.001763866 | 0.675 | 0.007 | 0 | T_cell |
| Rpl121 | 2.37E-253 | 2.12302041 | 0.99 | 0.645 | 4.85E-249 | T_cell |
| Trbc11 | 4.69E-177 | 2.17603564 | 0.509 | 0.097 | 9.60E-173 | T_cell |
| Ctla2a2 | 1.14E-128 | 2.392506808 | 0.639 | 0.213 | 2.33E-124 | T_cell |
| Igkc | 0 | 6.076780641 | 0.957 | 0.046 | 0 | B_cell |
| Cd79a | 0 | 4.721599046 | 0.993 | 0.005 | 0 | B_cell |
| Ly6d | 0 | 4.023196785 | 0.878 | 0.003 | 0 | B_cell |
| Ebf1 | 0 | 3.827402403 | 0.964 | 0.007 | 0 | B_cell |
| Iglc2 | 0 | 3.77955772 | 0.813 | 0.003 | 0 | B_cell |
| Cd79b | 0 | 3.15684253 | 0.856 | 0.008 | 0 | B_cell |
| Fcmr | 0 | 2.919074837 | 0.842 | 0.002 | 0 | B_cell |
| Ms4a1 | 0 | 2.812514714 | 0.842 | 0.001 | 0 | B_cell |
| Iglc1 | 0 | 2.609528358 | 0.403 | 0.002 | 0 | B_cell |
| H2-DMb2 | 0 | 2.551037734 | 0.77 | 0.011 | 0 | B_cell |
| Iglc3 | 0 | 2.49176771 | 0.662 | 0.002 | 0 | B_cell |
| Ighd | 0 | 2.442544715 | 0.755 | 0.003 | 0 | B_cell |
| H2-Ob | 0 | 2.251206439 | 0.777 | 0.006 | 0 | B_cell |
| Scd1 | 0 | 2.10255343 | 0.691 | 0.021 | 0 | B_cell |
| Mzb1 | 0 | 2.078923216 | 0.669 | 0.002 | 0 | B_cell |
| Ccr7 | 8.37E-294 | 2.064853366 | 0.799 | 0.041 | 1.71E-289 | B_cell |
| Ighm | 2.13E-261 | 4.223885492 | 0.971 | 0.096 | 4.37E-257 | B_cell |

| Mef2c | 1.45E-237 | 2.849196047 | 0.856 | 0.074 | 2.96E-233 | B_cell |
| --- | --- | --- | --- | --- | --- | --- |
| H2-Eb11 | 6.49E-190 | 2.566180422 | 0.971 | 0.122 | 1.33E-185 | B_cell |
| H2-Aa1 | 2.28E-181 | 2.867463601 | 0.978 | 0.134 | 4.66E-177 | B_cell |
| H2-Ab11 | 2.51E-176 | 2.974174798 | 0.978 | 0.142 | 5.14E-172 | B_cell |
| Gm83691 | 1.35E-162 | 2.230624227 | 0.755 | 0.078 | 2.76E-158 | B_cell |
| Cd741 | 1.63E-137 | 3.616204556 | 1 | 0.228 | 3.35E-133 | B_cell |
| **0 means that the p value is less than lower limit**  **Genes with avg_log2FC more than 2 have been shown in the table** | | | | | | |

| **Supplementary Table. 5**  **NET-related genes** | | | |
| --- | --- | --- | --- |
| SGK1 | CXCR4 | KLF2 | PKM |
| ACTB | CYBB | KRT10 | PROCR |
| ACTG1 | DEFA3 | LCP1 | PRTN3 |
| ACTN1 | DNAJB1 | LDLR | PTAFR |
| ACTN4 | DNASE1 | LPAR3 | RIPK1 |
| AKT1 | ELANE | LTF | RIPK3 |
| AKT2 | ENO1 | LYZ | S100A12 |
| ARPIN | ENTPD4 | MAPK1 | S100A8 |
| ATG7 | F2RL2 | MAPK14 | S100A9 |
| AZU1 | F3 | MAPK3 | S1PR2 |
| C3 | FCAR | MAPK7 | SELP |
| C3AR1 | FCGR2B | MCOLN3 | SELPLG |
| C5AR1 | FGL2 | MFN1 | SGK1 |
| CAMP | GPBAR1 | MFN2 | SIGLEC14 |
| CARD11 | GSDMD | MIR146A | SOCS3 |
| CASP1 | H2AX | MIR21 | SPP1 |
| CAT | HIF1A | MIR223 | SRC |
| CCDC25 | HMGB1 | MMP9 | STAT3 |
| CCL2 | HRG | MNDA | SUCNR1 |
| CCL3 | IL12A | MPO | SYK |
| CCL4 | IL17A | MTOR | TICAM1 |
| CCL5 | IL1B | MYD88 | TIMP1 |
| CD177 | IL1RL1 | MYH9 | TKT |
| CD274 | IL33 | NFE2L2 | TLR2 |
| CD44 | IL36RN | NFIL3 | TLR4 |
| CEBPB | IL5 | NFKBIA | TLR7 |
| CFTR | IL6 | NLRP3 | TLR8 |
| CLEC4E | IL8 | NOX4 | TLR9 |
| CLEC6A | ILK | OPA1 | TNF |
| CLEC7A | IRAK4 | ORAI1 | TNFAIP3 |
| CSF3 | IRF1 | P2RX1 | WASL |
| CTSC | ITGAM | PADI4 | XIST |
| CTSG | ITGB1 | PARVB |  |
| CXCL1 | ITGB2 | PF4 |  |
| CXCL2 | KCNN3 | PIK3CA |  |

| **Supplementary Table. 6** |
| --- |
| **MAPK negative feedback** |
| DUSP1 |
| DUSP10 |
| DUSP14 |
| DUSP16 |
| DUSP2 |
| DUSP3 |
| DUSP4 |
| DUSP5 |
| DUSP6 |
| DUSP7 |
| DUSP8 |
| DUSP9 |

| **Supplementary Table. 7** | | | | |
| --- | --- | --- | --- | --- |
| **Glycolysis / Gluconeogenesis** | | | | |
| HK3 | HK1 | HK2 | HKDC1 | GCK |
| GPI | PFKM | PFKP | PFKL | FBP1 |
| FBP2 | ALDOC | ALDOA | ALDOB | TPI1 |
| GAPDH | GAPDHS | PGK2 | PGK1 | PGAM1 |
| PGAM2 | PGAM4 | ENO3 | ENO2 | ENO1 |
| ENO4 | PKM | PKLR | PDHA2 | PDHA1 |
| PDHB | DLAT | DLD | LDHAL6A | LDHAL6B |
| LDHA | LDHB | LDHC | ADH1A | ADH1B |
| ADH1C | ADH7 | ADH4 | ADH5 | ADH6 |
| AKR1A1 | ALDH2 | ALDH3A2 | ALDH1B1 | ALDH7A1 |
| ALDH9A1 | ALDH3B1 | ALDH3B2 | ALDH1A3 | ALDH3A1 |
| ACSS1 | ACSS2 | GALM | PGM1 | PGM2 |
| G6PC | G6PC2 | G6PC3 | ADPGK | BPGM |
| MINPP1 | PCK1 | PCK2 |  |  |
| **Citrate cycle (TCA cycle)** | | | | |
| CS | ACLY | ACO2 | ACO1 | IDH1 |
| IDH2 | IDH3B | IDH3G | IDH3A | OGDHL |
| OGDH | DLST | DLD | SUCLG1 | SUCLG2 |
| SUCLA2 | SDHA | SDHB | SDHC | SDHD |
| FH | MDH1 | MDH2 | PC | PCK1 |
| PCK2 | PDHA2 | PDHA1 | PDHB | DLAT |
| **Oxidative phosphorylation** | | | | |
| NDUFS6 | NDUFS7 | NDUFS8 | NDUFV1 | NDUFV2 |
| NDUFV3 | NDUFA1 | NDUFA2 | NDUFA3 | NDUFA4 |
| NDUFA4L2 | NDUFA5 | NDUFA6 | NDUFA7 | NDUFA8 |
| NDUFA9 | NDUFA10 | NDUFAB1 | NDUFA11 | NDUFA12 |
| NDUFA13 | NDUFB1 | NDUFB2 | NDUFB3 | NDUFB4 |
| NDUFB5 | NDUFB6 | NDUFB7 | NDUFB8 | NDUFB9 |
| NDUFB10 | NDUFB11 | NDUFC1 | NDUFC2 | NDUFC2-KCTD14 |
| SDHA | SDHB | SDHC | SDHD | UQCRFS1 |
| CYTB | CYC1 | UQCRC1 | UQCRC2 | UQCRH |
| UQCRHL | UQCRB | UQCRQ | UQCR10 | UQCR11 |
| COX10 | COX3 | COX1 | COX2 | COX4I2 |
| COX4I1 | COX5A | COX5B | COX6A1 | COX6A2 |
| COX6B1 | COX6B2 | COX6C | COX7A1 | COX7A2 |
| COX7A2L | COX7B | COX7B2 | COX7C | COX8C |
| COX8A | COX11 | COX15 | COX17 | ATP5F1A |
| ATP5F1B | ATP5F1C | ATP5F1D | ATP5F1E | ATP5PO |
| ATP6 | ATP5PB | ATP5MC1 | ATP5MC2 | ATP5MC3 |
| ATP5PD | ATP5ME | ATP5MF | ATP5MG | ATP5PF |
| ATP8 | ATP6V1A | ATP6V1B1 | ATP6V1B2 | ATP6V1C2 |
| ATP6V1C1 | ATP6V1D | ATP6V1E2 | ATP6V1E1 | ATP6V1F |
| ATP6V1G1 | ATP6V1G3 | ATP6V1G2 | ATP6V1H | TCIRG1 |
| ATP6V0A2 | ATP6V0A4 | ATP6V0A1 | ATP6V0C | ATP6V0B |
| ATP6V0D1 | ATP6V0D2 | ATP6V0E1 | ATP6V0E2 | ATP6AP1 |
| ATP4A | ATP4B | ATP12A | PPA2 | PPA1 |
| LHPP |  |  |  |  |

| **Supplementary Table. 8** | | | | |
| --- | --- | --- | --- | --- |
| **A total of 504 autophagy-related genes from the Molecular Signatures Database** | | | | |
| ATG5 | LRRK2 | ADRB2 | NPRL2 | TP53INP2 |
| ATP13A2 | LZTS1 | AMBRA1 | NPRL3 | TPCN1 |
| BAG3 | MAGEA3 | ATF6 | OPTN | TRIM13 |
| CLU | MAGEA6 | ATG16L1 | ORMDL3 | TRIM14 |
| CTSA | MCL1 | ATG2A | PAFAH1B2 | TRIM21 |
| EEF1A1 | MET | BAD | PARK7 | TRIM22 |
| EEF1A2 | MIR199A1 | BCL2L11 | PIK3C2A | TRIM27 |
| GFAP | MIRLET7B | BNIP3 | PIK3CB | TRIM32 |
| HSP90AA | MT3 | BNIP3L | PIM2 | TRIM34 |
| HSPA8 | MTM1 | C9orf72 | PIP4K2A | TRIM38 |
| LAMP2 | MTMR8 | CALCOCO2 | PIP4K2B | TRIM5 |
| PLK3 | MTMR9 | CAMKK2 | PIP4K2C | TRIM6 |
| SNCA | MTOR | CERS1 | PLEKHF1 | TRIM65 |
| SNRNP70 | NPC1 | DAPK1 | PLK2 | TRIM68 |
| STUB1 | NRBP2 | DCN | PRKAA1 | TRIM8 |
| SYNPO2 | NUPR1 | DEPDC5 | PRKAA2 | TRIML1 |
| ADRA1A | PHF23 | DHRSX | PRKD1 | TRIML2 |
| AKT1 | PIK3CA | ELAPOR1 | PRKN | UFL1 |
| ATG7 | PINK1 | ENDOG | RAB3GAP1 | ULK1 |
| BCL2 | POLDIP2 | EPM2A | RAB3GAP2 | ULK2 |
| BECN1 | PTPN22 | FBXO7 | RALB | UVRAG |
| BMF | QSOX1 | FLCN | RIPK2 | VDAC1 |
| CHMP4A | RASIP1 | FOXO1 | RNF152 | VPS13D |
| CHMP4B | RNF41 | FOXO3 | RNF31 | WAC |
| CLEC16A | RNF5 | FYCO1 | ROCK1 | WDR45 |
| CPTP | RRAGA | GNAI3 | RUFY4 | WIPI1 |
| DAP | RUBCN | GPSM1 | SCOC | ZC3H12A |
| DAPL1 | SCFD1 | GSK3A | SESN1 | ABL1 |
| EHMT2 | SEC22B | GSK3B | SESN2 | ABL2 |
| EIF4E | SIRT2 | HIF1A | SESN3 | ACER2 |
| EIF4G1 | SMCR8 | HMGB1 | SH3BP4 | ATG12 |
| EIF4G2 | SMG1 | HSPB8 | SH3GLB1 | ATG14 |
| EIF4G3 | STAT3 | HTT | SIRT1 | ATM |
| FEZ1 | TAB2 | IFNG | SLC25A4 | ATP6V0A1 |
| FEZ2 | TAB3 | IKBKG | SLC25A5 | ATP6V0A2 |
| FOXK1 | TBC1D14 | IL4 | SNX18 | ATP6V0B |
| FOXK2 | TIGAR | IRGM | SNX30 | ATP6V0C |
| GATA4 | TLK2 | KAT5 | SNX4 | ATP6V0D1 |
| GOLGA2 | TMEM39A | KDR | SNX7 | ATP6V0D2 |
| HERC1 | TP53 | LACRT | SPTLC1 | ATP6V0E1 |
| HGF | TREM2 | LARP1 | SPTLC2 | ATP6V0E2 |
| HMOX1 | TSC1 | LRSAM1 | STING1 | ATP6V1A |
| HTR2B | TSC2 | MAP3K7 | STK11 | ATP6V1B1 |
| IL10 | TSPO | MAPK3 | SUPT5H | ATP6V1B2 |
| IL10RA | UBQLN4 | MEFV | SVIP | ATP6V1C1 |
| KDM4A | USP30 | MID2 | TBK1 | ATP6V1C2 |
| KIF25 | USP36 | MOAP1 | TFEB | ATP6V1D |
| KLHL22 | WASHC1 | MTDH | TICAM1 | ATP6V1E1 |
| LEP | WDR6 | NOD1 | TMEM59 | ATP6V1E2 |
| LEPR | ZKSCAN3 | NOD2 | TP53INP1 | ATP6V1G1 |
| ATP6V1G | MTMR3 | MUL1 | IFNA6 | PLIN3 |
| ATP6V1H | MTMR4 | TOMM7 | IFNA7 | PRKAB1 |
| BOK | NEDD4 | ACBD5 | IFNA8 | PRKAB2 |
| CAPN1 | NLRP6 | ARFIP2 | INS | PRKAG1 |
| CAPNS1 | OSBPL7 | ATG13 | ULK3 | PRKAG2 |
| CASP3 | PARL | ATG2B | ARL13B | PRKAG3 |

| CDK5 | PIK3C3 | ATG4B | ATG10 | RNASE1 |
| --- | --- | --- | --- | --- |
| CDK5R1 | PIK3R2 | ATG4D | ATG101 | RPS27A |
| CISD1 | PIK3R4 | AUP1 | ATG9A | SLC38A9 |
| CISD2 | PRKACA | C5orf51 | ATG9B | SRC |
| CLN3 | PSAP | DDRGK1 | CETN1 | TOMM20 |
| CRYBA1 | PYCARD | KLHL3 | CFTR | TOMM22 |
| CSNK2A2 | RAB39B | LGALS8 | CHMP2A | TOMM40 |
| CTTN | RAB8A | LRBA | CHMP2B | TOMM5 |
| DAPK2 | RHEB | MAP1LC3B | CHMP3 | TOMM6 |
| DAPK3 | RMC1 | MAP1LC3C | CHMP4C | TOMM70 |
| DCAF12 | RPTOR | PHB2 | CHMP6 | TSG101 |
| DDIT3 | RRAGB | PJVK | CHMP7 | TUBA1A |
| DEPP1 | RRAGC | RAB7A | CSNK2A1 | TUBA1B |
| DNM1L | RRAGD | RB1CC1 | CSNK2B | TUBA1C |
| DRAM1 | SNX32 | RETREG1 | DYNC1H1 | TUBA3C |
| DRAM2 | SNX5 | RETREG3 | DYNC1I1 | TUBA3D |
| EP300 | SNX6 | RNF213 | DYNC1I2 | TUBA3E |
| ERCC4 | SOGA1 | SQSTM1 | DYNC1LI1 | TUBA4A |
| ERN1 | SOGA3 | STBD1 | DYNC1LI2 | TUBA4B |
| EXOC1 | SREBF1 | TAFAZZIN | DYNLL1 | TUBA8 |
| EXOC4 | SREBF2 | TEX264 | DYNLL2 | TUBAL3 |
| EXOC7 | TBC1D25 | UBA5 | EPAS1 | TUBB1 |
| EXOC8 | TECPR1 | UFC1 | FUNDC1 | TUBB2A |
| FBXL2 | TPCN2 | UFM1 | HBB | TUBB2B |
| FBXW7 | TRIB3 | WDFY3 | HSF1 | TUBB3 |
| FZD5 | UBQLN1 | WDR81 | HSP90AB1 | TUBB4A |
| GAPDH | UBQLN2 | WIPI2 | IFT88 | TUBB4B |
| GBA | UCHL1 | ATG3 | LAMTOR1 | TUBB6 |
| GPR137 | USP10 | ATG4A | LAMTOR2 | TUBB8 |
| GPR137B | USP13 | ATG4C | LAMTOR3 | TUBB8B |
| HAX1 | USP33 | BECN2 | LAMTOR4 | UBA52 |
| HDAC6 | VPS13C | GABARAP | LAMTOR5 | UBAP1 |
| HSPB1 | VPS26A | GABARAPL1 | MAP1LC3A | UBB |
| HTRA2 | VPS26B | GABARAPL2 | MFN1 | UBC |
| IFI16 | VPS29 | IFNA1 | MLST8 | UBE2N |
| ITPR1 | VPS35 | IFNA10 | MTERF3 | UBE2V1 |
| KAT8 | WDR24 | IFNA13 | MTMR14 | VCP |
| KEAP1 | WDR41 | IFNA14 | MVB12A | VIM |
| LAMP3 | ZMPSTE24 | IFNA16 | MVB12B | VPS28 |
| MAPK15 | ATP5IF1 | IFNA17 | NBR1 | VPS37A |
| MAPK8 | CDC37 | IFNA2 | PCNT | VPS37B |
| MAPT | HK2 | IFNA21 | PEX5 | VPS37C |
| MFSD8 | HUWE1 | IFNA4 | PGAM5 | VPS37D |
| MTCL1 | MFN2 | IFNA5 | PLIN2 | WDR45B |
| AKT1S1 | DEPTOR | ATG16L2 | ZFYVE1 |  |

| **Supplementary Table. 9** | | | | |
| --- | --- | --- | --- | --- |
| **29 mitophagy-related genes extracted from Reactome Pathway Database** | | | | |
| ATG12 | ATG5 | CSNK2A1 | CSNK2A2 | CSNK2B |
| FUNDC1 | MAP1LC3A | MAP1LC3B | MFN1 | MFN2 |
| MTERF3 | PGAM5 | PINK1 | PARK2 | RPS27A |
| SQSTM1 | SRC | TOMM20 | TOMM22 | TOMM40 |
| TOMM5 | TOMM6 | TOMM7 | TOMM70A | UBA52 |
| UBB | UBC | ULK1 | VDAC1 |  |

| **Supplementary Table. 10** |
| --- |
| **PANoptosome-related genes** |
| ZBP1 |
| NLRP3 |
| RIPK1 |
| RIPK3 |
| CASP1 |
| CASP6 |
| CASP8 |
| PYCARD |
| FADD |
| MAP3K7 |
| TNFAIP3 |
| RNF31 |
| RBCK1 |
| PSTPIP2 |

| **Supplementary Table. 11 Marker genes of S100A8/A9hi neutrophils** | | | | | |
| --- | --- | --- | --- | --- | --- |
| **gene** | **p_val** | **avg_log2FC** | **pct.1** | **pct.2** | **p_val_adj** |
| Camp | 0 | 3.557375038 | 0.967 | 0.176 | 0 |
| Ltf | 0 | 3.346052949 | 0.941 | 0.141 | 0 |
| Ngp | 0 | 2.931882815 | 1 | 0.417 | 0 |
| Cd177 | 1.58E-278 | 2.078453431 | 0.989 | 0.354 | 3.23E-274 |
| Serpinb1a | 1.16E-274 | 2.059002983 | 0.901 | 0.165 | 2.38E-270 |
| Ifitm6 | 8.28E-270 | 2.118577801 | 1 | 0.681 | 1.70E-265 |
| Chil3 | 9.46E-267 | 2.322632687 | 0.99 | 0.496 | 1.94E-262 |
| Adpgk | 1.55E-242 | 1.647507173 | 0.784 | 0.11 | 3.18E-238 |
| Arhgdib | 9.97E-242 | 1.352891891 | 0.998 | 0.81 | 2.04E-237 |
| Anxa1 | 6.58E-224 | 1.541206283 | 1 | 0.771 | 1.35E-219 |
| Cybb | 3.70E-214 | 1.844484845 | 0.86 | 0.222 | 7.59E-210 |
| Syne1 | 5.08E-214 | 1.594978729 | 0.893 | 0.249 | 1.04E-209 |
| Dstn | 2.18E-193 | 1.39142907 | 0.987 | 0.555 | 4.47E-189 |
| S100a9 | 6.96E-181 | 0.693430324 | 1 | 0.999 | 1.43E-176 |
| Itgb2l | 1.65E-178 | 1.116444391 | 0.7 | 0.112 | 3.38E-174 |
| Aldh2 | 1.06E-176 | 1.360878217 | 0.917 | 0.418 | 2.18E-172 |
| S100a8 | 8.56E-170 | 0.738591794 | 1 | 0.999 | 1.75E-165 |
| Ckap4 | 1.48E-165 | 1.295102854 | 0.874 | 0.303 | 3.04E-161 |
| Lamtor4 | 2.47E-157 | 1.198481205 | 0.872 | 0.33 | 5.05E-153 |
| Mmp8 | 4.26E-154 | 1.327124557 | 0.987 | 0.633 | 8.73E-150 |
| mt-Co2 | 1.96E-150 | 1.032607247 | 0.986 | 0.852 | 4.01E-146 |
| Tkt | 2.48E-146 | 1.122685375 | 0.914 | 0.443 | 5.07E-142 |
| mt-Co3 | 8.35E-146 | 1.098168534 | 0.994 | 0.849 | 1.71E-141 |
| Alox5ap | 6.51E-140 | 0.779135143 | 1 | 0.947 | 1.33E-135 |
| mt-Atp6 | 9.04E-140 | 0.977892091 | 0.993 | 0.882 | 1.85E-135 |
| Anxa3 | 5.31E-139 | 1.052529029 | 0.7 | 0.173 | 1.09E-134 |
| Lcn2 | 1.06E-133 | 0.948890796 | 1 | 0.921 | 2.17E-129 |
| Abca13 | 1.11E-130 | 0.905442719 | 0.533 | 0.077 | 2.26E-126 |
| Flna | 4.14E-128 | 1.030398042 | 0.945 | 0.539 | 8.48E-124 |
| Cyba | 1.17E-127 | 0.746125369 | 0.995 | 0.932 | 2.40E-123 |
| AA467197 | 9.16E-125 | 1.019432479 | 0.945 | 0.514 | 1.88E-120 |
| Scrg1 | 1.23E-124 | 0.962231321 | 0.652 | 0.16 | 2.51E-120 |
| Lgals3 | 1.28E-121 | 0.906467578 | 0.983 | 0.759 | 2.63E-117 |
| Ldha | 1.57E-120 | 1.049366811 | 0.908 | 0.484 | 3.21E-116 |
| Capg | 5.60E-119 | 0.979112533 | 0.712 | 0.213 | 1.15E-114 |
| Rflnb | 1.46E-117 | 0.97040011 | 0.715 | 0.222 | 2.99E-113 |
| Ak2 | 2.46E-117 | 0.926492083 | 0.696 | 0.198 | 5.03E-113 |
| Flot2 | 5.84E-117 | 0.904839999 | 0.667 | 0.182 | 1.20E-112 |
| mt-Co1 | 8.45E-117 | 0.749135963 | 0.996 | 0.908 | 1.73E-112 |
| Ly6c2 | 4.50E-112 | 0.857878536 | 0.919 | 0.473 | 9.21E-108 |
| Mmp25 | 9.31E-112 | 0.864585216 | 0.718 | 0.219 | 1.91E-107 |
| Rbm3 | 5.36E-111 | 0.93825194 | 0.932 | 0.541 | 1.10E-106 |
| Gpi1 | 2.47E-110 | 0.856456469 | 0.966 | 0.64 | 5.06E-106 |
| Trak2 | 1.70E-104 | 0.729916543 | 0.493 | 0.089 | 3.49E-100 |
| Nucb2 | 7.29E-103 | 0.768529836 | 0.498 | 0.092 | 1.49E-98 |
| Hmgn2 | 8.04E-103 | 1.016441295 | 0.859 | 0.444 | 1.65E-98 |
| Acvrl1 | 2.55E-99 | 0.918989458 | 0.485 | 0.098 | 5.23E-95 |
| 1-Sep | 1.45E-98 | 0.610756984 | 0.407 | 0.054 | 2.96E-94 |
| Aprt | 4.53E-97 | 0.734583407 | 0.556 | 0.135 | 9.28E-93 |
| Man2a1 | 2.56E-96 | 0.696413951 | 0.724 | 0.228 | 5.23E-92 |
| Ndufb7 | 1.97E-95 | 0.825431121 | 0.829 | 0.371 | 4.04E-91 |
| Nin | 7.76E-93 | 0.732224288 | 0.54 | 0.135 | 1.59E-88 |
| Rpsa | 1.49E-91 | 0.907494861 | 0.88 | 0.514 | 3.05E-87 |
| Lta4h | 3.22E-91 | 0.710408668 | 0.401 | 0.061 | 6.59E-87 |
| Rps17 | 1.90E-90 | 0.802884565 | 0.749 | 0.314 | 3.88E-86 |
| Tmem256 | 5.57E-90 | 0.628236672 | 0.385 | 0.056 | 1.14E-85 |

| G6pdx | 1.78E-89 | 0.786337454 | 0.681 | 0.247 | 3.64E-85 |
| --- | --- | --- | --- | --- | --- |
| Pglyrp1 | 2.44E-89 | 0.627961166 | 0.995 | 0.897 | 4.99E-85 |
| Ldhc | 1.46E-87 | 0.701155705 | 0.275 | 0.014 | 2.99E-83 |
| Cd63 | 1.59E-87 | 0.656635546 | 0.856 | 0.408 | 3.26E-83 |
| Tpr | 1.70E-87 | 0.782671404 | 0.661 | 0.228 | 3.48E-83 |
| Pygl | 2.07E-86 | 0.711533835 | 0.975 | 0.74 | 4.24E-82 |
| Ltb4r1 | 2.21E-85 | 0.749097525 | 0.757 | 0.316 | 4.52E-81 |
| Rpl36al | 9.08E-85 | 0.705913817 | 0.584 | 0.178 | 1.86E-80 |
| Hist1h1e | 6.25E-84 | 0.867335209 | 0.405 | 0.076 | 1.28E-79 |
| Ppia | 3.79E-83 | 0.856992965 | 0.836 | 0.432 | 7.76E-79 |
| Tmem160 | 7.10E-83 | 0.597413764 | 0.446 | 0.094 | 1.45E-78 |
| Dach1 | 9.18E-83 | 0.692900478 | 0.444 | 0.095 | 1.88E-78 |
| F630028O | 3.38E-82 | 0.785959601 | 0.863 | 0.45 | 6.92E-78 |
| Pomp | 3.23E-79 | 0.731760624 | 0.838 | 0.414 | 6.61E-75 |
| Tecr | 3.43E-79 | 0.661969849 | 0.581 | 0.186 | 7.03E-75 |
| Myl6 | 7.03E-79 | 0.547420524 | 0.993 | 0.901 | 1.44E-74 |
| Tbcb | 9.60E-78 | 0.582125801 | 0.484 | 0.123 | 1.97E-73 |
| Anxa5 | 3.93E-77 | 0.600687968 | 0.44 | 0.101 | 8.05E-73 |
| Ndufv3 | 4.77E-77 | 0.592385388 | 0.444 | 0.103 | 9.77E-73 |
| Ap3s1 | 2.62E-76 | 0.695994099 | 0.691 | 0.28 | 5.37E-72 |
| Degs1 | 5.00E-75 | 0.713513387 | 0.753 | 0.333 | 1.02E-70 |
| Lipg | 1.05E-74 | 0.546173131 | 0.749 | 0.286 | 2.15E-70 |
| Olfml2b | 2.47E-74 | 0.531924963 | 0.312 | 0.04 | 5.06E-70 |
| Tbc1d8 | 4.36E-74 | 0.531471986 | 0.337 | 0.052 | 8.94E-70 |
| mt-Cytb | 1.40E-73 | 0.660419903 | 0.948 | 0.662 | 2.86E-69 |
| Dbnl | 2.35E-72 | 0.630792869 | 0.662 | 0.257 | 4.81E-68 |
| Pcmt1 | 2.84E-72 | 0.605305897 | 0.529 | 0.165 | 5.81E-68 |
| Mapk13 | 3.33E-72 | 0.691688902 | 0.699 | 0.296 | 6.82E-68 |
| Lbr | 8.65E-72 | 0.688467368 | 0.918 | 0.569 | 1.77E-67 |
| Mmp9 | 1.06E-71 | 0.667015125 | 0.985 | 0.834 | 2.18E-67 |
| Napsa | 1.21E-71 | 0.668221243 | 0.825 | 0.425 | 2.47E-67 |
| Ndufb10 | 1.58E-71 | 0.555280986 | 0.495 | 0.138 | 3.23E-67 |
| Sec61g | 8.15E-71 | 0.731492214 | 0.742 | 0.351 | 1.67E-66 |
| Atp5h | 3.31E-70 | 0.648743165 | 0.849 | 0.472 | 6.78E-66 |
| Slc25a24 | 2.71E-69 | 0.565676469 | 0.449 | 0.118 | 5.55E-65 |
| Rpl32 | 1.58E-68 | 0.68743502 | 0.883 | 0.562 | 3.24E-64 |
| Atp5o | 2.82E-68 | 0.52425817 | 0.448 | 0.118 | 5.79E-64 |
| Arrb2 | 3.05E-68 | 0.651359073 | 0.735 | 0.33 | 6.25E-64 |
| Rps20 | 3.43E-68 | 0.656242703 | 0.885 | 0.533 | 7.03E-64 |
| Pdia3 | 5.48E-68 | 0.591694032 | 0.592 | 0.214 | 1.12E-63 |
| AI662270 | 6.78E-68 | 0.518026924 | 0.345 | 0.063 | 1.39E-63 |
| Ndufa11 | 4.81E-67 | 0.533663346 | 0.411 | 0.099 | 9.85E-63 |
| Rpl3 | 6.47E-67 | 0.576619561 | 0.65 | 0.25 | 1.32E-62 |
| Atp5g3 | 1.04E-66 | 0.502976397 | 0.489 | 0.143 | 2.14E-62 |
| Cd47 | 1.75E-66 | 0.56272457 | 0.962 | 0.725 | 3.59E-62 |
| Rpl10a | 1.64E-65 | 0.567944252 | 0.605 | 0.225 | 3.36E-61 |
| Krt83 | 1.72E-65 | 0.716210042 | 0.322 | 0.059 | 3.52E-61 |
| mt-Nd1 | 3.22E-65 | 0.60340245 | 0.773 | 0.36 | 6.60E-61 |
| Rasa2 | 5.24E-65 | 0.684452024 | 0.817 | 0.419 | 1.07E-60 |
| Ppm1m | 6.09E-65 | 0.523968882 | 0.372 | 0.085 | 1.25E-60 |
| Stfa2 | 8.14E-64 | 1.589201457 | 0.417 | 0.116 | 1.67E-59 |
| Thbs1 | 1.05E-63 | 0.786318736 | 0.956 | 0.636 | 2.15E-59 |
| Cebpe | 2.53E-63 | 0.583183162 | 0.323 | 0.061 | 5.17E-59 |
| Ncf1 | 2.55E-63 | 0.597188076 | 0.938 | 0.614 | 5.22E-59 |
| Srp9 | 4.88E-63 | 0.527456175 | 0.683 | 0.295 | 1.00E-58 |
| Cntrl | 7.44E-63 | 0.550147993 | 0.496 | 0.158 | 1.52E-58 |
| Hsd11b1 | 8.85E-63 | 0.567065524 | 0.572 | 0.208 | 1.81E-58 |
| Mpc2 | 3.48E-62 | 0.593651997 | 0.666 | 0.289 | 7.14E-58 |

| S100a13 | 4.39E-62 | 0.533641625 | 0.65 | 0.262 | 8.98E-58 |
| --- | --- | --- | --- | --- | --- |
| Rps18 | 7.72E-62 | 0.605138688 | 0.804 | 0.441 | 1.58E-57 |
| Plbd1 | 7.73E-62 | 0.622952667 | 0.797 | 0.41 | 1.58E-57 |
| Pnkp | 1.30E-61 | 0.645085617 | 0.71 | 0.341 | 2.67E-57 |
| Ywhab | 1.46E-61 | 0.591609701 | 0.81 | 0.416 | 2.99E-57 |
| Txndc17 | 1.87E-61 | 0.551147099 | 0.681 | 0.301 | 3.82E-57 |
| Eef1a1 | 4.17E-61 | 0.506201411 | 0.973 | 0.753 | 8.53E-57 |
| Plec | 3.44E-60 | 0.581916341 | 0.622 | 0.258 | 7.04E-56 |
| Tuba4a | 6.15E-60 | 0.59271406 | 0.576 | 0.218 | 1.26E-55 |
| Rps19 | 4.75E-59 | 0.670169794 | 0.829 | 0.516 | 9.73E-55 |
| Ndufb8 | 6.42E-59 | 0.536293897 | 0.528 | 0.191 | 1.31E-54 |
| Atxn10 | 2.16E-58 | 0.615427128 | 0.792 | 0.42 | 4.42E-54 |
| Padi4 | 1.06E-57 | 0.558973917 | 0.455 | 0.15 | 2.17E-53 |
| Sri | 1.93E-57 | 0.571265426 | 0.92 | 0.616 | 3.95E-53 |
| Pdcd6 | 4.67E-57 | 0.616734821 | 0.788 | 0.417 | 9.56E-53 |
| Rpl15 | 9.52E-56 | 0.594508391 | 0.845 | 0.497 | 1.95E-51 |
| Ppib | 1.82E-55 | 0.545544859 | 0.737 | 0.368 | 3.73E-51 |
| Ap2s1 | 5.59E-55 | 0.509989411 | 0.615 | 0.256 | 1.14E-50 |
| Capns1 | 7.96E-55 | 0.502855325 | 0.661 | 0.291 | 1.63E-50 |
| Sec61b | 2.37E-54 | 0.582740515 | 0.794 | 0.453 | 4.85E-50 |
| Atp8b4 | 7.99E-54 | 0.509298495 | 0.491 | 0.172 | 1.64E-49 |
| Fam107b | 1.07E-52 | 0.633486775 | 0.759 | 0.408 | 2.20E-48 |
| Acpp | 1.40E-52 | 0.530264469 | 0.36 | 0.098 | 2.87E-48 |
| 6430548M | 3.06E-52 | 0.516051327 | 0.567 | 0.231 | 6.26E-48 |
| Abhd5 | 5.92E-52 | 0.579612372 | 0.477 | 0.173 | 1.21E-47 |
| Rps4x | 6.72E-52 | 0.587833521 | 0.861 | 0.561 | 1.38E-47 |
| Npepps | 8.72E-52 | 0.523234578 | 0.559 | 0.224 | 1.79E-47 |
| Mdh2 | 1.91E-51 | 0.524230387 | 0.661 | 0.313 | 3.92E-47 |
| Cep19 | 1.97E-51 | 0.536459665 | 0.356 | 0.099 | 4.04E-47 |
| Rps8 | 2.57E-51 | 0.530356477 | 0.933 | 0.658 | 5.26E-47 |
| Rpl8 | 4.94E-51 | 0.525874176 | 0.929 | 0.687 | 1.01E-46 |
| Ceacam10 | 1.90E-50 | 0.694507241 | 0.478 | 0.188 | 3.90E-46 |
| Myh9 | 6.54E-49 | 0.530442989 | 0.964 | 0.741 | 1.34E-44 |
| Limd2 | 8.58E-49 | 0.53176683 | 0.666 | 0.324 | 1.76E-44 |
| Prok2 | 9.83E-46 | 0.514498667 | 0.47 | 0.179 | 2.01E-41 |
| H2afz | 3.19E-45 | 0.651913386 | 0.864 | 0.555 | 6.54E-41 |
| Stfa3 | 1.93E-42 | 1.420371607 | 0.31 | 0.092 | 3.94E-38 |
| Glrx | 2.52E-39 | 0.514296263 | 0.904 | 0.624 | 5.15E-35 |

**Genes with avg_log2FC more than 0.5 have been shown in the table.**

| **Supplementary Table. 12 Marker genes of endothelial cells** | | | | | | |
| --- | --- | --- | --- | --- | --- | --- |
| **gene** | **p_val** | **avg_log2FC** | **pct.1** | **pct.2** | **p_val_adj** | **cluster** |
| Cd93 | 7.65E-76 | 1.376431817 | 0.913 | 0.33 | 1.57E-71 | 0 |
| Plvap | 1.45E-67 | 1.396206024 | 0.812 | 0.253 | 2.96E-63 | 0 |
| Sema3c | 3.10E-66 | 1.385739008 | 0.795 | 0.241 | 6.35E-62 | 0 |
| Ptprb | 2.62E-63 | 1.227423119 | 0.94 | 0.407 | 5.38E-59 | 0 |
| H2-Q6 | 3.63E-63 | 1.028609876 | 0.873 | 0.322 | 7.44E-59 | 0 |
| Kit | 3.84E-63 | 1.032091324 | 0.652 | 0.128 | 7.87E-59 | 0 |
| Npr3 | 2.57E-58 | 1.141937761 | 0.754 | 0.208 | 5.26E-54 | 0 |
| Tspan7 | 1.74E-53 | 1.083145384 | 0.895 | 0.43 | 3.56E-49 | 0 |
| Lpl | 1.78E-48 | 1.135067624 | 0.853 | 0.358 | 3.64E-44 | 0 |
| Itga1 | 6.21E-48 | 1.086537035 | 0.777 | 0.313 | 1.27E-43 | 0 |
| Gpihbp1 | 1.37E-47 | 1.044957992 | 0.839 | 0.365 | 2.80E-43 | 0 |
| Ier3 | 6.10E-47 | 1.200805758 | 0.926 | 0.609 | 1.25E-42 | 0 |
| Tek | 1.25E-44 | 1.028436999 | 0.556 | 0.15 | 2.55E-40 | 0 |
| Jun | 1.06E-38 | 1.052877327 | 0.973 | 0.791 | 2.16E-34 | 0 |
| Atf3 | 2.41E-35 | 1.232502705 | 0.828 | 0.452 | 4.93E-31 | 0 |
| H2-Ab1 | 1.03E-23 | 1.089739676 | 0.35 | 0.105 | 2.10E-19 | 0 |
| Cd74 | 5.50E-20 | 1.524204008 | 0.422 | 0.188 | 1.13E-15 | 0 |
| S100a9 | 9.33E-50 | 2.891260126 | 0.819 | 0.711 | 1.91E-45 | 1 |
| S100a8 | 3.20E-43 | 2.907361953 | 0.76 | 0.588 | 6.56E-39 | 1 |
| Mgp | 3.47E-32 | 1.9452038 | 0.713 | 0.584 | 7.11E-28 | 1 |
| Scgb1a1 | 7.94E-31 | 1.212165341 | 0.836 | 0.862 | 1.63E-26 | 1 |
| Saa3 | 2.80E-25 | 2.419358105 | 0.588 | 0.401 | 5.73E-21 | 1 |
| Gsn | 1.91E-20 | 2.086907525 | 0.541 | 0.403 | 3.91E-16 | 1 |
| Cfh | 2.74E-19 | 1.755735871 | 0.365 | 0.159 | 5.61E-15 | 1 |
| Thbs1 | 2.46E-16 | 1.882733594 | 0.38 | 0.205 | 5.04E-12 | 1 |
| Lyz2 | 4.98E-15 | 1.96725461 | 0.404 | 0.252 | 1.02E-10 | 1 |
| Ngp | 2.47E-14 | 2.045624716 | 0.33 | 0.171 | 5.06E-10 | 1 |
| Chil3 | 2.92E-12 | 1.767014451 | 0.357 | 0.217 | 5.98E-08 | 1 |
| Dcn | 1.33E-11 | 2.089753743 | 0.342 | 0.207 | 2.72E-07 | 1 |
| Apod | 2.30E-10 | 1.147956477 | 0.266 | 0.134 | 4.71E-06 | 1 |
| Cxcl2 | 4.14E-08 | 1.469635141 | 0.298 | 0.186 | 0.000848553 | 1 |
| Retnlg | 2.96E-07 | 1.989059796 | 0.266 | 0.169 | 0.006053808 | 1 |
| C3 | 1.62E-06 | 1.253679007 | 0.257 | 0.166 | 0.033198725 | 1 |
| S100a6 | 9.58E-06 | 1.282423516 | 0.354 | 0.287 | 0.196149008 | 1 |
| Sparcl1 | 1.03E-05 | 1.311401539 | 0.257 | 0.175 | 0.210516079 | 1 |
| Serpina3n | 1.92E-05 | 1.18537295 | 0.266 | 0.186 | 0.394115992 | 1 |
| Apoe | 0.0001905 | 1.128871544 | 0.307 | 0.25 | 1 | 1 |
| Fmo2 | 0.00332983 | 1.375912652 | 0.38 | 0.401 | 1 | 1 |
| Hbb-bs | 0.009713061 | 1.925544567 | 0.173 | 0.277 | 1 | 1 |
| Emp2 | 2.45E-114 | 3.849269533 | 0.851 | 0.162 | 5.02E-110 | 2 |
| Car4 | 1.75E-110 | 3.192813239 | 0.824 | 0.149 | 3.59E-106 | 2 |
| Ednrb | 1.46E-106 | 2.694370418 | 0.797 | 0.123 | 2.98E-102 | 2 |
| Igfbp7 | 3.00E-106 | 3.287613528 | 0.968 | 0.511 | 6.14E-102 | 2 |
| Enho | 6.63E-78 | 2.138521907 | 0.626 | 0.095 | 1.36E-73 | 2 |
| Hopx | 3.11E-74 | 2.242598257 | 0.865 | 0.361 | 6.37E-70 | 2 |
| Fibin | 2.11E-72 | 2.635970222 | 0.459 | 0.034 | 4.32E-68 | 2 |
| Pmp22 | 2.61E-71 | 2.195103176 | 0.851 | 0.376 | 5.35E-67 | 2 |
| AW112010 | 3.45E-66 | 2.98796807 | 0.644 | 0.141 | 7.06E-62 | 2 |
| Tbx2 | 2.11E-59 | 1.967753502 | 0.369 | 0.024 | 4.32E-55 | 2 |
| Prx | 5.69E-55 | 1.806529443 | 0.748 | 0.281 | 1.17E-50 | 2 |
| Bcam | 8.56E-54 | 1.757785034 | 0.631 | 0.175 | 1.75E-49 | 2 |
| Ccdc68 | 1.31E-53 | 1.743234228 | 0.392 | 0.04 | 2.69E-49 | 2 |
| Kitl | 1.60E-52 | 2.015474379 | 0.743 | 0.299 | 3.27E-48 | 2 |
| Lmo7 | 1.91E-50 | 1.371114557 | 0.428 | 0.059 | 3.90E-46 | 2 |
| Cyp4b1 | 3.00E-50 | 1.917722156 | 0.824 | 0.456 | 6.15E-46 | 2 |
| Apln | 1.76E-49 | 1.376844752 | 0.279 | 0.011 | 3.61E-45 | 2 |

| Icam2 | 2.31E-49 | 1.947629666 | 0.829 | 0.439 | 4.74E-45 | 2 |
| --- | --- | --- | --- | --- | --- | --- |
| Chst1 | 1.11E-48 | 1.245941097 | 0.302 | 0.017 | 2.28E-44 | 2 |
| Ptp4a3 | 1.14E-47 | 2.049979915 | 0.45 | 0.082 | 2.34E-43 | 2 |
| Clu | 4.98E-47 | 1.728693152 | 0.559 | 0.143 | 1.02E-42 | 2 |
| Nhlrc2 | 2.01E-46 | 1.709079725 | 0.514 | 0.119 | 4.12E-42 | 2 |
| Itga3 | 7.81E-46 | 1.734149828 | 0.55 | 0.143 | 1.60E-41 | 2 |
| Clic5 | 1.07E-43 | 1.533246444 | 0.851 | 0.566 | 2.18E-39 | 2 |
| Phlda3 | 2.05E-39 | 1.633479065 | 0.45 | 0.102 | 4.19E-35 | 2 |
| Tspan13 | 1.66E-38 | 1.777192026 | 0.676 | 0.299 | 3.39E-34 | 2 |
| Kdr | 8.75E-38 | 1.963817716 | 0.73 | 0.412 | 1.79E-33 | 2 |
| Hspb1 | 1.37E-37 | 1.859179746 | 0.856 | 0.515 | 2.82E-33 | 2 |
| App | 2.15E-35 | 1.327614107 | 0.824 | 0.596 | 4.41E-31 | 2 |
| Crip2 | 2.28E-35 | 1.302575218 | 0.833 | 0.55 | 4.67E-31 | 2 |
| Pcdh1 | 5.43E-35 | 1.479501636 | 0.604 | 0.235 | 1.11E-30 | 2 |
| Edil3 | 7.65E-35 | 1.288355062 | 0.387 | 0.077 | 1.57E-30 | 2 |
| Tuba1a | 1.56E-34 | 1.408970027 | 0.779 | 0.492 | 3.19E-30 | 2 |
| Ramp2 | 4.28E-34 | 1.150530826 | 0.941 | 0.734 | 8.78E-30 | 2 |
| Tbx3 | 4.53E-34 | 1.639588492 | 0.649 | 0.304 | 9.27E-30 | 2 |
| Trib2 | 1.66E-33 | 1.360653356 | 0.428 | 0.108 | 3.39E-29 | 2 |
| Rgs12 | 2.01E-33 | 1.433802057 | 0.608 | 0.254 | 4.12E-29 | 2 |
| Tspan8 | 4.28E-33 | 1.329704221 | 0.514 | 0.157 | 8.77E-29 | 2 |
| Anxa3 | 2.08E-32 | 1.234017452 | 0.743 | 0.419 | 4.25E-28 | 2 |
| Slc9a3r2 | 2.74E-32 | 1.249724952 | 0.802 | 0.467 | 5.62E-28 | 2 |
| Piezo2 | 3.35E-32 | 1.098037174 | 0.288 | 0.041 | 6.87E-28 | 2 |
| Tns3 | 1.06E-30 | 1.223070019 | 0.288 | 0.045 | 2.18E-26 | 2 |
| Tmem140 | 1.55E-30 | 1.400460746 | 0.577 | 0.237 | 3.17E-26 | 2 |
| Atp8a1 | 3.49E-30 | 1.401528933 | 0.554 | 0.221 | 7.15E-26 | 2 |
| Cd34 | 7.24E-30 | 1.255844248 | 0.707 | 0.372 | 1.48E-25 | 2 |
| Rasgrp2 | 3.11E-29 | 1.329569223 | 0.423 | 0.121 | 6.36E-25 | 2 |
| 4-Sep | 1.67E-27 | 1.536316201 | 0.374 | 0.101 | 3.42E-23 | 2 |
| Nectin3 | 5.87E-27 | 1.121463392 | 0.365 | 0.091 | 1.20E-22 | 2 |
| Fgfr3 | 8.47E-25 | 1.285726442 | 0.392 | 0.122 | 1.74E-20 | 2 |
| Stmn2 | 2.49E-24 | 1.661745362 | 0.432 | 0.155 | 5.09E-20 | 2 |
| Stard9 | 4.90E-24 | 1.103393741 | 0.505 | 0.194 | 1.00E-19 | 2 |
| Nipal3 | 7.14E-24 | 1.152401236 | 0.324 | 0.082 | 1.46E-19 | 2 |
| Agfg1 | 1.73E-23 | 1.274251805 | 0.486 | 0.2 | 3.55E-19 | 2 |
| Aard | 2.15E-23 | 1.049815501 | 0.279 | 0.059 | 4.41E-19 | 2 |
| Ehd4 | 6.70E-23 | 1.092255008 | 0.752 | 0.471 | 1.37E-18 | 2 |
| Glrx5 | 3.77E-22 | 1.257215764 | 0.432 | 0.166 | 7.72E-18 | 2 |
| Nrp1 | 4.12E-22 | 1.728937914 | 0.545 | 0.292 | 8.44E-18 | 2 |
| Zfhx3 | 5.15E-22 | 1.119858882 | 0.505 | 0.215 | 1.05E-17 | 2 |
| Grina | 8.52E-22 | 1.030446611 | 0.468 | 0.184 | 1.75E-17 | 2 |
| Krt80 | 8.57E-22 | 1.133124799 | 0.486 | 0.201 | 1.75E-17 | 2 |
| Rtl8a | 2.57E-21 | 1.254367 | 0.523 | 0.248 | 5.26E-17 | 2 |
| Ntn4 | 2.57E-21 | 1.015198314 | 0.324 | 0.089 | 5.26E-17 | 2 |
| Mgll | 2.69E-21 | 1.128598654 | 0.405 | 0.14 | 5.50E-17 | 2 |
| Impdh1 | 2.80E-21 | 1.277507922 | 0.428 | 0.17 | 5.74E-17 | 2 |
| Tmbim1 | 2.81E-21 | 1.056816109 | 0.486 | 0.213 | 5.75E-17 | 2 |
| Mxra7 | 1.76E-20 | 1.110913203 | 0.405 | 0.15 | 3.60E-16 | 2 |
| Cyth3 | 7.08E-20 | 1.172726719 | 0.653 | 0.432 | 1.45E-15 | 2 |
| Pllp | 1.97E-19 | 1.061990898 | 0.302 | 0.086 | 4.04E-15 | 2 |
| Mgst3 | 2.28E-19 | 1.099994766 | 0.261 | 0.065 | 4.66E-15 | 2 |
| Pdgfb | 5.70E-19 | 1.179627552 | 0.401 | 0.152 | 1.17E-14 | 2 |
| Scn7a | 5.74E-19 | 1.517137547 | 0.396 | 0.156 | 1.18E-14 | 2 |
| Smagp | 2.42E-18 | 1.073328418 | 0.608 | 0.367 | 4.95E-14 | 2 |
| Tppp3 | 3.69E-18 | 1.132480823 | 0.266 | 0.069 | 7.56E-14 | 2 |
| Agpat4 | 3.71E-18 | 1.118736724 | 0.514 | 0.259 | 7.59E-14 | 2 |
| Tspan18 | 3.14E-17 | 1.025944489 | 0.473 | 0.225 | 6.42E-13 | 2 |

| Nostrin | 9.90E-17 | 1.11635114 | 0.495 | 0.263 | 2.03E-12 | 2 |
| --- | --- | --- | --- | --- | --- | --- |
| Bin1 | 1.07E-16 | 1.080386198 | 0.392 | 0.163 | 2.18E-12 | 2 |
| Tmem204 | 2.44E-16 | 1.103861476 | 0.464 | 0.233 | 5.00E-12 | 2 |
| Acvrl1 | 2.78E-16 | 1.009520801 | 0.721 | 0.533 | 5.70E-12 | 2 |
| Adgrl2 | 1.44E-15 | 1.080352634 | 0.572 | 0.342 | 2.96E-11 | 2 |
| Arhgef3 | 4.47E-14 | 1.05052231 | 0.428 | 0.219 | 9.16E-10 | 2 |
| Tmcc2 | 1.35E-13 | 1.083030542 | 0.437 | 0.229 | 2.76E-09 | 2 |
| Rala | 1.58E-13 | 1.001709572 | 0.459 | 0.248 | 3.24E-09 | 2 |
| Ackr2 | 2.51E-13 | 1.020669393 | 0.41 | 0.201 | 5.14E-09 | 2 |
| Ccnd1 | 3.74E-12 | 1.041286259 | 0.333 | 0.148 | 7.65E-08 | 2 |
| Clec1a1 | 3.10E-11 | 1.066802278 | 0.446 | 0.254 | 6.36E-07 | 2 |
| Timp3 | 4.52E-10 | 1.290984996 | 0.788 | 0.704 | 9.26E-06 | 2 |
| Crip1 | 2.95E-08 | 1.03008161 | 0.505 | 0.346 | 0.000604943 | 2 |
| Mmrn1 | 1.46E-200 | 4.850185655 | 0.932 | 0.01 | 3.00E-196 | 3 |
| Reln | 9.37E-197 | 3.834429853 | 0.822 | 0.002 | 1.92E-192 | 3 |
| Sema3a | 6.06E-156 | 2.937333062 | 0.726 | 0.007 | 1.24E-151 | 3 |
| Prox1 | 3.75E-149 | 2.446129567 | 0.699 | 0.007 | 7.69E-145 | 3 |
| Maf | 8.50E-148 | 3.636690004 | 0.822 | 0.021 | 1.74E-143 | 3 |
| Ccl21a | 1.26E-143 | 6.173455877 | 0.767 | 0.017 | 2.59E-139 | 3 |
| Nts | 5.68E-133 | 4.379825324 | 0.63 | 0.007 | 1.16E-128 | 3 |
| Fgl2 | 7.38E-131 | 5.082603719 | 0.932 | 0.053 | 1.51E-126 | 3 |
| Rgs16 | 6.08E-126 | 2.9671701 | 0.644 | 0.01 | 1.25E-121 | 3 |
| Sema3d | 1.10E-113 | 2.31979847 | 0.589 | 0.009 | 2.26E-109 | 3 |
| Plce1 | 1.57E-106 | 1.753781515 | 0.493 | 0.004 | 3.21E-102 | 3 |
| Fxyd6 | 1.25E-93 | 2.310427716 | 0.589 | 0.02 | 2.56E-89 | 3 |
| Cp | 3.88E-92 | 3.203026463 | 0.863 | 0.076 | 7.94E-88 | 3 |
| Adamtsl1 | 2.45E-83 | 1.402108399 | 0.37 | 0.002 | 5.03E-79 | 3 |
| Klhl4 | 4.80E-80 | 1.453394609 | 0.37 | 0.003 | 9.83E-76 | 3 |
| Wipf3 | 6.98E-80 | 1.424960352 | 0.342 | 0.001 | 1.43E-75 | 3 |
| Sh3gl3 | 1.37E-79 | 1.213416157 | 0.342 | 0.001 | 2.80E-75 | 3 |
| Clca3a1 | 3.79E-77 | 2.617247837 | 0.658 | 0.046 | 7.76E-73 | 3 |
| Pdpn | 1.96E-74 | 1.723618858 | 0.589 | 0.032 | 4.02E-70 | 3 |
| Gm15655 | 5.21E-73 | 1.285784413 | 0.315 | 0.001 | 1.07E-68 | 3 |
| Gja1 | 2.55E-66 | 2.00133159 | 0.603 | 0.044 | 5.22E-62 | 3 |
| Igfbp5 | 2.00E-63 | 3.255791066 | 0.753 | 0.09 | 4.10E-59 | 3 |
| Nectin2 | 8.94E-61 | 1.747669976 | 0.479 | 0.027 | 1.83E-56 | 3 |
| Tshz2 | 2.95E-59 | 1.865763251 | 0.616 | 0.054 | 6.04E-55 | 3 |
| Gpm6a | 3.23E-58 | 1.316916717 | 0.356 | 0.01 | 6.61E-54 | 3 |
| Ctnnal1 | 3.10E-57 | 1.022943984 | 0.274 | 0.003 | 6.34E-53 | 3 |
| Thy1 | 5.00E-56 | 1.266499194 | 0.301 | 0.006 | 1.02E-51 | 3 |
| Itga9 | 8.36E-56 | 1.722304092 | 0.411 | 0.02 | 1.71E-51 | 3 |
| Dusp2 | 5.77E-55 | 1.829573604 | 0.466 | 0.029 | 1.18E-50 | 3 |
| Dtx1 | 6.18E-54 | 1.079433476 | 0.26 | 0.003 | 1.26E-49 | 3 |
| Scn1b | 8.74E-53 | 1.38043069 | 0.329 | 0.01 | 1.79E-48 | 3 |
| Tanc2 | 1.21E-52 | 1.353864855 | 0.356 | 0.014 | 2.48E-48 | 3 |
| Apold1 | 3.41E-52 | 2.625458786 | 0.74 | 0.118 | 6.99E-48 | 3 |
| Fcgrt | 8.87E-52 | 1.72969828 | 0.548 | 0.05 | 1.82E-47 | 3 |
| Nr2f2 | 5.57E-51 | 2.015460322 | 0.603 | 0.066 | 1.14E-46 | 3 |
| Pglyrp1 | 1.13E-50 | 2.758083176 | 0.534 | 0.051 | 2.32E-46 | 3 |
| Kctd17 | 3.36E-50 | 1.424242189 | 0.425 | 0.026 | 6.88E-46 | 3 |
| Flt4 | 2.39E-49 | 2.447063857 | 0.767 | 0.144 | 4.89E-45 | 3 |
| Slc45a3 | 9.00E-49 | 1.184445776 | 0.26 | 0.005 | 1.84E-44 | 3 |
| Lbp | 1.16E-48 | 2.615848379 | 0.753 | 0.139 | 2.38E-44 | 3 |
| Lama4 | 1.36E-48 | 1.697983481 | 0.425 | 0.028 | 2.78E-44 | 3 |
| Nxn | 2.36E-48 | 1.794037228 | 0.521 | 0.05 | 4.84E-44 | 3 |
| Adamts5 | 1.44E-47 | 1.987191385 | 0.37 | 0.02 | 2.95E-43 | 3 |
| Nrp2 | 4.03E-47 | 1.880985989 | 0.616 | 0.077 | 8.25E-43 | 3 |
| Ltbp4 | 1.19E-46 | 2.090474304 | 0.699 | 0.105 | 2.45E-42 | 3 |

| Rcan1 | 2.71E-44 | 2.048628897 | 0.589 | 0.079 | 5.54E-40 | 3 |
| --- | --- | --- | --- | --- | --- | --- |
| Palm | 6.12E-44 | 1.466028296 | 0.384 | 0.025 | 1.25E-39 | 3 |
| Fbln5 | 7.98E-42 | 1.262015631 | 0.342 | 0.02 | 1.64E-37 | 3 |
| Sned1 | 7.75E-39 | 1.331722197 | 0.342 | 0.022 | 1.59E-34 | 3 |
| Cdc42ep5 | 1.00E-38 | 1.101710328 | 0.301 | 0.016 | 2.05E-34 | 3 |
| Prelp | 9.09E-38 | 1.536455098 | 0.397 | 0.035 | 1.86E-33 | 3 |
| Dlg1 | 7.89E-37 | 2.200800006 | 0.575 | 0.095 | 1.62E-32 | 3 |
| Psen2 | 7.37E-36 | 1.355132029 | 0.384 | 0.035 | 1.51E-31 | 3 |
| Lcn2 | 9.75E-36 | 3.614627206 | 0.945 | 0.459 | 2.00E-31 | 3 |
| Cpne2 | 2.17E-35 | 1.257237656 | 0.356 | 0.029 | 4.44E-31 | 3 |
| Ccl2 | 3.60E-34 | 2.49529489 | 0.452 | 0.057 | 7.38E-30 | 3 |
| Rnd1 | 6.56E-33 | 1.596492804 | 0.438 | 0.053 | 1.34E-28 | 3 |
| Fam189a2 | 1.25E-32 | 1.025125305 | 0.274 | 0.017 | 2.55E-28 | 3 |
| Postn | 1.78E-32 | 1.286520159 | 0.301 | 0.022 | 3.65E-28 | 3 |
| Fth11 | 1.91E-32 | 1.966680133 | 1 | 0.969 | 3.92E-28 | 3 |
| Dab2 | 3.06E-32 | 1.467055953 | 0.589 | 0.106 | 6.26E-28 | 3 |
| Fabp4 | 3.06E-32 | 1.625950632 | 0.575 | 0.099 | 6.27E-28 | 3 |
| Adgrg3 | 6.57E-32 | 1.384711509 | 0.384 | 0.041 | 1.35E-27 | 3 |
| Serpine2 | 7.33E-32 | 1.377680536 | 0.425 | 0.051 | 1.50E-27 | 3 |
| Marcksl1 | 9.96E-32 | 1.505070473 | 0.507 | 0.08 | 2.04E-27 | 3 |
| Dennd4a | 8.25E-31 | 1.793652885 | 0.548 | 0.097 | 1.69E-26 | 3 |
| Ahnak | 4.72E-29 | 2.060752902 | 0.945 | 0.483 | 9.67E-25 | 3 |
| Rnf19b | 2.15E-27 | 1.853393654 | 0.479 | 0.082 | 4.41E-23 | 3 |
| Hsd3b7 | 3.62E-27 | 1.438152708 | 0.534 | 0.103 | 7.41E-23 | 3 |
| Cd631 | 4.66E-27 | 1.535677105 | 0.712 | 0.194 | 9.54E-23 | 3 |
| Stab11 | 6.79E-27 | 1.498204717 | 0.616 | 0.146 | 1.39E-22 | 3 |
| Ndrg1 | 9.43E-27 | 1.540172046 | 0.699 | 0.186 | 1.93E-22 | 3 |
| Stc1 | 3.81E-26 | 1.378557936 | 0.329 | 0.037 | 7.81E-22 | 3 |
| Tgm2 | 1.96E-25 | 1.713753878 | 0.822 | 0.314 | 4.01E-21 | 3 |
| Aebp1 | 4.15E-25 | 1.096577956 | 0.356 | 0.046 | 8.50E-21 | 3 |
| Csf3 | 7.40E-24 | 2.304945551 | 0.274 | 0.027 | 1.52E-19 | 3 |
| Sult1a1 | 9.75E-24 | 1.033980131 | 0.356 | 0.05 | 2.00E-19 | 3 |
| S100a61 | 1.93E-23 | 1.401055028 | 0.781 | 0.274 | 3.96E-19 | 3 |
| Slc10a6 | 2.96E-23 | 1.227724875 | 0.37 | 0.055 | 6.06E-19 | 3 |
| Abca1 | 6.01E-23 | 1.455961234 | 0.507 | 0.107 | 1.23E-18 | 3 |
| Ebf1 | 1.53E-22 | 1.12835522 | 0.356 | 0.052 | 3.13E-18 | 3 |
| Tmem173 | 7.34E-22 | 1.137463513 | 0.342 | 0.052 | 1.50E-17 | 3 |
| Adm | 5.62E-21 | 2.106065395 | 0.342 | 0.053 | 1.15E-16 | 3 |
| S100a11 | 5.37E-20 | 1.59815472 | 0.808 | 0.386 | 1.10E-15 | 3 |
| Casp4 | 1.52E-19 | 1.198236823 | 0.411 | 0.082 | 3.12E-15 | 3 |
| Slc39a14 | 6.62E-19 | 1.240295392 | 0.425 | 0.089 | 1.36E-14 | 3 |
| Sntb2 | 6.72E-19 | 1.454864844 | 0.534 | 0.151 | 1.38E-14 | 3 |
| Twsg1 | 7.53E-19 | 1.120414746 | 0.288 | 0.041 | 1.54E-14 | 3 |
| Bcr | 7.70E-19 | 1.54612939 | 0.411 | 0.092 | 1.58E-14 | 3 |
| Ppfibp1 | 2.39E-18 | 1.38670242 | 0.671 | 0.25 | 4.90E-14 | 3 |
| Mindy2 | 2.92E-18 | 1.43360438 | 0.493 | 0.128 | 5.97E-14 | 3 |
| Gpx1 | 7.30E-18 | 1.232498421 | 0.712 | 0.27 | 1.49E-13 | 3 |
| Gng11 | 1.40E-17 | 1.884042302 | 0.74 | 0.355 | 2.88E-13 | 3 |
| Mmrn2 | 1.43E-17 | 1.041739067 | 0.411 | 0.091 | 2.94E-13 | 3 |
| Tmem64 | 2.18E-17 | 1.10835383 | 0.288 | 0.045 | 4.47E-13 | 3 |
| Comt | 3.03E-17 | 1.229893857 | 0.384 | 0.082 | 6.20E-13 | 3 |
| Timp21 | 3.50E-17 | 1.401533718 | 0.781 | 0.377 | 7.17E-13 | 3 |
| Nudt4 | 8.80E-17 | 1.256743059 | 0.452 | 0.117 | 1.80E-12 | 3 |
| Cavin3 | 2.73E-16 | 1.407080475 | 0.493 | 0.145 | 5.58E-12 | 3 |
| Nupr1 | 1.61E-15 | 1.339927895 | 0.37 | 0.081 | 3.30E-11 | 3 |
| Pard6g1 | 4.23E-15 | 1.179818537 | 0.493 | 0.147 | 8.66E-11 | 3 |
| Ephx1 | 4.44E-15 | 1.052691185 | 0.288 | 0.053 | 9.10E-11 | 3 |
| Errfi1 | 4.57E-15 | 1.574275284 | 0.534 | 0.178 | 9.35E-11 | 3 |

| Ctsb | 5.02E-15 | 1.144770014 | 0.726 | 0.312 | 1.03E-10 | 3 |
| --- | --- | --- | --- | --- | --- | --- |
| Nfia | 7.99E-15 | 1.101848547 | 0.452 | 0.129 | 1.64E-10 | 3 |
| Cd9 | 1.03E-14 | 1.130043179 | 0.932 | 0.688 | 2.10E-10 | 3 |
| Ptpn14 | 1.35E-13 | 1.092670941 | 0.315 | 0.069 | 2.76E-09 | 3 |
| Serping1 | 2.17E-13 | 1.078368855 | 0.452 | 0.13 | 4.44E-09 | 3 |
| S100a10 | 3.40E-13 | 1.351967873 | 0.767 | 0.471 | 6.96E-09 | 3 |
| Ugcg | 5.66E-13 | 1.331800953 | 0.603 | 0.257 | 1.16E-08 | 3 |
| Trf | 1.45E-12 | 1.029689395 | 0.301 | 0.067 | 2.97E-08 | 3 |
| Ifi27l2a | 1.57E-12 | 2.287437668 | 0.452 | 0.152 | 3.22E-08 | 3 |
| Serpina3n1 | 1.90E-12 | 1.481843956 | 0.521 | 0.189 | 3.89E-08 | 3 |
| Emcn | 2.40E-12 | 1.196323438 | 0.507 | 0.182 | 4.91E-08 | 3 |
| Sh3glb2 | 4.68E-12 | 1.122057523 | 0.274 | 0.058 | 9.59E-08 | 3 |
| Yes1 | 7.96E-12 | 1.410050078 | 0.479 | 0.176 | 1.63E-07 | 3 |
| Gapdh | 2.49E-11 | 1.063467994 | 0.781 | 0.517 | 5.11E-07 | 3 |
| Trib1 | 2.57E-11 | 1.19469164 | 0.575 | 0.24 | 5.25E-07 | 3 |
| Egr11 | 2.63E-11 | 1.27965315 | 0.836 | 0.606 | 5.38E-07 | 3 |
| Lrg1 | 5.61E-11 | 1.032221711 | 0.644 | 0.294 | 1.15E-06 | 3 |
| Junb1 | 1.22E-10 | 1.046673768 | 0.945 | 0.76 | 2.50E-06 | 3 |
| Ifitm2 | 1.38E-10 | 1.069211575 | 0.904 | 0.836 | 2.82E-06 | 3 |
| Vim1 | 1.40E-10 | 1.108128951 | 0.822 | 0.572 | 2.87E-06 | 3 |
| Ctnna1 | 2.89E-09 | 1.153629584 | 0.795 | 0.583 | 5.92E-05 | 3 |
| Fosb1 | 3.33E-09 | 1.353681789 | 0.822 | 0.568 | 6.83E-05 | 3 |
| Gls1 | 4.91E-09 | 1.114307039 | 0.616 | 0.31 | 0.00010061 | 3 |
| Rps6ka3 | 5.79E-09 | 1.009354266 | 0.425 | 0.162 | 0.000118526 | 3 |
| Nfat5 | 6.78E-09 | 1.396783035 | 0.644 | 0.391 | 0.000138918 | 3 |
| Cxcl1 | 9.02E-09 | 1.349888961 | 0.37 | 0.129 | 0.000184681 | 3 |
| Rassf9 | 1.26E-08 | 1.033579417 | 0.397 | 0.146 | 0.000258934 | 3 |
| Tm4sf11 | 1.27E-08 | 1.103893241 | 0.726 | 0.448 | 0.000260937 | 3 |
| Ntn11 | 1.88E-08 | 1.015633094 | 0.438 | 0.184 | 0.00038426 | 3 |
| Fos1 | 5.59E-08 | 1.054450723 | 0.863 | 0.716 | 0.001144061 | 3 |
| Ccn1 | 1.10E-07 | 1.448119399 | 0.603 | 0.352 | 0.002251475 | 3 |
| Nfkbiz | 1.45E-07 | 1.07696973 | 0.521 | 0.257 | 0.00296462 | 3 |
| Neat1 | 5.10E-06 | 1.306042824 | 0.699 | 0.542 | 0.10437425 | 3 |
| Apod1 | 7.27E-05 | 1.864625074 | 0.342 | 0.162 | 1 | 3 |
| Adamts1 | 7.70E-05 | 1.25243171 | 0.699 | 0.501 | 1 | 3 |
| Cxcl21 | 0.000181757 | 1.117788465 | 0.397 | 0.208 | 1 | 3 |
| Cytl1 | 2.30E-120 | 3.615551039 | 0.688 | 0.008 | 4.72E-116 | 4 |
| Tmem45a | 6.55E-68 | 1.368041598 | 0.406 | 0.005 | 1.34E-63 | 4 |
| Car8 | 1.05E-61 | 1.484260457 | 0.406 | 0.007 | 2.15E-57 | 4 |
| AU021092 | 1.32E-59 | 1.109687317 | 0.312 | 0.003 | 2.71E-55 | 4 |
| Vcam1 | 2.60E-52 | 3.18304949 | 0.719 | 0.047 | 5.32E-48 | 4 |
| Enpp6 | 1.17E-49 | 1.018233239 | 0.25 | 0.002 | 2.39E-45 | 4 |
| Myof | 2.73E-47 | 1.607369196 | 0.406 | 0.013 | 5.59E-43 | 4 |
| Cpe | 1.49E-45 | 2.297093704 | 0.531 | 0.028 | 3.05E-41 | 4 |
| Vwf | 5.18E-43 | 3.883778124 | 0.969 | 0.151 | 1.06E-38 | 4 |
| Slc6a2 | 1.19E-42 | 2.984783956 | 0.406 | 0.015 | 2.43E-38 | 4 |
| Cdh13 | 6.18E-39 | 1.662304955 | 0.375 | 0.014 | 1.27E-34 | 4 |
| Adgrg6 | 2.15E-38 | 1.949972287 | 0.438 | 0.022 | 4.40E-34 | 4 |
| Bst1 | 3.96E-38 | 1.795372102 | 0.469 | 0.026 | 8.10E-34 | 4 |
| Sorbs2 | 8.16E-34 | 1.276182433 | 0.344 | 0.014 | 1.67E-29 | 4 |
| Selp | 1.77E-31 | 2.807660586 | 0.562 | 0.05 | 3.63E-27 | 4 |
| Fbln51 | 1.39E-30 | 1.498535186 | 0.438 | 0.029 | 2.84E-26 | 4 |
| Bmx | 6.21E-27 | 1.377102579 | 0.344 | 0.02 | 1.27E-22 | 4 |
| Sele | 6.87E-27 | 2.36362318 | 0.375 | 0.024 | 1.41E-22 | 4 |
| Ctsh | 2.21E-26 | 1.498714733 | 0.406 | 0.031 | 4.53E-22 | 4 |
| Ptgs2 | 1.30E-25 | 2.578549573 | 0.531 | 0.055 | 2.66E-21 | 4 |
| Ptprr | 3.04E-24 | 1.013387215 | 0.25 | 0.011 | 6.23E-20 | 4 |
| Sulf1 | 4.75E-24 | 1.726940786 | 0.344 | 0.023 | 9.73E-20 | 4 |

| Fam107a | 1.89E-21 | 1.229511721 | 0.469 | 0.05 | 3.88E-17 | 4 |
| --- | --- | --- | --- | --- | --- | --- |
| Mecom | 3.54E-19 | 1.250287381 | 0.406 | 0.044 | 7.25E-15 | 4 |
| Eln1 | 9.87E-19 | 1.663333159 | 0.562 | 0.087 | 2.02E-14 | 4 |
| Samd5 | 2.90E-18 | 2.035458168 | 0.656 | 0.127 | 5.93E-14 | 4 |
| Emp3 | 1.44E-17 | 1.577467147 | 0.594 | 0.102 | 2.94E-13 | 4 |
| Jam2 | 3.37E-17 | 1.49512153 | 0.781 | 0.187 | 6.90E-13 | 4 |
| Mustn1 | 4.33E-16 | 1.051676084 | 0.375 | 0.045 | 8.87E-12 | 4 |
| Fstl11 | 4.90E-16 | 1.506129305 | 0.562 | 0.099 | 1.00E-11 | 4 |
| Procr | 2.25E-15 | 1.489728508 | 0.406 | 0.055 | 4.60E-11 | 4 |
| Ch25h | 4.55E-14 | 1.463347635 | 0.344 | 0.044 | 9.33E-10 | 4 |
| Bmp4 | 1.03E-13 | 1.187800687 | 0.406 | 0.062 | 2.11E-09 | 4 |
| Ptprj | 2.25E-12 | 1.578080868 | 0.5 | 0.103 | 4.61E-08 | 4 |
| Fbln2 | 2.63E-12 | 1.224486686 | 0.25 | 0.027 | 5.39E-08 | 4 |
| Mmrn21 | 1.26E-11 | 1.539610775 | 0.469 | 0.101 | 2.59E-07 | 4 |
| Cd81 | 2.46E-11 | 1.261472333 | 0.531 | 0.129 | 5.03E-07 | 4 |
| Nr2f21 | 1.25E-10 | 1.2889789 | 0.438 | 0.09 | 2.57E-06 | 4 |
| Plac8 | 1.59E-10 | 1.602901062 | 0.625 | 0.186 | 3.25E-06 | 4 |
| Cemip21 | 3.56E-10 | 1.343961379 | 0.812 | 0.334 | 7.28E-06 | 4 |
| Itgb3 | 3.78E-10 | 1.02365248 | 0.281 | 0.041 | 7.75E-06 | 4 |
| Tm4sf12 | 3.93E-10 | 1.660343197 | 0.938 | 0.452 | 8.05E-06 | 4 |
| Prss231 | 5.25E-09 | 2.031988592 | 0.438 | 0.116 | 0.000107557 | 4 |
| Ltbp41 | 7.53E-09 | 1.745902708 | 0.469 | 0.133 | 0.000154197 | 4 |
| Nucb2 | 1.22E-08 | 1.228182256 | 0.344 | 0.071 | 0.000249654 | 4 |
| Synpo | 2.37E-08 | 1.220185302 | 0.344 | 0.072 | 0.000485579 | 4 |
| Insr | 2.45E-08 | 1.53144798 | 0.344 | 0.071 | 0.000500769 | 4 |
| Adam15 | 2.63E-08 | 1.317923701 | 0.656 | 0.248 | 0.000537858 | 4 |
| Serpinb6b | 7.80E-08 | 1.145006696 | 0.344 | 0.078 | 0.001597884 | 4 |
| Fxyd5 | 9.24E-08 | 1.359347271 | 0.75 | 0.381 | 0.001891502 | 4 |
| Fam3c | 2.32E-07 | 1.276054105 | 0.438 | 0.124 | 0.004749065 | 4 |
| Tinagl11 | 3.73E-07 | 1.484575773 | 0.688 | 0.335 | 0.00764011 | 4 |
| Rps31 | 5.25E-07 | 1.114693698 | 0.969 | 0.766 | 0.010755947 | 4 |
| Rpl36a | 1.12E-06 | 1.099545458 | 0.938 | 0.578 | 0.022977242 | 4 |
| Ifitm21 | 1.16E-06 | 1.051539338 | 0.969 | 0.837 | 0.023729607 | 4 |
| Bgn | 1.16E-06 | 1.252365209 | 0.5 | 0.174 | 0.023856945 | 4 |
| Ahnak1 | 1.84E-06 | 1.137491859 | 0.812 | 0.504 | 0.037597576 | 4 |
| Rps12 | 1.86E-06 | 1.085802504 | 0.969 | 0.833 | 0.038141431 | 4 |
| Atp13a31 | 1.93E-06 | 1.539130002 | 0.406 | 0.123 | 0.03962769 | 4 |
| Atp1b3 | 1.94E-06 | 1.109179892 | 0.719 | 0.363 | 0.039647509 | 4 |
| Gja4 | 2.13E-06 | 1.19946833 | 0.344 | 0.094 | 0.043697534 | 4 |
| Apbb2 | 2.17E-06 | 1.356479748 | 0.438 | 0.148 | 0.044416661 | 4 |
| Rfk | 2.30E-06 | 1.045100429 | 0.375 | 0.112 | 0.047077095 | 4 |
| Palmd1 | 2.39E-06 | 1.032476483 | 0.5 | 0.171 | 0.048984597 | 4 |
| Rps28 | 3.31E-06 | 1.059874673 | 0.938 | 0.647 | 0.067745119 | 4 |
| Icam1 | 4.54E-06 | 1.332834786 | 0.781 | 0.475 | 0.092994779 | 4 |
| Rps251 | 5.74E-06 | 1.050643809 | 0.938 | 0.714 | 0.117537967 | 4 |
| Csrp21 | 2.03E-05 | 1.254947183 | 0.5 | 0.223 | 0.415370385 | 4 |
| Igfbp41 | 6.57E-05 | 1.717853791 | 0.719 | 0.463 | 1 | 4 |
| Mgp1 | 8.39E-05 | 2.187222383 | 0.812 | 0.618 | 1 | 4 |
| Rps8 | 0.000227339 | 1.07219642 | 0.969 | 0.865 | 1 | 4 |
| Irak3 | 0.000332366 | 1.069471333 | 0.25 | 0.074 | 1 | 4 |
| Jag1 | 0.000390809 | 1.1290511 | 0.281 | 0.099 | 1 | 4 |
| Rpl30 | 0.000552725 | 1.018802082 | 0.906 | 0.775 | 1 | 4 |
| 11-Sep | 0.000577254 | 1.037845579 | 0.406 | 0.17 | 1 | 4 |
| Uba52 | 0.000703208 | 1.108957003 | 0.75 | 0.448 | 1 | 4 |
| Abi3bp1 | 0.000770112 | 1.158995308 | 0.312 | 0.124 | 1 | 4 |
| Rps152 | 0.000874623 | 1.026181293 | 0.844 | 0.726 | 1 | 4 |
| Rpl37a | 0.001053888 | 1.086842494 | 0.938 | 0.784 | 1 | 4 |
| Rps211 | 0.001214776 | 1.076582974 | 0.906 | 0.698 | 1 | 4 |

| Lrg11 | 0.001228724 | 1.449711832 | 0.531 | 0.31 | 1 | 4 |
| --- | --- | --- | --- | --- | --- | --- |
| Igfbp51 | 0.002062156 | 2.403886629 | 0.312 | 0.127 | 1 | 4 |
| Rpl31 | 0.002357654 | 1.066350315 | 0.75 | 0.528 | 1 | 4 |
| Tsc22d1 | 0.002728153 | 1.01332451 | 0.562 | 0.338 | 1 | 4 |
| Ccn2 | 0.002814587 | 1.077493547 | 0.25 | 0.095 | 1 | 4 |
| Rflnb | 1.73E-59 | 2.342288901 | 0.75 | 0.034 | 3.55E-55 | 5 |
| Pde2a | 7.60E-52 | 1.696664585 | 0.542 | 0.018 | 1.56E-47 | 5 |
| Thrsp | 3.42E-47 | 1.179081163 | 0.333 | 0.005 | 7.01E-43 | 5 |
| Tcf15 | 1.19E-46 | 1.84670675 | 0.375 | 0.008 | 2.43E-42 | 5 |
| Fabp41 | 1.56E-45 | 5.41117304 | 1 | 0.111 | 3.21E-41 | 5 |
| Sorbs21 | 2.69E-45 | 1.19867174 | 0.458 | 0.014 | 5.50E-41 | 5 |
| Fabp5 | 9.89E-39 | 2.155091865 | 0.542 | 0.028 | 2.03E-34 | 5 |
| Rgcc | 5.34E-38 | 3.030965885 | 0.667 | 0.047 | 1.09E-33 | 5 |
| Tshz22 | 1.40E-37 | 1.894491698 | 0.833 | 0.074 | 2.86E-33 | 5 |
| Meox2 | 3.79E-36 | 1.422407483 | 0.292 | 0.006 | 7.76E-32 | 5 |
| Ablim3 | 8.04E-34 | 1.067741533 | 0.292 | 0.007 | 1.65E-29 | 5 |
| Rbp7 | 1.15E-32 | 1.898524626 | 0.333 | 0.011 | 2.35E-28 | 5 |
| Col15a1 | 4.02E-31 | 1.22420912 | 0.333 | 0.012 | 8.23E-27 | 5 |
| Dnm3 | 1.94E-29 | 1.511717062 | 0.375 | 0.017 | 3.98E-25 | 5 |
| Hspg2 | 2.22E-29 | 1.506496194 | 0.458 | 0.027 | 4.55E-25 | 5 |
| Cd300lg | 5.80E-27 | 1.733996923 | 0.583 | 0.053 | 1.19E-22 | 5 |
| Ebf11 | 4.08E-23 | 1.583110003 | 0.583 | 0.061 | 8.37E-19 | 5 |
| Igfbp3 | 1.16E-20 | 3.340908604 | 0.583 | 0.071 | 2.38E-16 | 5 |
| Cdc42ep3 | 3.71E-18 | 1.375954864 | 0.5 | 0.056 | 7.60E-14 | 5 |
| Galnt15 | 3.33E-17 | 1.357838618 | 0.458 | 0.049 | 6.82E-13 | 5 |
| Ifit2 | 5.19E-16 | 1.488714474 | 0.375 | 0.037 | 1.06E-11 | 5 |
| Ctnnbip1 | 2.03E-15 | 1.391181537 | 0.5 | 0.066 | 4.16E-11 | 5 |
| Emcn1 | 2.13E-15 | 1.801334484 | 0.792 | 0.19 | 4.35E-11 | 5 |
| Ncoa7 | 2.46E-14 | 1.62503002 | 0.458 | 0.062 | 5.03E-10 | 5 |
| Abcg22 | 6.93E-14 | 1.741558115 | 0.625 | 0.123 | 1.42E-09 | 5 |
| Jam21 | 1.63E-13 | 1.408638665 | 0.792 | 0.191 | 3.34E-09 | 5 |
| Mgll2 | 1.79E-13 | 1.764268264 | 0.75 | 0.18 | 3.67E-09 | 5 |
| Mmrn22 | 2.54E-12 | 1.17151712 | 0.542 | 0.102 | 5.19E-08 | 5 |
| Shroom4 | 5.53E-12 | 1.036435162 | 0.417 | 0.06 | 1.13E-07 | 5 |
| Lpar6 | 1.06E-10 | 1.259543733 | 0.5 | 0.1 | 2.18E-06 | 5 |
| Sparcl11 | 5.23E-10 | 1.055035742 | 0.708 | 0.189 | 1.07E-05 | 5 |
| Apol10b | 5.57E-10 | 1.261234912 | 0.292 | 0.037 | 1.14E-05 | 5 |
| Tcim1 | 1.59E-09 | 1.666582215 | 0.667 | 0.19 | 3.26E-05 | 5 |
| Aplnr | 3.10E-09 | 1.129770697 | 0.292 | 0.04 | 6.35E-05 | 5 |
| Rsad2 | 5.71E-09 | 1.583918514 | 0.333 | 0.053 | 0.000116938 | 5 |
| Fryl | 6.26E-09 | 1.145244674 | 0.5 | 0.117 | 0.000128127 | 5 |
| Hspb11 | 2.37E-08 | 1.515637849 | 0.958 | 0.573 | 0.000485819 | 5 |
| Cd811 | 2.56E-08 | 1.702360628 | 0.5 | 0.132 | 0.000524593 | 5 |
| Rps6ka32 | 4.54E-08 | 1.185534955 | 0.583 | 0.17 | 0.000929457 | 5 |
| Cxcl121 | 5.76E-08 | 1.038001474 | 0.583 | 0.158 | 0.001179078 | 5 |
| Tinagl12 | 7.41E-08 | 1.227856189 | 0.792 | 0.335 | 0.001518006 | 5 |
| Plpp31 | 7.97E-08 | 1.256298419 | 0.75 | 0.275 | 0.001632322 | 5 |
| Atp8b1 | 9.54E-08 | 1.318216454 | 0.417 | 0.093 | 0.001953991 | 5 |
| Gbp51 | 1.54E-07 | 1.202701309 | 0.5 | 0.132 | 0.003156303 | 5 |
| Sparc1 | 2.53E-07 | 1.062651483 | 0.917 | 0.461 | 0.005188181 | 5 |
| Ier5l1 | 2.74E-07 | 1.077018231 | 0.792 | 0.283 | 0.005611483 | 5 |
| Nudt42 | 3.71E-07 | 1.080793825 | 0.5 | 0.131 | 0.007590853 | 5 |
| Ifit3b | 4.01E-07 | 1.410653843 | 0.333 | 0.064 | 0.008215535 | 5 |
| Grrp11 | 2.39E-06 | 1.357332525 | 0.583 | 0.219 | 0.048871847 | 5 |
| Gtpbp4 | 2.44E-06 | 1.0002802 | 0.5 | 0.147 | 0.049980381 | 5 |
| Apoe1 | 3.02E-06 | 1.238642587 | 0.667 | 0.259 | 0.06177767 | 5 |
| Fli1 | 4.70E-06 | 1.05663426 | 0.625 | 0.235 | 0.096179344 | 5 |
| Scgb3a1 | 6.07E-06 | 1.189330663 | 0.292 | 0.06 | 0.124349246 | 5 |

| Sp1001 | 8.50E-06 | 1.132665531 | 0.5 | 0.164 | 0.174164073 | 5 |
| --- | --- | --- | --- | --- | --- | --- |
| Prss232 | 9.98E-06 | 1.066477445 | 0.417 | 0.119 | 0.2044045 | 5 |
| Sox171 | 1.09E-05 | 1.140636568 | 0.542 | 0.199 | 0.222891123 | 5 |
| Cd362 | 1.24E-05 | 1.04580966 | 0.958 | 0.704 | 0.253361595 | 5 |
| Btg12 | 1.44E-05 | 1.372244661 | 0.833 | 0.474 | 0.294904295 | 5 |
| Mndal1 | 1.66E-05 | 1.30539765 | 0.458 | 0.149 | 0.339120965 | 5 |
| Irf7 | 2.08E-05 | 1.118990094 | 0.417 | 0.132 | 0.425041857 | 5 |
| Slfn52 | 2.44E-05 | 1.142589507 | 0.875 | 0.5 | 0.499875277 | 5 |
| Gbp21 | 2.93E-05 | 1.552498308 | 0.625 | 0.25 | 0.601116404 | 5 |
| Slc39a141 | 4.00E-05 | 1.13467178 | 0.375 | 0.105 | 0.820113757 | 5 |
| Mast41 | 5.30E-05 | 1.218064696 | 0.667 | 0.301 | 1 | 5 |
| Rnd12 | 5.75E-05 | 1.075634953 | 0.292 | 0.073 | 1 | 5 |
| Pdzd21 | 6.84E-05 | 1.357786561 | 0.542 | 0.229 | 1 | 5 |
| Ifi2031 | 0.000109926 | 1.30066983 | 0.417 | 0.143 | 1 | 5 |
| Adamts9 | 0.000112607 | 1.394794553 | 0.667 | 0.325 | 1 | 5 |
| Hspa1a1 | 0.000113576 | 1.376869678 | 0.708 | 0.352 | 1 | 5 |
| Itga61 | 0.000191864 | 1.084755882 | 0.625 | 0.312 | 1 | 5 |
| Egr12 | 0.00033048 | 1.018443007 | 0.833 | 0.616 | 1 | 5 |
| Adamts12 | 0.000441873 | 1.021984396 | 0.792 | 0.508 | 1 | 5 |
| Pkp41 | 0.000469986 | 1.138903481 | 0.542 | 0.244 | 1 | 5 |
| Ifit11 | 0.001046431 | 1.269328457 | 0.417 | 0.182 | 1 | 5 |
| Gbp71 | 0.001678052 | 1.368859917 | 0.542 | 0.303 | 1 | 5 |
| Iigp11 | 0.003581436 | 1.222347996 | 0.625 | 0.397 | 1 | 5 |
| Hk1 | 0.004550138 | 1.091280216 | 0.292 | 0.117 | 1 | 5 |

**Genes with avg_log2FC more than 1 have been shown in the table.**

| **Supplementary Table. 13** | | | | | | | |
| --- | --- | --- | --- | --- | --- | --- | --- |
| **Univariate logistic regression** | | | | | | | |
| **Gene** | **Odds.Ratio** | **X95.CI** | **P.value** | **Gene** | **Odds.Ratio** | **X95.CI** | **P.value** |
| ABCA13 | 1.14 | 1.03-1.27 | 0.012 | NDUFA13 | 1.17 | 0.99-1.39 | 0.067 |
| ABHD5 | 1.2 | 0.99-1.45 | 0.067 | NDUFA4 | 1.12 | 0.96-1.32 | 0.155 |
| ABRACL | 1.01 | 0.83-1.24 | 0.892 | NDUFA8 | 1.03 | 0.85-1.25 | 0.757 |
| ACADL | 0.33 | 0.02-3.48 | 0.367 | NDUFB10 | 1.02 | 0.83-1.27 | 0.84 |
| ACAP1 | 1.3 | 1.09-1.56 | 0.004 | NDUFB11 | 0.96 | 0.79-1.16 | 0.688 |
| ACTN1 | 1.22 | 1.03-1.46 | 0.025 | NDUFB5 | 0.91 | 0.73-1.12 | 0.371 |
| ACTR3 | 1.16 | 0.93-1.44 | 0.188 | NDUFB6 | 1 | 0.8-1.25 | 0.983 |
| ACVRL1 | 1.2 | 1.03-1.42 | 0.024 | NDUFB7 | 1.04 | 0.9-1.19 | 0.609 |
| ADAM8 | 1.18 | 1.02-1.37 | 0.024 | NDUFB8 | 0.95 | 0.79-1.15 | 0.622 |
| ADD1 | 1.3 | 1.03-1.67 | 0.03 | NDUFC1 | 1.1 | 0.89-1.37 | 0.368 |
| ADD3 | 1.08 | 0.92-1.27 | 0.335 | NDUFS4 | 1.01 | 0.85-1.2 | 0.885 |
| ADPGK | 1.05 | 0.85-1.29 | 0.671 | NDUFS6 | 1.03 | 0.84-1.25 | 0.8 |
| AGPAT2 | 1.18 | 1.01-1.38 | 0.044 | NDUFV2 | 1.16 | 0.96-1.41 | 0.119 |
| AK2 | 0.89 | 0.72-1.11 | 0.31 | NDUFV3 | 0.98 | 0.78-1.24 | 0.868 |
| AKR1A1 | 0.96 | 0.8-1.15 | 0.664 | NFE2 | 1.33 | 1.12-1.6 | 0.002 |
| ALAS1 | 1.25 | 1.01-1.56 | 0.042 | NFIC | 1.11 | 0.93-1.33 | 0.262 |
| ALDH2 | 1.05 | 0.87-1.27 | 0.59 | NFU1 | 1.13 | 0.92-1.4 | 0.239 |
| ALOX5 | 1.32 | 1.11-1.56 | 0.001 | NHSL2 | 0.98 | 0.82-1.17 | 0.856 |
| ALOX5AP | 1.26 | 1.07-1.5 | 0.008 | NIN | 1.16 | 0.97-1.39 | 0.098 |
| ANAPC13 | 0.82 | 0.65-1.02 | 0.076 | NMT1 | 1.11 | 0.89-1.39 | 0.362 |
| ANKRD13A | 1.03 | 0.84-1.27 | 0.752 | NOTCH1 | 1.12 | 0.96-1.32 | 0.139 |
| ANO10 | 1.44 | 1.16-1.79 | 0.001 | NPEPPS | 1.18 | 0.95-1.46 | 0.134 |
| ANXA1 | 1.23 | 1.02-1.5 | 0.029 | NRAS | 1.05 | 0.89-1.23 | 0.596 |
| ANXA11 | 1.22 | 1-1.5 | 0.057 | NSA2 | 0.86 | 0.71-1.05 | 0.144 |
| ANXA3 | 1.31 | 1.16-1.5 | <0.001 | NUCB2 | 1.07 | 0.9-1.27 | 0.447 |
| ANXA5 | 1.09 | 0.86-1.39 | 0.452 | OAZ1 | 1.25 | 1-1.57 | 0.053 |
| ANXA7 | 1.19 | 0.92-1.54 | 0.177 | OLFML2B | 1.07 | 0.93-1.24 | 0.345 |
| AP2A2 | 1.12 | 0.91-1.38 | 0.276 | P4HB | 1.16 | 0.97-1.4 | 0.115 |
| AP2S1 | 1.08 | 0.86-1.35 | 0.509 | PACSIN2 | 1.24 | 1.02-1.5 | 0.032 |
| AP3S1 | 1.24 | 1.01-1.52 | 0.041 | PADI4 | 1.4 | 1.19-1.65 | <0.001 |
| APP | 1.18 | 0.98-1.43 | 0.089 | PARK7 | 1.04 | 0.82-1.31 | 0.769 |
| APPBP2 | 0.98 | 0.8-1.19 | 0.825 | PDCD6 | 1.05 | 0.85-1.31 | 0.638 |
| APRT | 0.93 | 0.8-1.08 | 0.352 | PDCL3 | 0.97 | 0.81-1.17 | 0.758 |
| ARCN1 | 1.07 | 0.85-1.36 | 0.557 | PDIA3 | 0.99 | 0.8-1.24 | 0.96 |
| ARF5 | 1.09 | 0.88-1.34 | 0.435 | PDIA6 | 1.06 | 0.88-1.28 | 0.523 |
| ARFGEF1 | 1.11 | 0.92-1.33 | 0.271 | PDZD8 | 1.24 | 1.06-1.47 | 0.008 |
| ARHGAP30 | 1.16 | 0.93-1.46 | 0.188 | PFN1 | 1.14 | 0.93-1.39 | 0.197 |
| ARHGDIB | 1.14 | 0.91-1.43 | 0.251 | PGAM1 | 1.19 | 0.98-1.45 | 0.079 |
| ARHGEF1 | 1.12 | 0.94-1.33 | 0.215 | PGD | 1.49 | 1.24-1.81 | <0.001 |
| ARL2BP | 0.95 | 0.77-1.17 | 0.642 | PGK1 | 1.39 | 1.1-1.76 | 0.006 |
| ARPC2 | 1.21 | 0.95-1.54 | 0.13 | PGLS | 1.06 | 0.9-1.26 | 0.478 |
| ARPC4 | 1.15 | 0.92-1.44 | 0.208 | PGLYRP1 | 1.27 | 1.13-1.44 | <0.001 |
| ARPC5 | 1.22 | 0.99-1.52 | 0.061 | PHF20L1 | 1.15 | 0.94-1.4 | 0.185 |
| ARRB2 | 1.26 | 1.03-1.55 | 0.023 | PITPNA | 0.99 | 0.79-1.23 | 0.897 |
| ASNSD1 | 1.07 | 0.89-1.28 | 0.469 | PLBD1 | 1.37 | 1.13-1.68 | 0.002 |
| ATF4 | 1.13 | 0.92-1.38 | 0.241 | PLEC | 1.28 | 1.09-1.53 | 0.004 |
| ATP8B4 | 1.15 | 0.98-1.34 | 0.082 | PLP2 | 1.32 | 1.1-1.6 | 0.004 |
| ATRN | 1 | 0.85-1.19 | 0.97 | PLSCR1 | 1.19 | 1.01-1.4 | 0.045 |
| ATRX | 1.02 | 0.84-1.24 | 0.849 | PLXNC1 | 1.16 | 1-1.36 | 0.053 |
| ATXN10 | 0.86 | 0.71-1.05 | 0.135 | PNKP | 1.09 | 0.93-1.27 | 0.28 |
| B3GNT2 | 1.12 | 0.97-1.3 | 0.112 | POMP | 1.16 | 0.92-1.48 | 0.213 |
| BOD1L1 | 1.06 | 0.89-1.27 | 0.486 | PPIA | 0.98 | 0.83-1.16 | 0.839 |
| BZW1 | 1.08 | 0.9-1.3 | 0.387 | PPIB | 1.08 | 0.91-1.28 | 0.409 |
| C15orf48 | 1.15 | 1-1.35 | 0.061 | PPM1M | 1.26 | 1.04-1.53 | 0.017 |
| C1D | 1.16 | 0.97-1.4 | 0.108 | PPP1CA | 1.08 | 0.89-1.31 | 0.448 |

| CALR | 1.11 | 0.92-1.34 | 0.287 | PRELID1 | 1.06 | 0.86-1.31 | 0.569 |
| --- | --- | --- | --- | --- | --- | --- | --- |
| CAMP | 1.15 | 1.02-1.31 | 0.025 | PRKAB1 | 1.16 | 0.92-1.48 | 0.21 |
| CAPG | 1.31 | 1.1-1.58 | 0.003 | PRNP | 1 | 0.85-1.17 | 0.986 |
| CAPNS1 | 1.16 | 0.94-1.44 | 0.172 | PROK2 | 1.18 | 1.04-1.34 | 0.009 |
| CCDC88C | 0.91 | 0.76-1.08 | 0.277 | PSMA2 | 0.94 | 0.76-1.16 | 0.557 |
| CCND3 | 1.27 | 1.06-1.53 | 0.01 | PSMA4 | 1 | 0.84-1.2 | 0.96 |
| CCT2 | 0.95 | 0.8-1.13 | 0.584 | PSMA7 | 1.18 | 0.96-1.45 | 0.117 |
| CD164 | 1.09 | 0.92-1.3 | 0.315 | PSMB1 | 1.04 | 0.84-1.28 | 0.739 |
| CD177 | 1.21 | 1.12-1.31 | <0.001 | PSMB2 | 0.95 | 0.78-1.17 | 0.644 |
| CD38 | 0.96 | 0.85-1.09 | 0.53 | PSMB3 | 1.1 | 0.89-1.37 | 0.363 |
| CD47 | 0.9 | 0.74-1.1 | 0.314 | PSMB4 | 1.08 | 0.87-1.34 | 0.502 |
| CD63 | 1.43 | 1.16-1.78 | 0.001 | PSMB6 | 1.17 | 0.98-1.42 | 0.09 |
| CD81 | 0.9 | 0.77-1.05 | 0.192 | PSMC2 | 1.01 | 0.83-1.23 | 0.902 |
| CDADC1 | 1.37 | 1.13-1.66 | 0.001 | PSMC3 | 1.03 | 0.85-1.26 | 0.74 |
| CEACAM1 | 1.25 | 1.1-1.44 | 0.001 | PSMD13 | 1.09 | 0.88-1.36 | 0.43 |
| CEBPE | 1.1 | 0.97-1.26 | 0.142 | PSMD14 | 1 | 0.84-1.2 | 0.986 |
| CEP19 | 1.09 | 0.92-1.3 | 0.319 | PSMD6 | 1.24 | 0.97-1.58 | 0.09 |
| CHURC1 | 1.02 | 0.86-1.21 | 0.834 | PSME4 | 1.05 | 0.86-1.29 | 0.627 |
| CIB2 | 0.97 | 0.83-1.13 | 0.687 | PYCARD | 1.1 | 0.94-1.3 | 0.225 |
| CKAP4 | 1.56 | 1.31-1.88 | <0.001 | PYGL | 1.33 | 1.13-1.56 | 0.001 |
| CLEC12A | 1.01 | 0.88-1.17 | 0.842 | RAB24 | 1.19 | 1-1.41 | 0.047 |
| CLEC4A | 1.15 | 0.94-1.41 | 0.18 | RAB3D | 1.19 | 0.98-1.44 | 0.078 |
| CLTC | 1.21 | 0.99-1.49 | 0.066 | RAB6A | 1.13 | 0.92-1.39 | 0.246 |
| CMAS | 1.08 | 0.88-1.34 | 0.464 | RANBP9 | 1.29 | 1.06-1.59 | 0.014 |
| CMPK1 | 0.92 | 0.78-1.08 | 0.306 | RASA2 | 1.12 | 0.96-1.31 | 0.135 |
| CNN2 | 1.25 | 1.04-1.52 | 0.021 | RBFA | 0.92 | 0.78-1.08 | 0.314 |
| COMMD1 | 1 | 0.82-1.22 | 0.994 | RBM3 | 0.96 | 0.82-1.12 | 0.618 |
| COPE | 1.09 | 0.92-1.3 | 0.296 | REEP5 | 1.09 | 0.87-1.38 | 0.461 |
| COX4I1 | 1.01 | 0.82-1.25 | 0.902 | RINL | 1.11 | 0.91-1.35 | 0.305 |
| COX5A | 0.98 | 0.82-1.18 | 0.848 | RNASEH2C | 0.98 | 0.82-1.17 | 0.812 |
| COX6A1 | 1.15 | 0.94-1.42 | 0.181 | RNF10 | 1.51 | 1.23-1.87 | <0.001 |
| COX7B | 1.11 | 0.94-1.32 | 0.206 | RNF20 | 1 | 0.79-1.28 | 0.979 |
| CPNE3 | 1.13 | 0.94-1.36 | 0.189 | RPL10 | 0.9 | 0.76-1.07 | 0.224 |
| CRISPLD2 | 1.19 | 1.02-1.39 | 0.027 | RPL10A | 0.82 | 0.67-0.99 | 0.037 |
| CSNK2A1 | 1.02 | 0.81-1.29 | 0.866 | RPL11 | 0.97 | 0.81-1.17 | 0.75 |
| CTNNA1 | 1.3 | 1.04-1.64 | 0.024 | RPL12 | 0.91 | 0.74-1.12 | 0.37 |
| CTSC | 1.01 | 0.82-1.25 | 0.924 | RPL13 | 0.94 | 0.81-1.11 | 0.477 |
| CUEDC2 | 1.1 | 0.91-1.34 | 0.321 | RPL14 | 0.83 | 0.68-1.02 | 0.073 |
| CYB5R4 | 1.09 | 0.89-1.33 | 0.391 | RPL15 | 0.94 | 0.79-1.11 | 0.457 |
| CYBA | 1.18 | 1-1.41 | 0.053 | RPL19 | 0.84 | 0.68-1.02 | 0.086 |
| CYBB | 1.08 | 0.89-1.3 | 0.446 | RPL24 | 1 | 0.83-1.21 | 0.978 |
| CYFIP2 | 1.09 | 0.89-1.35 | 0.404 | RPL28 | 0.88 | 0.73-1.07 | 0.199 |
| DACH1 | 1.29 | 1.13-1.48 | <0.001 | RPL3 | 0.89 | 0.75-1.05 | 0.159 |
| DBNL | 1.19 | 0.95-1.49 | 0.125 | RPL36AL | 0.99 | 0.79-1.23 | 0.894 |
| DCTN3 | 1.05 | 0.86-1.29 | 0.63 | RPL4 | 0.89 | 0.73-1.07 | 0.221 |
| DDX46 | 1.01 | 0.82-1.24 | 0.954 | RPL6 | 0.99 | 0.83-1.17 | 0.868 |
| DEGS1 | 1.19 | 0.96-1.49 | 0.115 | RPL7 | 1.03 | 0.89-1.19 | 0.67 |
| DHRS1 | 1.13 | 0.94-1.36 | 0.194 | RPL7A | 0.94 | 0.78-1.14 | 0.539 |
| DHRS7 | 1.2 | 0.97-1.49 | 0.098 | RPL8 | 0.98 | 0.82-1.17 | 0.824 |
| DHRS9 | 1.22 | 1.05-1.42 | 0.009 | RPLP0 | 1 | 0.86-1.15 | 0.952 |
| DOCK8 | 1.04 | 0.87-1.25 | 0.664 | RPS15 | 0.96 | 0.79-1.15 | 0.635 |
| DSTN | 0.88 | 0.71-1.09 | 0.255 | RPS17 | 0.95 | 0.8-1.13 | 0.565 |
| EEF1A1 | 1 | 0.85-1.17 | 0.954 | RPS18 | 0.95 | 0.82-1.1 | 0.505 |
| EIF3C | 1 | 0.84-1.19 | 0.979 | RPS19 | 0.92 | 0.78-1.09 | 0.344 |
| EIF3E | 0.92 | 0.77-1.09 | 0.333 | RPS2 | 0.99 | 0.85-1.14 | 0.856 |
| EIF3H | 0.84 | 0.67-1.06 | 0.138 | RPS20 | 0.9 | 0.75-1.09 | 0.293 |
| EIF3K | 0.95 | 0.77-1.17 | 0.606 | RPS24 | 1.03 | 0.89-1.19 | 0.689 |
| EIF3M | 0.87 | 0.7-1.06 | 0.174 | RPS25 | 0.96 | 0.79-1.16 | 0.689 |

| EIF4E3 | 1.2 | 0.98-1.46 | 0.08 | RPS3 | 0.92 | 0.77-1.11 | 0.378 |
| --- | --- | --- | --- | --- | --- | --- | --- |
| EIF4G2 | 1.1 | 0.9-1.34 | 0.347 | RPS4Y1 | 1.03 | 0.98-1.09 | 0.212 |
| EIF5A | 0.95 | 0.78-1.16 | 0.617 | RPS5 | 0.92 | 0.78-1.08 | 0.311 |
| EIF5B | 0.92 | 0.77-1.1 | 0.374 | RPS6 | 0.93 | 0.78-1.11 | 0.445 |
| EMP3 | 1.01 | 0.84-1.23 | 0.883 | RPS6KA1 | 1.23 | 1.01-1.49 | 0.04 |
| ETFB | 1.03 | 0.85-1.25 | 0.734 | RPS8 | 0.94 | 0.79-1.12 | 0.49 |
| ETHE1 | 1.11 | 0.93-1.32 | 0.247 | RPSA | 0.99 | 0.86-1.14 | 0.855 |
| EXOC3 | 1.14 | 0.91-1.42 | 0.26 | RSBN1L | 0.92 | 0.74-1.13 | 0.423 |
| F5 | 1.31 | 1.13-1.53 | 0.001 | RSU1 | 1.12 | 0.86-1.45 | 0.404 |
| FAM107B | 1.12 | 0.94-1.34 | 0.201 | S100A13 | 1.04 | 0.88-1.23 | 0.667 |
| FAR1 | 1.11 | 0.96-1.28 | 0.175 | S100A8 | 1.34 | 1.18-1.54 | <0.001 |
| FCHO2 | 1.05 | 0.91-1.2 | 0.517 | S100A9 | 1.39 | 1.18-1.65 | <0.001 |
| FERMT3 | 1.29 | 1.08-1.54 | 0.004 | S1PR4 | 1.13 | 0.99-1.28 | 0.063 |
| FGFR1OP2 | 1.1 | 0.92-1.32 | 0.307 | SARNP | 1.13 | 0.94-1.36 | 0.188 |
| FIS1 | 1.19 | 0.95-1.5 | 0.129 | SCP2 | 0.95 | 0.78-1.17 | 0.66 |
| FLNA | 1.14 | 0.96-1.36 | 0.129 | SCRG1 | 1.14 | 0.95-1.37 | 0.178 |
| FLOT1 | 1.34 | 1.13-1.61 | 0.001 | SDHB | 1.14 | 0.9-1.44 | 0.265 |
| FLOT2 | 1.28 | 1.08-1.52 | 0.005 | SEC11C | 1.03 | 0.9-1.18 | 0.7 |
| FMO5 | 0.94 | 0.79-1.11 | 0.472 | SEC61B | 1.13 | 0.94-1.37 | 0.203 |
| G6PD | 1.29 | 1.07-1.57 | 0.008 | SEC61G | 1.15 | 0.96-1.37 | 0.129 |
| GAPDH | 1.3 | 1.07-1.58 | 0.009 | SERBP1 | 0.96 | 0.77-1.19 | 0.687 |
| GAS7 | 1.37 | 1.15-1.63 | <0.001 | SERPINB1 | 1.41 | 1.17-1.72 | <0.001 |
| GATAD1 | 0.96 | 0.78-1.17 | 0.668 | SFR1 | 0.99 | 0.82-1.19 | 0.92 |
| GCA | 1.22 | 1.05-1.43 | 0.01 | SH3BGRL3 | 1.1 | 0.9-1.34 | 0.371 |
| GDE1 | 1.15 | 0.89-1.48 | 0.297 | SLC25A24 | 1.12 | 0.96-1.3 | 0.149 |
| GLRX | 1.18 | 0.96-1.46 | 0.117 | SLC27A4 | 1.04 | 0.88-1.23 | 0.659 |
| GLUD1 | 0.96 | 0.79-1.18 | 0.714 | SLK | 1.13 | 0.95-1.35 | 0.157 |
| GLYR1 | 1.16 | 0.92-1.45 | 0.209 | SMDT1 | 0.93 | 0.76-1.12 | 0.437 |
| GNG10 | 1.56 | 1.24-1.99 | <0.001 | SMIM14 | 0.99 | 0.81-1.22 | 0.941 |
| GOLIM4 | 1.01 | 0.86-1.19 | 0.914 | SMIM7 | 0.98 | 0.79-1.21 | 0.865 |
| GPI | 1.12 | 0.93-1.35 | 0.249 | SNAPC5 | 0.95 | 0.77-1.17 | 0.641 |
| GPR84 | 1.35 | 1.2-1.52 | <0.001 | SNRPD3 | 1 | 0.81-1.24 | 0.985 |
| GRK6 | 1.29 | 1.08-1.56 | 0.007 | SNX4 | 1.07 | 0.9-1.27 | 0.447 |
| GRN | 1.21 | 1.04-1.43 | 0.017 | SPCS1 | 0.97 | 0.79-1.18 | 0.725 |
| HDLBP | 1.18 | 0.94-1.48 | 0.156 | SPCS2 | 1.08 | 0.89-1.33 | 0.422 |
| HHEX | 1.19 | 0.98-1.45 | 0.075 | SRI | 1.11 | 0.87-1.42 | 0.416 |
| HIPK1 | 1.16 | 0.98-1.37 | 0.085 | SRP19 | 1 | 0.81-1.25 | 0.966 |
| HJURP | 1.05 | 0.94-1.16 | 0.388 | SRP72 | 0.92 | 0.76-1.1 | 0.363 |
| HK3 | 1.4 | 1.21-1.64 | <0.001 | SRP9 | 1.03 | 0.89-1.19 | 0.706 |
| HNRNPA2B1 | 1.05 | 0.83-1.34 | 0.687 | SRSF3 | 0.95 | 0.77-1.16 | 0.609 |
| HNRNPD | 0.95 | 0.78-1.16 | 0.631 | SRSF9 | 1.13 | 0.9-1.42 | 0.284 |
| HNRNPF | 1.03 | 0.82-1.28 | 0.82 | SSR3 | 1.04 | 0.87-1.25 | 0.643 |
| HP | 1.37 | 1.22-1.55 | <0.001 | SSR4 | 1.03 | 0.87-1.21 | 0.733 |
| HSD11B1 | 0.99 | 0.81-1.23 | 0.958 | STIM1 | 1.19 | 0.96-1.47 | 0.111 |
| HSP90B1 | 1.07 | 0.93-1.25 | 0.346 | STIM2 | 0.9 | 0.75-1.09 | 0.283 |
| IKZF1 | 0.98 | 0.78-1.22 | 0.834 | STK16 | 1.28 | 1.04-1.58 | 0.022 |
| IL16 | 1.01 | 0.82-1.25 | 0.93 | STK4 | 1.03 | 0.84-1.26 | 0.763 |
| IL17RA | 1.3 | 1.09-1.56 | 0.005 | STXBP2 | 1.31 | 1.11-1.55 | 0.001 |
| ILK | 1.16 | 0.91-1.48 | 0.22 | STXBP5 | 1.1 | 0.96-1.27 | 0.167 |
| INHBA | 1.18 | 1.05-1.34 | 0.006 | SUGT1 | 0.93 | 0.74-1.17 | 0.557 |
| IQGAP2 | 1.01 | 0.85-1.19 | 0.917 | SUMO2 | 1.03 | 0.82-1.29 | 0.795 |
| ITGAM | 1.45 | 1.21-1.74 | <0.001 | SUN2 | 1.05 | 0.88-1.25 | 0.575 |
| ITGB2 | 1.28 | 1.06-1.54 | 0.01 | SYNE1 | 1.19 | 1.01-1.42 | 0.042 |
| KCTD12 | 0.97 | 0.83-1.13 | 0.709 | TACC1 | 0.95 | 0.76-1.18 | 0.637 |
| KIAA0513 | 1.09 | 0.9-1.33 | 0.384 | TBC1D2 | 1.23 | 1.02-1.48 | 0.029 |
| KLHDC4 | 0.94 | 0.8-1.09 | 0.397 | TBC1D8 | 1.46 | 1.24-1.74 | <0.001 |
| KLHL6 | 1.11 | 0.9-1.37 | 0.321 | TBCA | 1.2 | 0.99-1.45 | 0.063 |
| LAMTOR4 | 0.99 | 0.83-1.19 | 0.942 | TBCB | 1.13 | 0.91-1.39 | 0.262 |

| LASP1 | 1.18 | 0.95-1.48 | 0.137 | TCP11L2 | 1.2 | 1-1.45 | 0.054 |
| --- | --- | --- | --- | --- | --- | --- | --- |
| LBR | 1.13 | 0.97-1.32 | 0.108 | TECR | 0.97 | 0.83-1.14 | 0.742 |
| LCN2 | 1.22 | 1.1-1.35 | <0.001 | THBS1 | 1.26 | 1.1-1.45 | 0.001 |
| LDHA | 1.5 | 1.23-1.85 | <0.001 | THRAP3 | 1.05 | 0.85-1.31 | 0.623 |
| LDHC | 0.98 | 0.77-1.25 | 0.864 | TIRAP | 1.28 | 1.04-1.59 | 0.023 |
| LGALS3 | 1.1 | 0.88-1.38 | 0.395 | TKT | 1.2 | 1-1.43 | 0.049 |
| LILRB3 | 1.28 | 1.09-1.52 | 0.003 | TLN1 | 1.28 | 1.07-1.55 | 0.009 |
| LIMD2 | 1.01 | 0.86-1.19 | 0.869 | TM9SF3 | 0.98 | 0.81-1.18 | 0.82 |
| LIPG | 0.73 | 0.33-1.53 | 0.405 | TMED2 | 1.06 | 0.86-1.3 | 0.586 |
| LMO4 | 1.09 | 0.87-1.37 | 0.463 | TMED9 | 1.09 | 0.92-1.3 | 0.311 |
| LRRFIP1 | 1.07 | 0.88-1.32 | 0.498 | TMEM120A | 1.23 | 1.06-1.45 | 0.009 |
| LTA4H | 1.09 | 0.88-1.34 | 0.428 | TMEM160 | 1.03 | 0.91-1.16 | 0.646 |
| LTB4R | 1.33 | 1.1-1.62 | 0.004 | TMEM256 | 1.04 | 0.9-1.2 | 0.575 |
| LTF | 1.2 | 1.1-1.33 | <0.001 | TMEM258 | 1.01 | 0.83-1.22 | 0.935 |
| MAN2A1 | 0.94 | 0.81-1.1 | 0.443 | TMEM30A | 1.05 | 0.89-1.24 | 0.568 |
| MAP1LC3A | 1.14 | 0.99-1.3 | 0.063 | TMEM40 | 0.99 | 0.85-1.16 | 0.901 |
| MAP2K4 | 1.02 | 0.85-1.22 | 0.836 | TMX3 | 0.98 | 0.85-1.13 | 0.823 |
| MAPK13 | 1.16 | 0.98-1.37 | 0.088 | TNKS2 | 1.01 | 0.84-1.21 | 0.93 |
| MDH2 | 0.94 | 0.76-1.16 | 0.576 | TP53INP2 | 1.04 | 0.87-1.25 | 0.654 |
| MED10 | 0.92 | 0.74-1.15 | 0.468 | TPM3 | 1.21 | 0.93-1.57 | 0.151 |
| METAP2 | 0.93 | 0.77-1.12 | 0.443 | TPR | 1.05 | 0.87-1.28 | 0.592 |
| METTL9 | 1.52 | 1.25-1.88 | <0.001 | TRAK2 | 0.99 | 0.81-1.2 | 0.906 |
| MGST2 | 1.16 | 0.94-1.44 | 0.176 | TRAM1 | 0.96 | 0.81-1.15 | 0.685 |
| MMP25 | 1.18 | 1.04-1.34 | 0.01 | TRAPPC4 | 1.02 | 0.83-1.26 | 0.846 |
| MMP8 | 1.26 | 1.15-1.38 | <0.001 | TRIOBP | 1.16 | 0.96-1.4 | 0.127 |
| MMP9 | 1.34 | 1.2-1.5 | <0.001 | TSC22D4 | 1.15 | 0.98-1.36 | 0.081 |
| MPC2 | 1.13 | 0.88-1.45 | 0.332 | TUBA4A | 1.26 | 1.05-1.51 | 0.015 |
| MRPL20 | 0.96 | 0.79-1.18 | 0.717 | TXNDC17 | 1.2 | 1-1.46 | 0.057 |
| MRPS14 | 0.92 | 0.76-1.12 | 0.417 | TXNDC9 | 1.03 | 0.87-1.22 | 0.747 |
| MRPS18C | 1.09 | 0.89-1.34 | 0.402 | UQCRB | 1.1 | 0.94-1.3 | 0.245 |
| MSRA | 1.38 | 1.13-1.69 | 0.002 | VCL | 1.12 | 0.91-1.39 | 0.296 |
| MTMR3 | 1.33 | 1.1-1.62 | 0.003 | VCP | 1.2 | 0.99-1.47 | 0.07 |
| MYH9 | 1.27 | 1.07-1.53 | 0.008 | VDAC2 | 1.09 | 0.88-1.35 | 0.43 |
| MYL6 | 1.38 | 1.13-1.7 | 0.002 | VDAC3 | 0.99 | 0.79-1.25 | 0.952 |
| MYO5A | 1.08 | 0.91-1.29 | 0.365 | WDR1 | 1.21 | 0.96-1.53 | 0.103 |
| NABP1 | 1.14 | 0.97-1.34 | 0.119 | WDR26 | 1.14 | 0.94-1.39 | 0.195 |
| NACA | 0.93 | 0.72-1.19 | 0.548 | YBX1 | 1.08 | 0.85-1.38 | 0.514 |
| NAP1L4 | 0.99 | 0.8-1.23 | 0.935 | YEATS4 | 0.97 | 0.83-1.14 | 0.713 |
| NAPSA | 1.08 | 0.94-1.26 | 0.287 | YWHAB | 1.05 | 0.82-1.36 | 0.688 |
| NCF1 | 1.09 | 0.91-1.29 | 0.351 | YWHAE | 1.06 | 0.83-1.35 | 0.637 |
| NCOA4 | 0.98 | 0.81-1.18 | 0.844 | YWHAZ | 1.16 | 0.93-1.47 | 0.194 |
| NDUFA1 | 1.28 | 1.04-1.57 | 0.019 | ZFHX3 | 0.96 | 0.82-1.11 | 0.571 |
| NDUFA11 | 1.05 | 0.9-1.22 | 0.528 |  |  |  |  |
