# Supplementary material for: S100A8/A9hi neutrophils induce mitochondrial dysfunction and PANoptosis in endothelial cells via mitochondrial complex I deficiency during sepsis
Source: Cell Death Dis. 2024 Jun 28;15(6):462. doi: 10.1038/s41419-024-06849-6 (PMC11213914; doi:10.1038/s41419-024-06849-6)
Supplement: Supplementary file 3 — Original Data File [file 41419_2024_6849_MOESM3_ESM.docx]

**Figure. 2G**


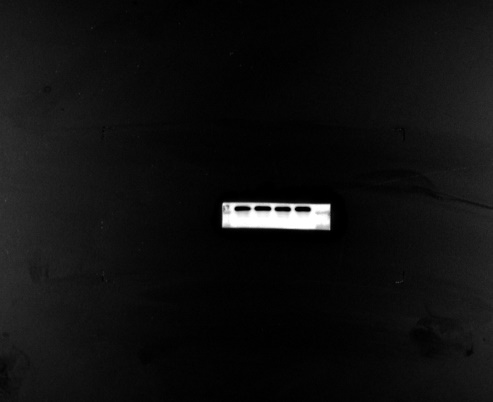


**GAPDH**

40

35


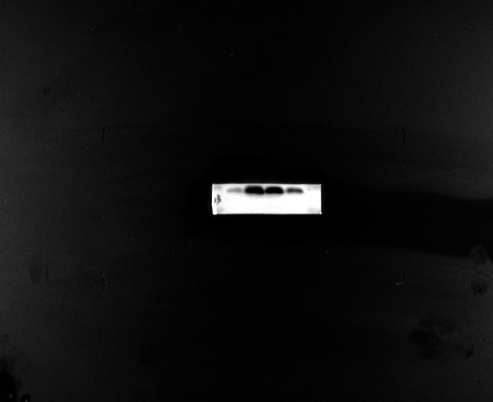


**S100a8+S100a9**

15

10

**Figure. 3G**


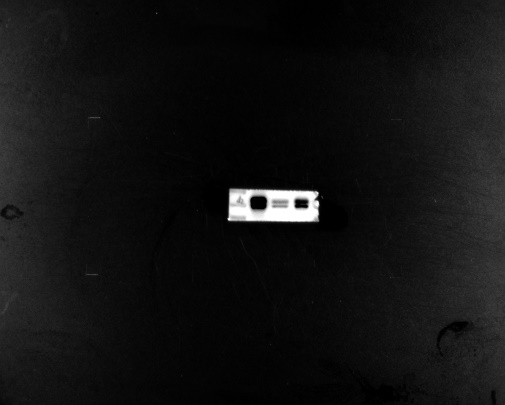


**P-Erk**

40

50


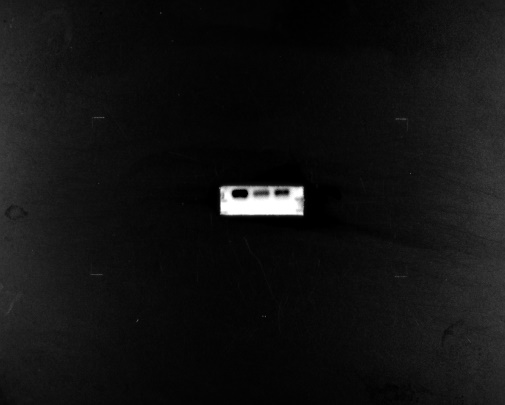


**P-MEK**

40

50


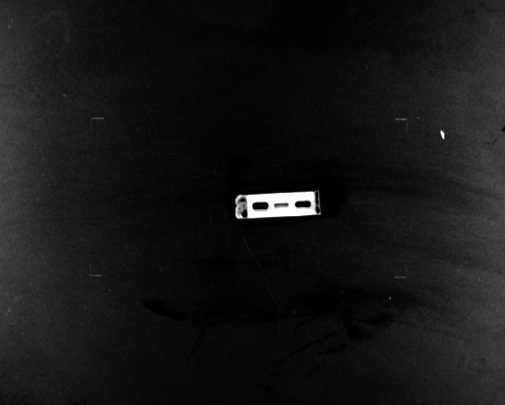


**Nrf1**

50

70


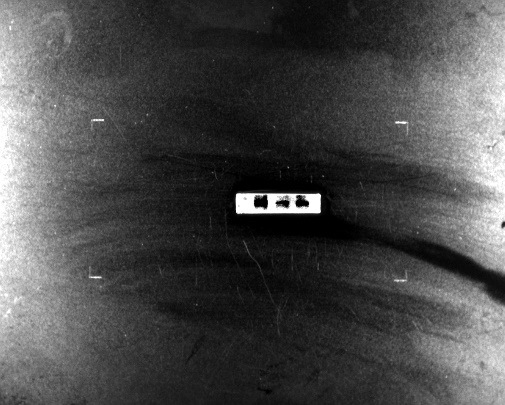


**PGC-1α**

100

150


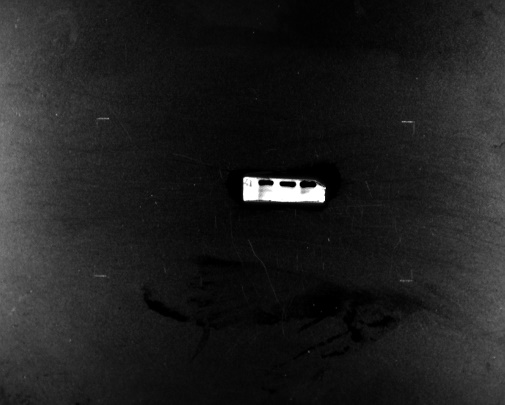


**GAPDH**

35

40

**Figure. 3J**


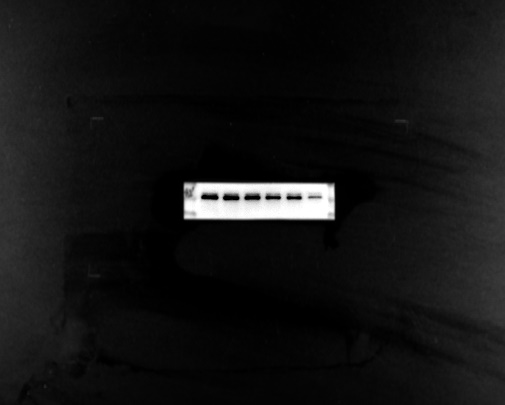


**P-Erk**

50

40


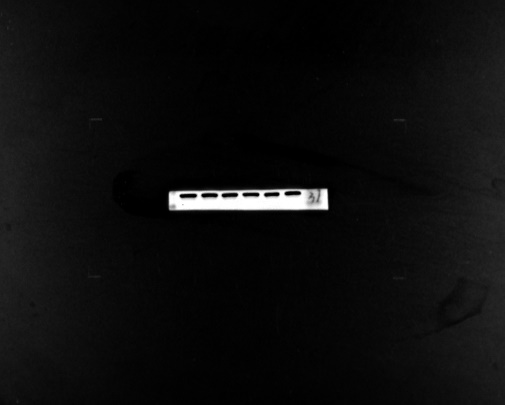


**GAPDH**

40

35


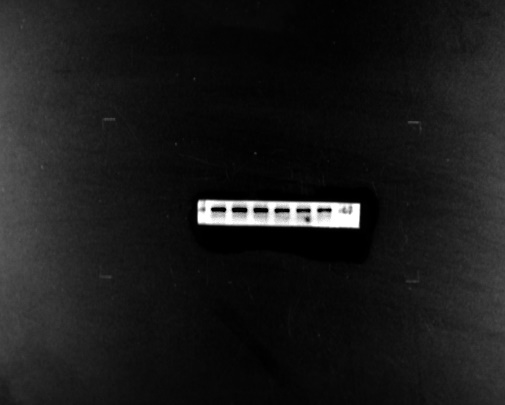


**Nrf1**

50

70


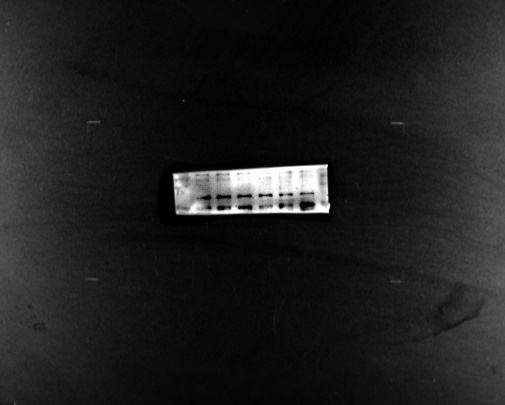


**PGC-1α**

100

150


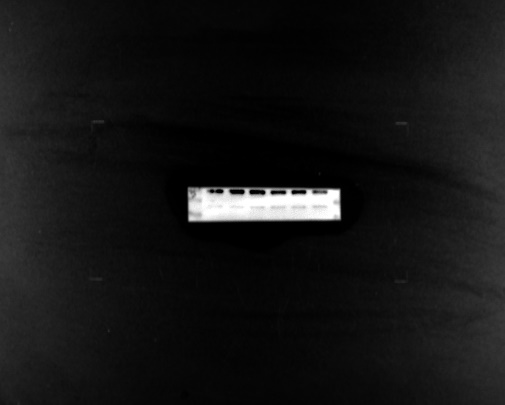


**P-MEK**

50

40

**Figure. 3N**


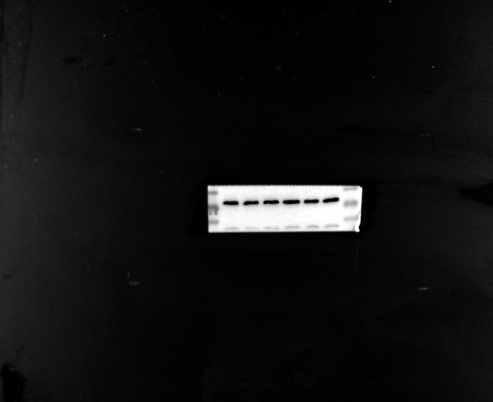


**Complex II**

20

35


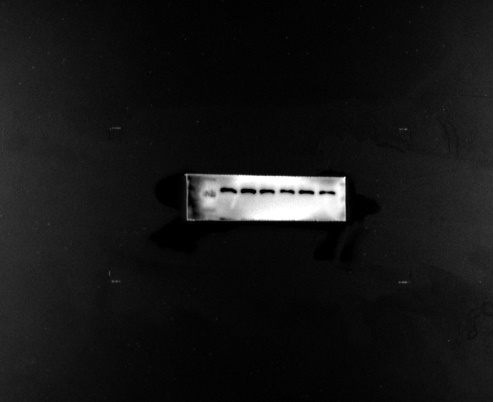


**Complex I**

20

25


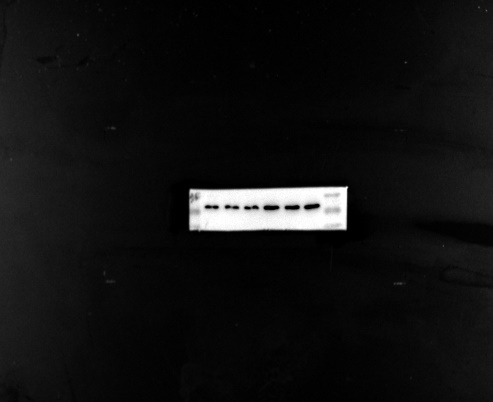


**Complex IV**

35

40


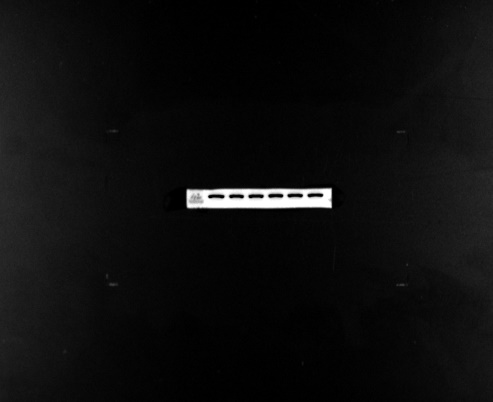


**β-Actin**

40

50


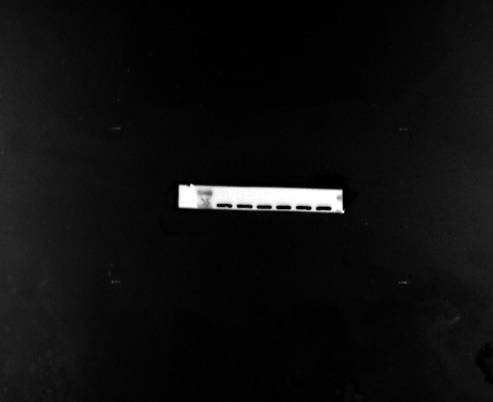


**Complex V**

50

70


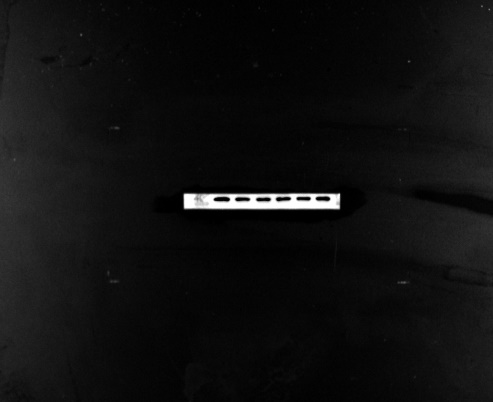


**Complex III**

40

50

**Figure. 4A**


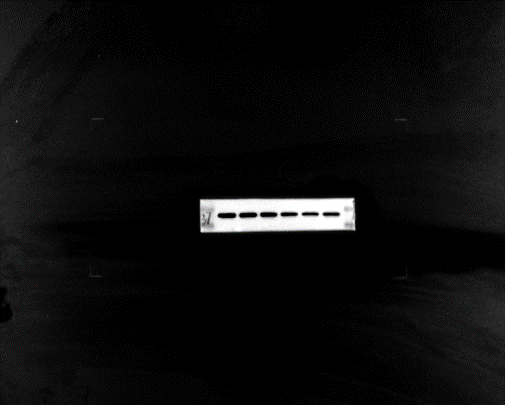


**GAPDH**

35

40


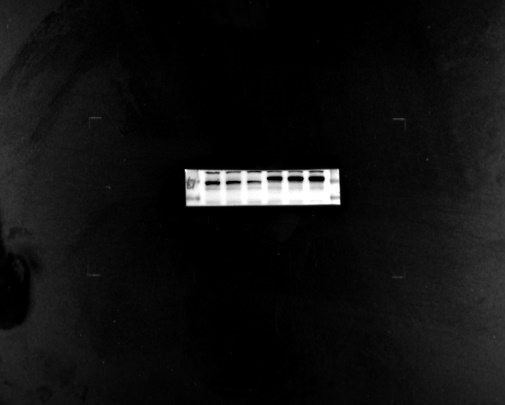


**Nrf1**

50

70

**Figure. 4J**


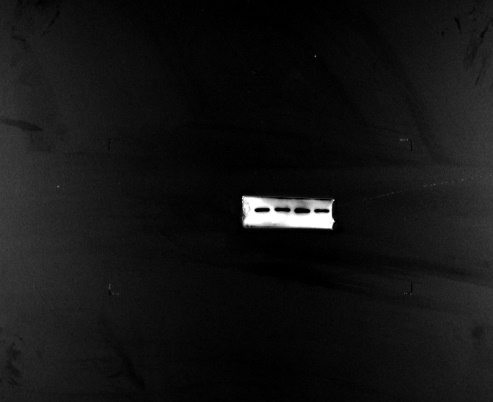


**GAPDH**

40

35


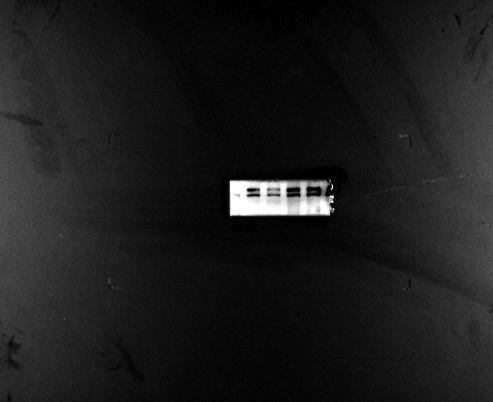


**Sirt1**

100

150

**Figure. 4L**


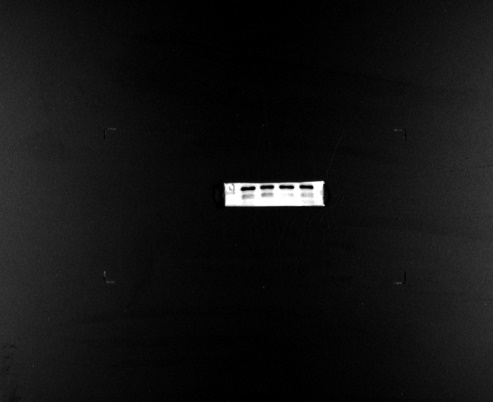


**GAPDH**

35

40


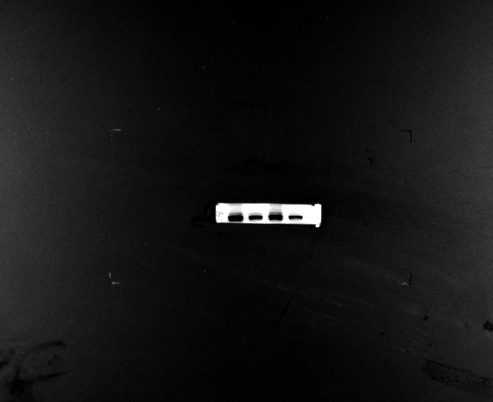


**Sirt1**

150

100

**Figure. 5A**


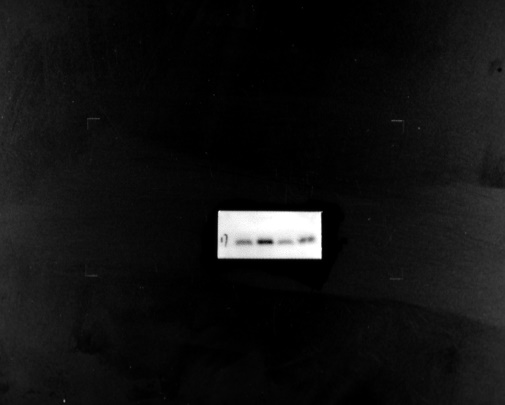


**Fis1**

15

20


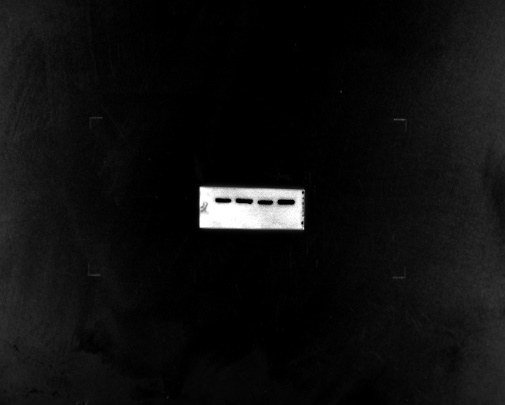


**GAPDH**

35

40


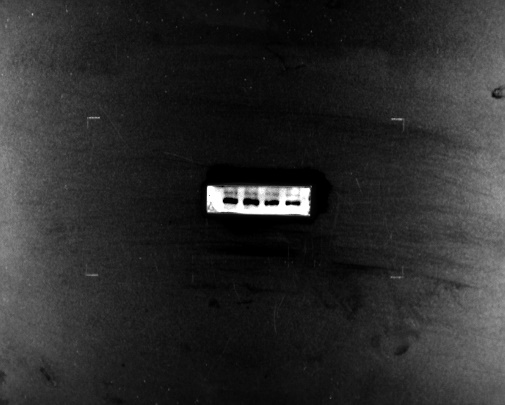


**p-Drp**

70

100

**Figure. 5B**


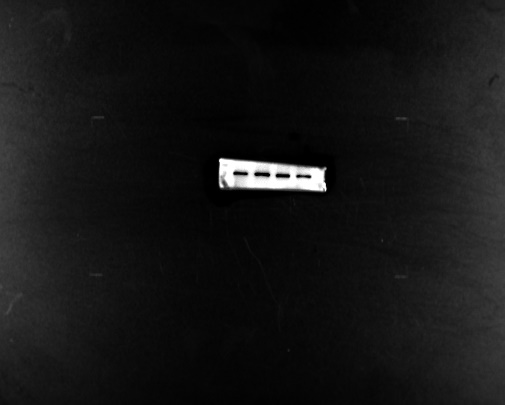


**Mfn2**

100

70


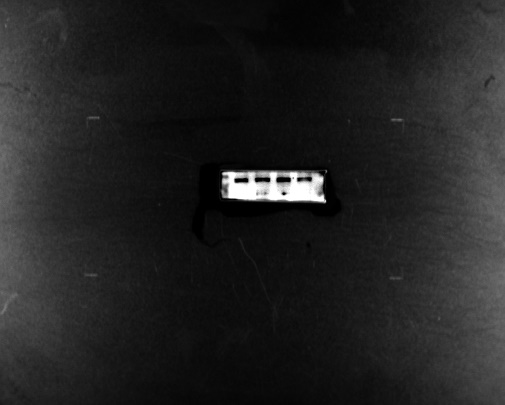


**Mfn1**

70

100


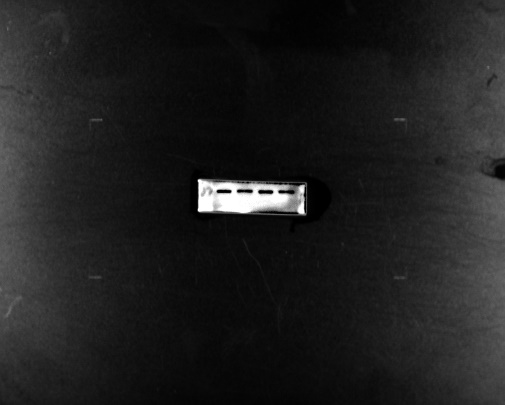


**GAPDH**

35

40

**Figure. 5C**


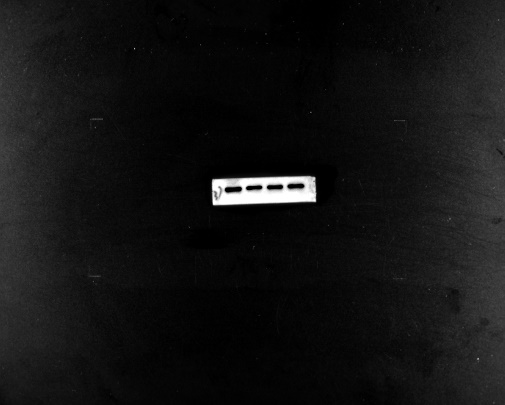


**GAPDH**

35

40


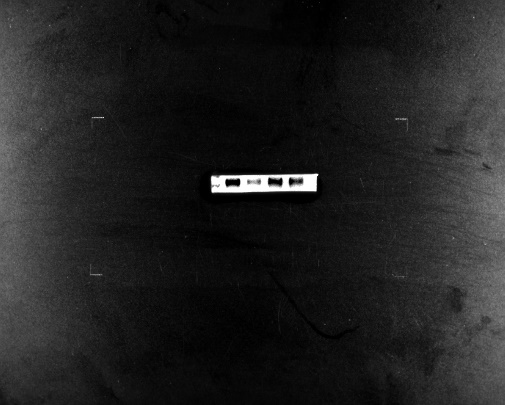


**LAMP1**

100

150


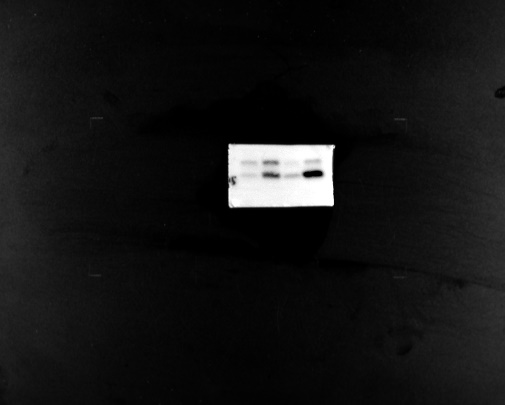


**LC3BI**

**LC3BII**

10

20

**Figure. 5H**


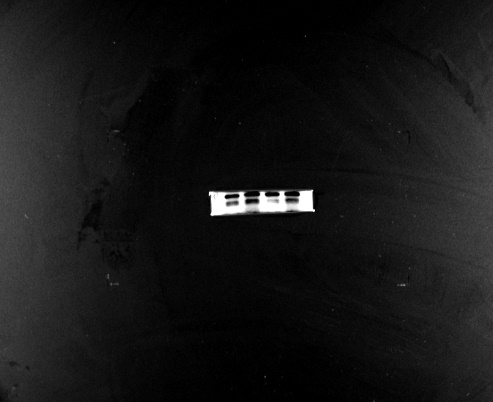


**GAPDH**

35

40


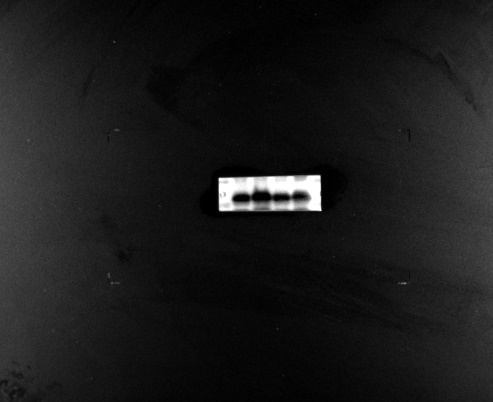


**Fis1**

15

20

**Figure. 6B**


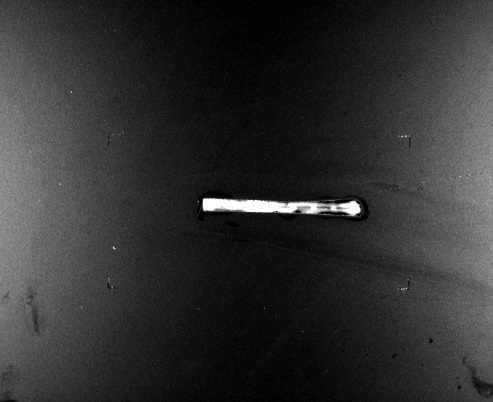


**ZBP1**

40

50


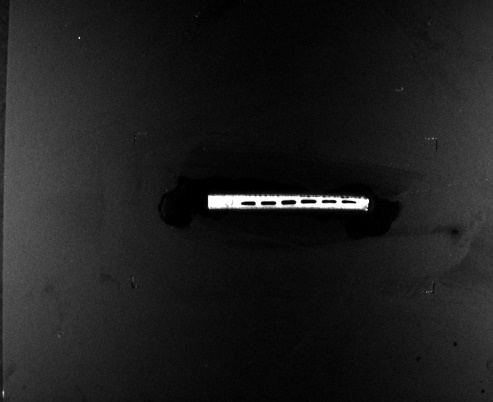


**GAPDH**

35

40

**Figure. 6E**


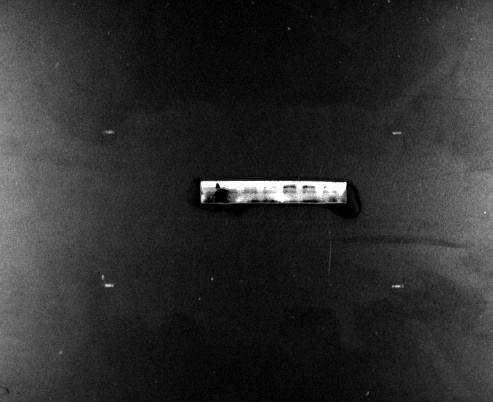


**p-MLKL**

50

70


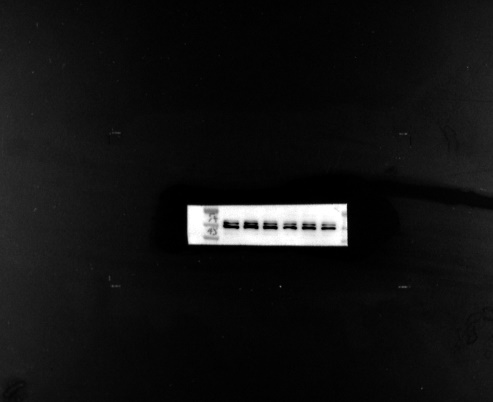


**MLKL**

40

70


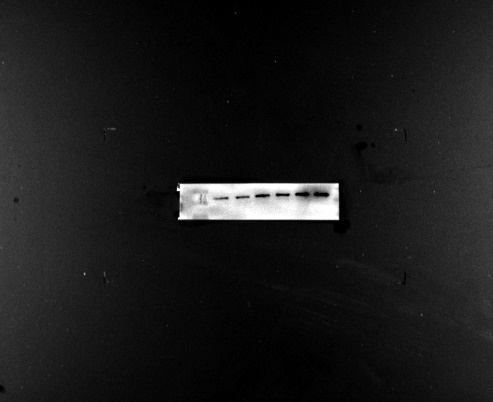


**N-terminal GSDMD**

35

40


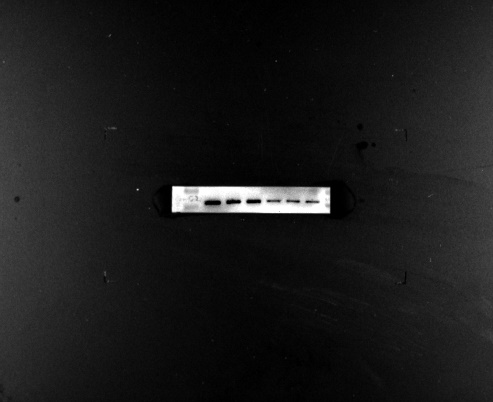


**GSDMD**

50

70


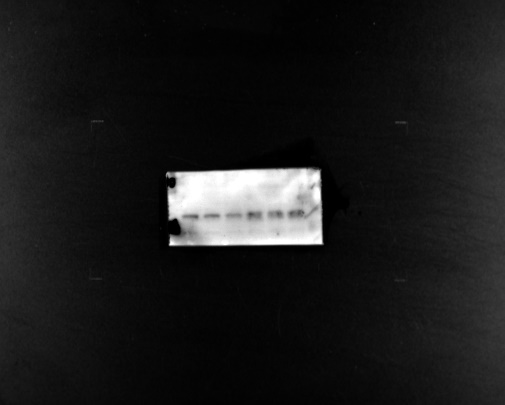


**Cleaved Caspase 3**

15

20


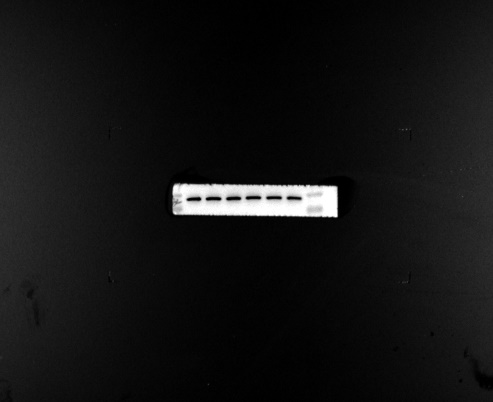


**Caspase 3**

25

35


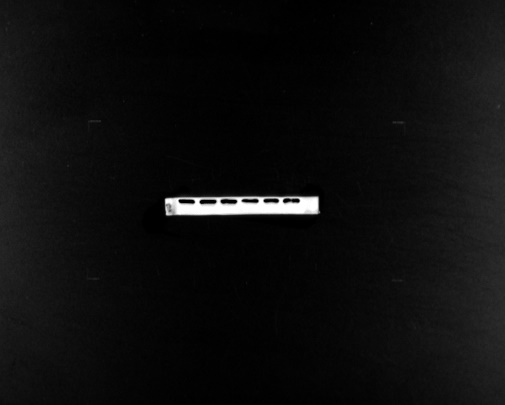


**GAPDH**

35

40

**Figure. 6G**


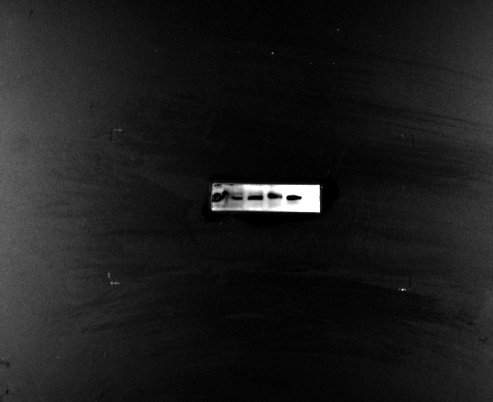


**p-MLKL**

70

50


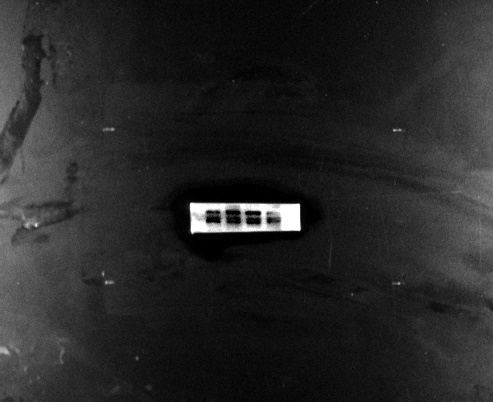


**MLKL**

70

50


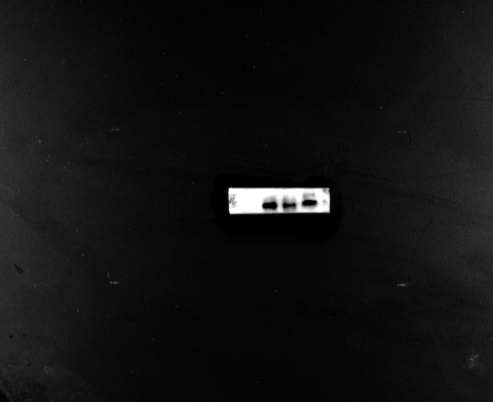


**N-terminal GSDMD**

35

40


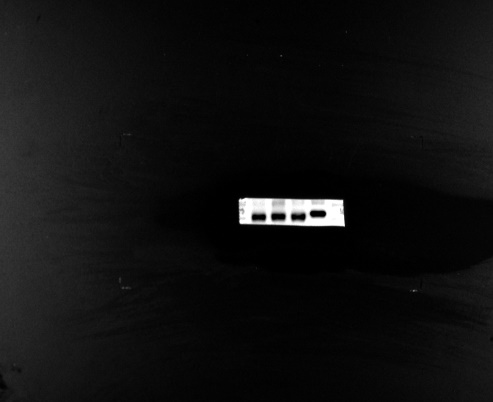


**GSDMD**

70

50


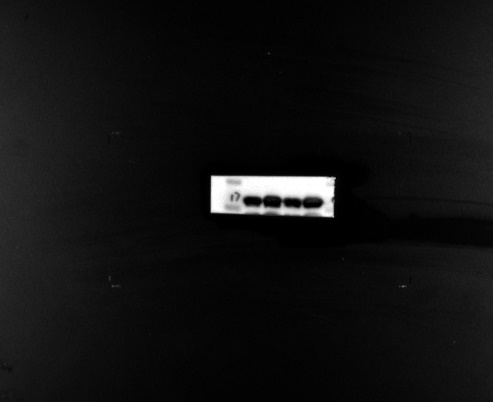


**Cleaved Caspase 3**

20

15


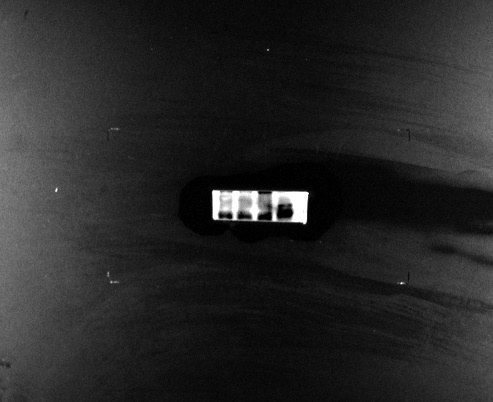


**Caspase 3**

35

25


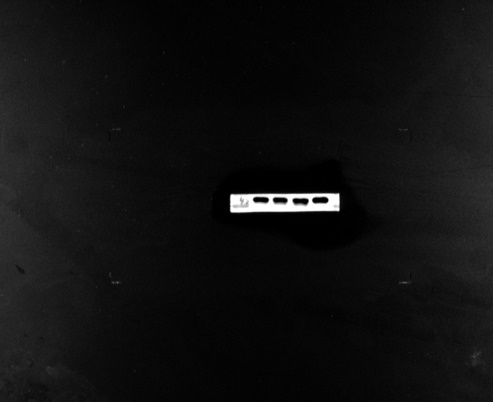


**β-Actin**

50

40
